# Uncovering the Genomic Regions Associated with Yield Maintenance in Rice Under Drought Stress Using an Integrated Meta-Analysis Approach

**DOI:** 10.1186/s12284-024-00684-1

**Published:** 2024-01-16

**Authors:** Parisa Daryani, Nazanin Amirbakhtiar, Jahad Soorni, Fatemeh Loni, Hadi Darzi Ramandi, Zahra-Sadat Shobbar

**Affiliations:** 1 Department of Systems Biology, Agricultural Biotechnology Research Institute of Iran (ABRII), Agricultural Research, Education and Extension Organization (AREEO), Karaj, Iran; 2 National Plant Gene Bank of Iran, Seed and Plant Improvement Institute (SPII), Agricultural Research, Education and Extension Organization (AREEO), Karaj, Iran; 3 Department of Plant Production and Genetics, Faculty of Agriculture, Bu-Ali Sina University, Hamedan, Iran

**Keywords:** Breeding MQTLs, Drought tolerance, Drought-responsive genes, Meta-analysis of QTLs, *Oryza sativa*, Genome-wide association studies

## Abstract

The complex trait of yield is controlled by several quantitative trait loci (QTLs). Given the global water deficit issue, the development of rice varieties suitable for non-flooded cultivation holds significant importance in breeding programs. The powerful approach of Meta-QTL (MQTL) analysis can be used for the genetic dissection of complicated quantitative traits. In the current study, a comprehensive MQTL analysis was conducted to identify consistent QTL regions associated with drought tolerance and yield-related traits under water deficit conditions in rice. In total, 1087 QTLs from 134 rice populations, published between 2000 to 2021, were utilized in the analysis. Distinct MQTL analysis of the relevant traits resulted in the identification of 213 stable MQTLs. The confidence interval (CI) for the detected MQTLs was between 0.12 and 19.7 cM. The average CI of the identified MQTLs (4.68 cM) was 2.74 times narrower compared to the average CI of the initial QTLs. Interestingly, 63 MQTLs coincided with SNP peak positions detected by genome-wide association studies for yield and drought tolerance-associated traits under water deficit conditions in rice. Considering the genes located both in the QTL-overview peaks and the SNP peak positions, 19 novel candidate genes were introduced, which are associated with drought response index, plant height, panicle number, biomass, and grain yield. Moreover, an inclusive MQTL analysis was performed on all the traits to obtain “Breeding MQTLs”. This analysis resulted in the identification of 96 MQTLs with a CI ranging from 0.01 to 9.0 cM. The mean CI of the obtained MQTLs (2.33 cM) was 4.66 times less than the mean CI of the original QTLs. Thirteen MQTLs fulfilling the criteria of having more than 10 initial QTLs, CI < 1 cM, and an average phenotypic variance explained greater than 10%, were designated as “Breeding MQTLs”. These findings hold promise for assisting breeders in enhancing rice yield under drought stress conditions.

**Supplementary Information:**

The online version contains supplementary material available at 10.1186/s12284-024-00684-1.

## Key message

Meta-QTLs associated with yield-related traits under drought stress were identified in rice applying an integrated meta-analysis approach, which will be useful in molecular breeding of rice to improve drought tolerance.

**Supplementary Information:**

The online version contains supplementary material available at 10.1186/s12284-024-00684-1.

## Introduction

To meet the global food requirements by 2050, an average annual increase of 44 million tons in food production is necessary (Tester and Langridge 2010). The scarcity of water resources has exacerbated the food shortage situation, and enhancing the drought tolerance (DT) of crops is an effective method to ensure food security (Hu and Xiong 2014). Rice (*Oryza sativa* L.) not only feeds more than half of the world's population but is also an important model plant in cereals (Xing and Zhang 2010). Drought stress is a main abiotic stress which restricts rice growth and productivity (Singhal et al. 2016). So, enhancing drought tolerance in rice is of great importance.

Enhancing DT in crops is a challenging process, given its complexity, which involves various physiological and molecular responses influenced by multiple alleles with minor effects (Blum 2011; Fukao and Xiong 2013). To comprehend the genetic basis of DT in rice, researchers have utilized quantitative trait locus (QTL) mapping with recombinant inbred lines (RILs) populations (Yue et al. 2006). Yield-associated traits and visible scores for plant performance during or after water deficit conditions are commonly used to assess DT, however; a limited number of QTLs were repetitively identified in various populations or different years or environments. Drought stress significantly reduces grain yield (GY), and identifying trustworthy loci related to DT using GY is challenging, considering that grain yield is influenced by many genes with minor effects and numerous uncontrolled environmental factors in the field (Guo et al. 2018a).

QTL mapping has been widely employed as an influential statistical approach to detect genomic regions related to important traits for breeding (Wang et al. 2019). Numerous QTL-based studies have been conducted on different populations for several DT-associated traits and GY components such as water use efficiency (Zhou et al. 2013), carbon isotope discrimination (Takai et al. 2006), canopy temperature (Prince et al. 2015), flag leaf size (Yue et al. 2008), heading date and delay in flowering (Trijatmiko et al. 2014), drought response index (Kim et al. 2017), leaf drying (Michael Gomez et al. 2010), grain number per panicle (Baisakh et al. 2020), biomass yield (Dixit et al. 2015) and plant height, number of tillers per plant, leaf rolling, leaf drying, harvest index, spikelet fertility, and relative water content (Barik et al. 2019).

Different positions of a QTL in various mapping populations result in a immense confidence interval and an unreliable QTL position. Additionally, several factors such as differences in mapping population size, sampling errors, marker density, experimental replicates and QTL mapping models can further complicate the situation (Darvasi and Soller 1997; Darvasi et al. 1993). Different methods have been applied till now to validate the QTL results, like QTL mapping utilizing first-generation populations, and confirmed in advanced-generation breeding populations of the same cross (Gelli et al. 2017). Furthermore, QTL validation is accomplished using the candidate gene method or positional cloning, followed by incorporating functional and genetic data within the breeding process (de Dorlodot et al. 2007). However, this is a challenging process that requires high-density linkage maps, extensive genomic resources and logical informatics data (de Dorlodot et al. 2007).

Meta-analysis is a statistical method that combines consensus loci from various QTL studies for multiple traitsinto a single dataset to determine the most probable position and confidence interval (CI) of QTL regions (Loni et al. 2023; Bilgrami et al. 2023). This approach has been applied to identify genomic consensus regions over various QTL studies considering their effects and constancy across different genetic backgrounds and environments. Moreover, it can enhanceand validate QTL positions on a consensus map through mathematical models. The identified consistent QTL for a set of QTLs with a CI of 95% via meta-analysis is called meta-QTL (MQTL) (Swamy et al. 2011). Another important advantage of meta-analysis of QTLs is its ability to decrease the CI of the MQTLs compared to QTLs. MQTL analysis determines the most constant QTLs irrespective of the population’s genetic background and field trial conditions, and it efficiently decreases the CI for identifying candidate genes and developing markers (Bilgrami et al. 2023; Khahani et al. 2021). Therefore, QTL-based meta-analysis provides more precise and stronger results. In addition, meta-analysis of QTLs provides a perception into the genetic analysis of complicated traits like drought, salt and heat response. Meta-analysis of QTLs has been applied to accurately evaluate many agronomical traits in different crops (Khowaja et al. 2009).

Several studies have been conducted for the meta-analysis of QTLs controlling grain yield components and DT-related traits under water deficit conditions in rice. Some MQTLs have been reported for plant height througha meta-analysis of published QTLs up to2009 (Khowaja et al. 2009). Swamy et al. (2011) projected 53 grain yield QTLs, reported in 15 studies under drought stress, on a consensus map and conducted a meta-analysis that resulted in the identification of 14 MQTLs on 7 chromosomes. They showed that a grain yield MQTL under drought coincided with at least one of the MQTLs discovered for root and leaf morphology in previous studies (Swamy et al. 2011). In another study, MQTL-analysis for grain yield and yield component under drought stress resulted in identification of a GY MQTL in a region close to the semi dwarf gene (*sd1*) locus on chromosome 1 which co-localized with QTLs for leaf rolling and osmotic adjustment (OA) (Trijatmiko et al. 2014). In addition, a QTL for percent seed set and grains per panicle under drought stress was discovered on chromosome 8 in the region where a QTL for OA was reported in previous studies (Trijatmiko et al. 2014). Yang et al. (2018a, b) reported some MQTLs for heading date through a meta-analysis of published QTLs till 2018 (Yang et al. 2018a). Khahani et al. (2021) conducted a meta-analysis of 536 QTLs related to yield and yield-associated traits such as yield (YLD), grain weight (GW), heading date (HD), plant height (PH) and tiller number (TN) plus root-architecture related traits under drought stress conditions. They identified 61 stable MQTLs across different genetic backgrounds and environments (Bilgrami et al. 2023; Khahani et al. 2021). Abdirad et al. (2022) combined root tip transcriptome sequencing and meta-analysis of QTLs to find the main genes engaged in drought stress response in rice (Abdirad et al. 2022). In addition, for complex quantitative traits, GWAS is widely employed to identify significant effects of genomic loci. It has been indicated that combining meta-analysis of QTLs and GWAS data can lead to dissecting important genomic regions and the genetic foundation of important quantitative traits (Bilgrami et al. 2023; Bilgrami et al. 2020; Daryani et al. 2022). Furthermore, an integrated approach of meta-QTL analysis was used to identify the genomic regions and candidate genes related to drought tolerance and yield-related traits in foxtail millet (Loni et al. 2023).

In the current study, a meta-analysis of 1,087 QTLs controlling DT and yield-related traits under drought stress conditions in rice was performed. The QTLs were gathered from QTL mapping studies conducted under drought stress in rice from 2000 to 2021. After mapping the 1,087 QTLs onto the consensus genetic map, the density of QTLs, described as the “QTL-overview index”, was computed for the considered interval of 0.5 cM on each chromosome to detect genomic regions significantly associated with yield and yield-related traits under drought stress conditions. Overlap between MQTLs identified using meta-analysis and SNPs identified using the GWAS technique for yield and yield-related traits under drought stress conditions was examined to select candidate genes. In our study, in addition to conducting a distinct MQTL analysis for each trait, an inclusive MQTL analysis was performed on all the investigated traits to identify and introduce hotspots for breeding programs. The consensus genomic regions identified by meta-analysis of QTLs were subsequently confirmed by the GWAS studies. The genes located within the MQTLs were found and categorized based on their function. Furthermore, the rice drought-responsive genes were identified by the RNA-seq and microarray datasets analysis, and the MQTL regions related to yield and yield-related traits were searched to identify the drought-responsive genes. Conclusively, the integration of QTLs, GWAS, and transcriptome data has facilitated the detection of the promising MQTLs and candidate genes. These findings would be utilized in MQTL-assisted breeding to improve yield potential under drought stress in rice.

## Materials and Methods

### Compilation of QTLs Linked to Drought Tolerance and Yield Associated Traits

All the publications reporting QTLs associated with DT and yield-related traits under drought treatment in rice from 2000 to 2021 were reviewed. Totally, 1,388 original QTLs belonging to 21 different traits from 134 bi-parental rice populations extracted from 76 studies, of which 1,087 QTLs were utilized for the meta-analysis. Out of this dataset, 1,087 QTLs (Table 1) were considered for the meta-analysis, which had the necessary information such as phenotypic variance, population size, etc. Moreover, the QTLs with a large confidence interval and small phenotypic variance were removed. Table 1 provides information on the parents used in the populations, the type and size of the populations, the markers used for genotyping (including AFLP, SSR, SNP, and RFLP), and the number of primary QTLs. The original QTLs were classified into 10 trait categories, including biomass yield (BY), canopy temperature (CT), drought response index (DRI), flag leaf size (FLZ), grain yield (GY), heading/days to flowering (HD), harvest index (HI), plant height (PH), panicle number (PN), and spikelet fertility (SF) (Additional file 4: Table S1).

**Table 1:** Brief of the QTL mapping studies used in meta-analysis of the QTLs for yield components and drought tolerance-associated traits in rice

No	Parents of population	Population size	Genotyping method	Population type	Number of initial QTL under drought stress	References
1	M23 × TC189	100	SSR	F_2_	26	Lin et al. (2007)
2	Basmati × IR55419-04	418	SSR	F_2_	12	Sabar et al. (2019)
3	CT9993 × IR62266	154	SSR, AFLP, RFLP	DH	78	Lanceras et al. (2004)
4	IR64 × Apo	50	SSR	BILs	3	Baghyalakshmi et al. (2016)
5	Zhenshan 97 × IRAT109	180	SSR	RILs	47	Yue et al. (2006)
6	N22 × Swarna, N22 × IR64, N22 × MTU1010	292, 289, 362	SSR	BSA	22	Vikram et al. (2011)
7	CR 143-2-2xKrishnahamsa	190	SSR	RILs	12	Barik et al. (2019)
8	VandanaxWay Rarem	126	SSR	F_3_	39	Bernier et al. (2007)
9	XiaobaijingzixKongyu131	220	SSR	RILs (F_2:7_)	13	Xing et al. (2014)
10	Swarna × WAB 450	188	SSR	BILs (BC_1_F_6_)	10	Sangodele et al. (2014)
11	Akihikari × IRAT109	106	SSR	BILs (BC_1_F_1_)	5	Kato et al. (2008)
12	Cocodrie × Vandana	187	SNP, SSR	F_2:3_	6	Solis et al. (2018)
13	Moroberekan × Swarna	260	SNP	BC_2_F_3_	47	Dixit et al. (2015)
14	Anbarbu × Spidroud	96	SSR	RILs	8	Sabouri et al. (2013)
15	Kali Aus × IR64, Kali Aus × MTU1010	300	SSR	BSA	7	Palanog et al. (2014)
16	IR20 × Nootripathu	200	SSR	RILs	35	Prince et al. (2015)
17	Swarna × Dular, IR11N121 × Aus196	350	SNP	BC_1_F_3_	41	Yadav et al. (2019)
18	Swarna × WAB450-I-B-P-157-2-1	202	SSR	BIL(BC_1_F_6_)	28	Saikumar et al. (2014)
19	Zhenshan97B × IRAT109	105	SSR	NILs	4	Nie et al. (2015)
20	Danteshwari × Dagad deshi	162	SSR, HvSSR	RILs	27	Verma et al. (2014)
21	IR77298-5-6-B-18 × IR64, IR77298-5-6-B-18 × IR77298-5-6-B-11, IR77298-14-1-2 × IR64, IR77298-14-1-2-B-10 × IR64	487, 478, 457, 286, 485	SSR	BILs (BC_4_F_3_)	10	Swamy et al. (2013)
22	IR55419-04/2 × TDK1	365	SSR	BSA (BC_1_F_3:4_)	19	Dixit et al. (2014a)
23	Kali Aus/2 × MTU1010, KaliAus/2 × IR64	134, 109	SSR	BSA (BC_1_F_4_)	6	Sandhu et al. (2014)
24	Cocodrie × N22	181	SNP	RILs	21	Bhattarai and Subudhi (2018)
25	IR74371-46-1-1 × Sabitri	294	SSR	BILs	9	Mishra et al. (2013)
26	Apo × Swarna, Aday sel × IR64, Vandana × Way Rarem	490, 288, 180, 470	SSR	BC_1_F_4_, BC_4_F_3_, BC_2_F_3_, BC_3_F_3_	14	Dixit et al. (2012)
27	Cocodrie × N22	190	SSR, SNP	F_2:3_	8	Dixit et al. (2012)
28	Swarna × Moroberekan	361	SNP	BC_2_F_3_	19	Dixit et al. (2014b)
29	Samgang × Nagdong	218	SSR, STS	DH	4	Kim et al. (2017)
30	Zhenshan97B × IRAT109	195	SSR	RILs	18	Liu et al. (2005)
31	Haogelao × Shennong265	94	SSR	RILs	31	Gu et al. (2014)
32	IR64 × Cabacu	154	SNP	RILs	11	Trijatmiko et al. (2014)
33	IR64 × Adaysel, Swarna × Apo, Vandana × Way Rarem	230	SSR	BC_1_F_5_	3	Shamsudin et al. (2016)
34	IR64 × MTU1010	119	SSR	BAC	12	Swamy et al. (2017)
35	N22 × Swarna, N22 × IR64, N22 × MTU1010	292, 289, 362	SNP	RILs	12	Vikram et al. (2011)
36	Norungan × IR62266	232	SSR	RILs	79	Suji et al. (2012)
37	Dongxiang × DXCWR	159	SSR	RILs	17	Tian et al. (2006)
38	Guichao2 × (0. sativa L. ssp. Indica)	135	SSR	BC_4_F_4_	12	Shao-Xia et al. (2006)
39	IRAT109 × Zhenshan 97	180	SSR	RILs	47	Yue et al. (2008)
40	IRAT109 × Zhenshan 97	181	SSR	RILs	41	Yue et al. (2005)
41	CT9993 × IR62266	154	SSR, AFLP, RFLP	DH	6	Babu et al. (2003)
42	IR64 × RAM 40 and RAM 90	513	SSR, STS	BC_2_F_3_	19	Bimpong et al. (2011)
43	CT9993-5-10-1-M × IR62266-42-6-2	154	SSR, AFLP, RFLP	DH	6	Chakraborty and Zeng 2011)
44	IR64 ×Azucena	135	RFLP, RAPD, Isozyme	DH	31	Courtois et al. (2000)
45	Swarna × Dhagaddeshi, IR64 × Dhagaddeshi	269	SSR	RILs (F_3:5_)	7	Ghimire et al. (2012)
46	IR20 × Nootripathu	259	ISSR, RAPD, EST	RILs (F_8_)	19	Michael Gomez et al. (2010)
47	Shennong265 × Haogelao	94	SSR	BILs (BC_3_F_6_)	9	Gu et al. (2012)
48	IR64 × Azucena	135	RFLP	DH	3	Hemamalini et al. (2000)
49	Zhenshan 97B × IRAT110	195	SSR	RILs (F_10_)	9	Hu et al. (2009)
50	Bala × Azucena	176	SSR, AFLP,RFLP	RILs (F_6_)	4	Khowaja and Price (2008)
51	CT9993-5-10-1-M × IR62266-42-6-2	105	SSR, AFLP, RFLP	DH	3	Kumar et al. (2007)
52	Bala × Azucena	205	SSR, AFLP, RFLP	RILs	21	Lafitte et al. (2004)
53	CT9993-5-10-1-M × IR62266-42-6-2	154	SSR, AFLP, RFLP	DH	36	Lanceras et al. (2004)
54	OM1490 × WAB880-1-38-18-20-P1-HB	229	SSR	BILs (BC_2_F_2_)	10	Lang et al. (2013)
55	Zhenshan 97B × IRAT109	187	SSR	RILs (F_10_)	12	(Liu et al. 2008)
56	Gharib × Sepidroud	148	SSR	F_2:4_	8	Mardani et al. (2013)
57	Maybelle × Baiyeqiu	251	SSR	DH	8	Qun et al. (2011)
58	IR62266-42-6-2 × IR60080-46-A	150	RFLP, SSR, Candidate genes	BILs	6	Robin et al. (2003)
59	Pusa Basmati1460 × MASARB 25, HKR47 × MAS26	94	SSR	RILs (F_2:3_)	25	Sandhu et al. (2013)
60	IR64 × Kali, MTU1010 × Kali, Kali Aus/2 × MTU1010	300	SSR	BILs (BC_1_F_4_)	23	Sandhu et al. (2014)
61	CT9993-5-10-1-M × IR62266-42-6-2	135	SSR, AFLP, RFLP	DH	4	Sellamuthu et al. (2015)
62	CT9993-5-10-1-M × IR62266-42-6-2	154	SSR, AFLP, RFLP	DH	4	Srinivasan et al. (2008)
63	IR64 × Norungan and IR50 × Norungan	380	SSR	RILs (F_6_)	28	Subashri et al. (2009)
64	Milyang 23 × Adhikari	126	SSR, RFLP	RILs	5	Takai et al. (2006)
65	IR64 × Azucena	91	SSR	DH	42	This et al. (2010)
66	IR64 × Azucena	165	SSR	RILs	32	This et al. (2010)
67	IR64 × IRAT177	154	SNP	RILs (F_6_)	11	Trijatmiko et al. (2014)
68	IR64 × Azucena	90	SSR	DH	4	Venuprasad et al. (2009)
69	Swarna × Basmati 334	367	SSR	RILs (F_3:4_)	8	Vikram et al. (2012)
70	MTU 1010 × N22	362	SSR	RILs (F_3:4_)	2	Vikram et al. (2011)
71	IR 64 × Tarom molaei	72	SSR	BILs ( BC_2_F_6_)	29	Wang et al. (2013)
72	Teqing × Lemont	133	SSR	BILs	33	Xu et al. (2005)
73	Sabitri × IR77298-5-6-18	294	SSR	BILs	4	Yadaw et al. (2013)
74	Tequing × Lemont	254	SSR	BILs	24	Zhao et al. (2008)
75	Zhenshan 97B × IRAT109	180	SSR	RILs (F_10_)	22	Zhou et al. (2011)
76	Zhenshan 97B × IRAT109	187	SSR	RILs (F_9_)	14	Zou et al. (2005)

### Consensus Genetic Map

The most comprehensive genetic map, developed by Wu et al., (2016) was used as a reference map for the meta-analysis of QTLs. This map integrated different types of markers such as SSR, RFLP and AFLP, from six rice saturated maps, and contained 6,970 markers spanning 1,823.1 cM with a genetic distance between markers ranging from 0.19 to 0.5 cM on all the chromosomes (Wu et al. 2016) (Additional file 5: Table S2). In order to incorporate those initial QTLs with SNP markers (Table 1) into the reference map, we employed our previous method (Daryani et al. 2022) in which the genomic positions of SNP markers on the rice genome were detected and the closest markers based on the physical position were utilized to project them on the reference map.

### Projection of QTLs into the Consensus Map

To project the QTLs on the reference map, the LOD (Logarithm of the odds) score, the phenotypic variation explained by the QTL (*R*^2^), the closest or flanking markers, and the position of the QTL-linked markers were used. A simple scaling rule was used to project the QTLs based on the consensus map, which involved the interval of the markers flanking the original QTL and the relevant interval on the chromosome. The projection was done using a Gaussian mixture model-based algorithm to estimate the new CI of a QTL on the consensus map. In the research, BioMercator v4.2 (http://moulon.inra.fr/) was used for meta-analysis of QTLs. The formula CI = 530/(N × *R*^2^) was used to calculate the 95% CI for QTLs obtained from backcross (BC) and F_2_ populations, where N is the population size and *R*^2^ is the proportion of phenotypic variance described by a QTL (Darvasi and Soller 1997). For QTLs obtained from doubled haploid (DH) and recombinant inbred (RI) lines, the formulas CI = 287/(N × *R*^2^) (Visscher and Goddard, 2004) and CI = 163/(N × *R*^2^) (Guo et al., 2006) were used to compute the 95% CI, respectively.

### Meta-QTL Analysis and QTL-Overview Index

Integrated QTLs on the consensus map were used to conduct MQTL analysis using BioMercator V4.2 (Arcade et al. 2004). Two types meta-analysis of QTLs were conducted; 1: Distinct MQTL analysis: individual trait-based analysis for 10 traits separately (Additional file 4: Table S1), 2: Inclusive MQTL analysis: a comprehensive analysis using all the original QTLs associated with drought tolerance and yield-related traits under drought stress. Two different methods were applied for MQTL analysis based on the number of primary QTLs. When the number of the primary QTLs was fewer than 10 for a chromosome, the method suggested by Goffinet and Gerber was used (Goffinet and Gerber 2000). Based on this approach, the model with the minimum AIC value was chosen for integrating QTLs and identifying MQTL positions. When the number of primary QTLs for an individual chromosome was at least 10, method recommended byby Veyrieras et al. was utilized (Veyrieras et al. 2007). This method used five criteria (AIC, AICc, AIC3, BIC, and AWE) for selecting the number of potential MQTLs on a chromosome. The model with the minimum value of three criteria out of five was selected as the best model, and the 95% CIs and MQTL positions were defined based on the chosen model. QTLs integration was done such that the peak position of the primary QTLs lay within the confidence interval of the MQTLs. QTLs with a membership probability greater than 60% for an MQTL were assigned to the same MQTL (Chardon et al. 2004). The “QTL-overview index” method was used to estimate the probability of QTL for 0.5-cM-long segment of the reference map (Daryani et al. 2022; Chardon et al. 2004).

### Identification of the Genes Located Within the MQTL Regions

The flanking markers were mapped onto the *Oryza sativa* Japonica group (IRGSP-1.0) reference genome (Kawahara et al. 2013) to determine their physical position. Finally, BioMart tool on the Ensemblplants website (https://plants.ensembl.org/biomart/martview/) was employed to find the genes placed within the MQTL regions (Additional file 6: Table S3 and Additional file 7: S4).

### Dataset Collection and Gene Expression Analysis

Differentially expressed genes (DEGs) under drought stress in rice were obtained from related microarray (11 published articles) and RNA-seq (13 published articles) data (Additional file 8: Table S5) available at https://www.ncbi.nlm.nih.gov. The genes having − 1 ≥ log_2_ fold change ≥ 1 and *p*-value ≤ 0.05 were identified as DEGs. The drought responsive genes placed within the MQTL regions were identified using Venn diagram (Venn diagram was drawn using a tool on this website: http://www.interactivenn.net/ (Heberle et al. 2015)) (Additional file 9: Table S6, Additional file 1: Fig. S1).

### Comparison of the MQTLs with DT-Associated Genome-Wide Association Studies (GWAS)

Reviewing DT-related GWAS studies (Bhandari et al. 2020; Courtois et al. 2013; Guo et al. 2018b; Kadam et al. 2018; Liang et al. 2016; Ma et al. 2016a; Pantaliao et al. 2016; Pariasca-Tanaka et al. 2020; Sandhu et al. 2019; Su et al. 2021; To et al. 2019; Zhang et al. 2021) was done to collect the reported SNP peak positions and discover the overlaps between their positions with MQTLs (Additional file 10: Table S7). Using the physical positions on the *Oryza sativa* Japonica group (IRGSP-1.0) reference genome, the genes placed within the SNP peak positions (± 25 kb) were identified.

### Graphical Representation

To visually represent the data, the Circos software (Krzywinski et al. 2009) were utilized to create a comprehensive graphical summary of the original QTLs, MQTLs, and QTL-overview statistics on all 12 rice chromosomes. Furthermore, additional graphs were generated using the ggplot2 *R* package.

## Results

### Collection of Original QTLs Associated with DT and Yield-Related Traits Under Drought Stress in Rice

A sum of 1,087 QTLs related to DT and yield-associated traits under drought stress in rice were collected which were from 134 different rice populations, including F_2_ (2 populations), BC (11 populations), RILs (30 populations), DH (13 populations), BAC (1 population), BIL (21 populations), BSA (4 populations), F_2:3_ (3 populations), F_3_ (1 population) and NILs (1 populations), with population size ranged from 50 (Baghyalakshmi et al. 2016) to 485 (Swamy BP et al. 2013) (Additional file 11: Table S8, Table 1). Additional file 11: Table S8 presents the complete information about the original QTLs including the QTL name, trait controlled by the QTL, related chromosome, LOD score, phenotypic variance described by the QTL, left and right flanking markers, interval between left and right markers (cM), parents of the mapping populations, type of the population, population size, number of used markers, location where the experiment was conducted and the reference.

**Fig. 1:**
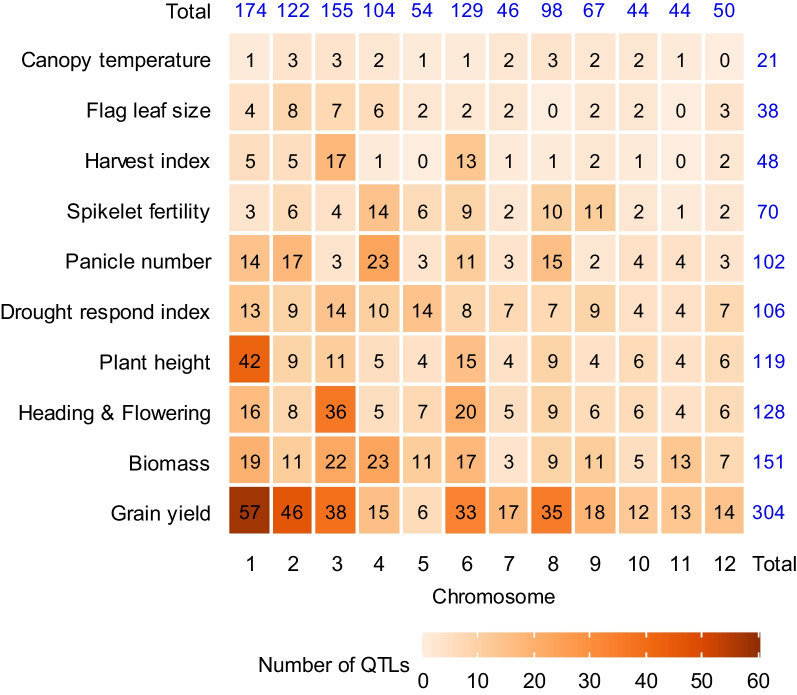
Dispersion pattern of the original QTLs related to yield components and drought tolerance associated traits under drought stress on the 12 rice chromosomes in terms of number

**Fig. 2:**
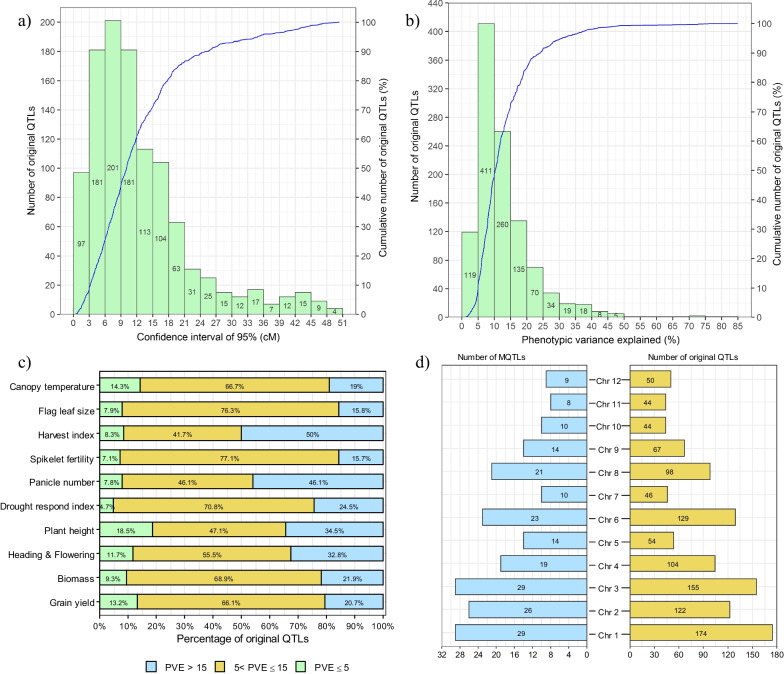
Brief of the 1,087 original QTLs associated with yield components and DT-associated traits utilized under drought stress for MQTL analysis. **a** The frequency dispersion of the original QTLs density according to diverse levels of the 95% confidence interval. **b** Original QTLs distribution based on the amount of the phenotypic variance explained by them, **c** The percentage of original QTLs with different phenotypic variance explained (*R*^2^ ≤ 5, 5 < *R*^2^ < 10 and *R*^2^ ≥ 15) for each of the yield components and DT-associated traits, **d** The dispersion of original QTLs and MQTLs on rice chromosomes. BY: biomass yield, CT: canopy temperature, DRI: drought response index, FLZ: flag leaf size, GY: grain yield, HD: heading/days to flowering, HI: harvest index, PH: plant height, PN: panicle number, SF: spikelet fertility

Analyzing the distribution pattern of these 1,087 QTLs across the rice chromosomes revealed interesting insights. chromosome 1 hosted the highest number of QTLs (176), followed by chromosomes 3 (155), 6 (128), 2 (122), 4 (104), 8 (98), 9 (67), 5 (53), 12 (50), 7 (46), 11 (44), and 10 (44), respectively (Fig. 1 and Table 2). The highest number of the initial QTLs related to grain yield (GY) (304), followed by biomass yield (BY) (151), heading/days to flowering (HD) (128), plant height (PH) (119), drought response index (DRI) (109), panicle number (PN) (102), spikelet fertility (SF) (71), harvest index (HI) (48), flag leaf size (FLZ) (38) and canopy temperature (CT) (21), respectively (Table 2). These QTLs exhibited 95% CI spanning from 0.43 to 50.40 cM, with an average of 12.36 cM. Notably, nearly 30% and 85% of the original QTLs had CIs of less than 10 cM and 20 cM, respectively (Fig. 2a). In terms of the phenotypic variance described by the investigated QTLs, there was considerable variability, ranging from 1.1% to 85% (Fig. 2b). Each trait's original QTLs were ranked based on the proportion of phenotypic variance they accounted for (Fig. 2c). Remarkably, 52.7% of the original QTLs (585 out of 1,087) described more than 10% of the phenotypic variance, while 47.2% of them (524 out of 1,087) had a PVE of less than 10% (Fig. 2b). Specifically, out of the 304 QTLs linked to GY, a total of 173 QTLs surpassed the 10% threshold for PVE (Fig. 2c).

**Table 2:** Distribution of the original QTLs used for the MQTL analysis on 12 rice chromosomes

Yield traits	Chromosome												Total QTLs	QTLs proportion (%)	MQTL number^b^
	1	2	3	4	5	6	7	8	9	10	11	12			
BY	20	11	22	23	11	17	3	9	11	5	12	7	151	13.61	29
CT	1	3	3	2	1	1	2	3	2	2	1	0	21	1.89	2
DRI	13	9	14	10	14	8	7	7	9	4	4	7	106	9.55	26
FLZ	4	8	7	6	2	2	2	0	2	2	0	3	38	3.42	10
GY	56	46	38	15	5	33	17	35	18	13	13	15	304	27.41	49
HD	16	8	36	5	7	20	5	9	6	6	4	6	128	11.54	27
HI	5	5	17	1	0	13	1	1	2	1	0	2	48	4.32	10
PH	43	9	11	5	4	15	4	9	4	6	4	5	119	10.73	26
PN	13	17	3	23	3	11	3	15	2	4	5	2	101	9.10	26
SF	5	6	4	14	6	8	2	10	11	1	1	3	71	6.40	13
Total QTLs	181	123	159	104	54	130	46	100	70	44	46	52	1087		
QTLs proportion (%)	16.32	11.90	14.33	9.37	4.86	11.72	4.14	9.01	6.31	3.96	4.14	4.68			
Total MQTLs^a^	31	28	28	19	14	23	10	22	14	10	8	11			
MQTLs proportion (%)	14.22	12.84	12.84	8.71	6.42	10.55	4.58	10.09	6.42	4.58	3.66	5.04			

a. Numbers in brackets indicate the total number of MQTLs identified on each chromosome

b. Number of MQTL containing an individual QTL for the trait

BY: biomass yield, CT: canopy temperature, DRI: drought respose index, FLZ: flag leaf size, GY: grain yield, HD: heading/days to flowering, HI: harvest index, PH: plant height, PN: panicle number, SF: spikelet fertility

### Identification of MQTLs Controlling Yield Components and DT-Related Traits by Meta-Analysis

**Fig. 3:**
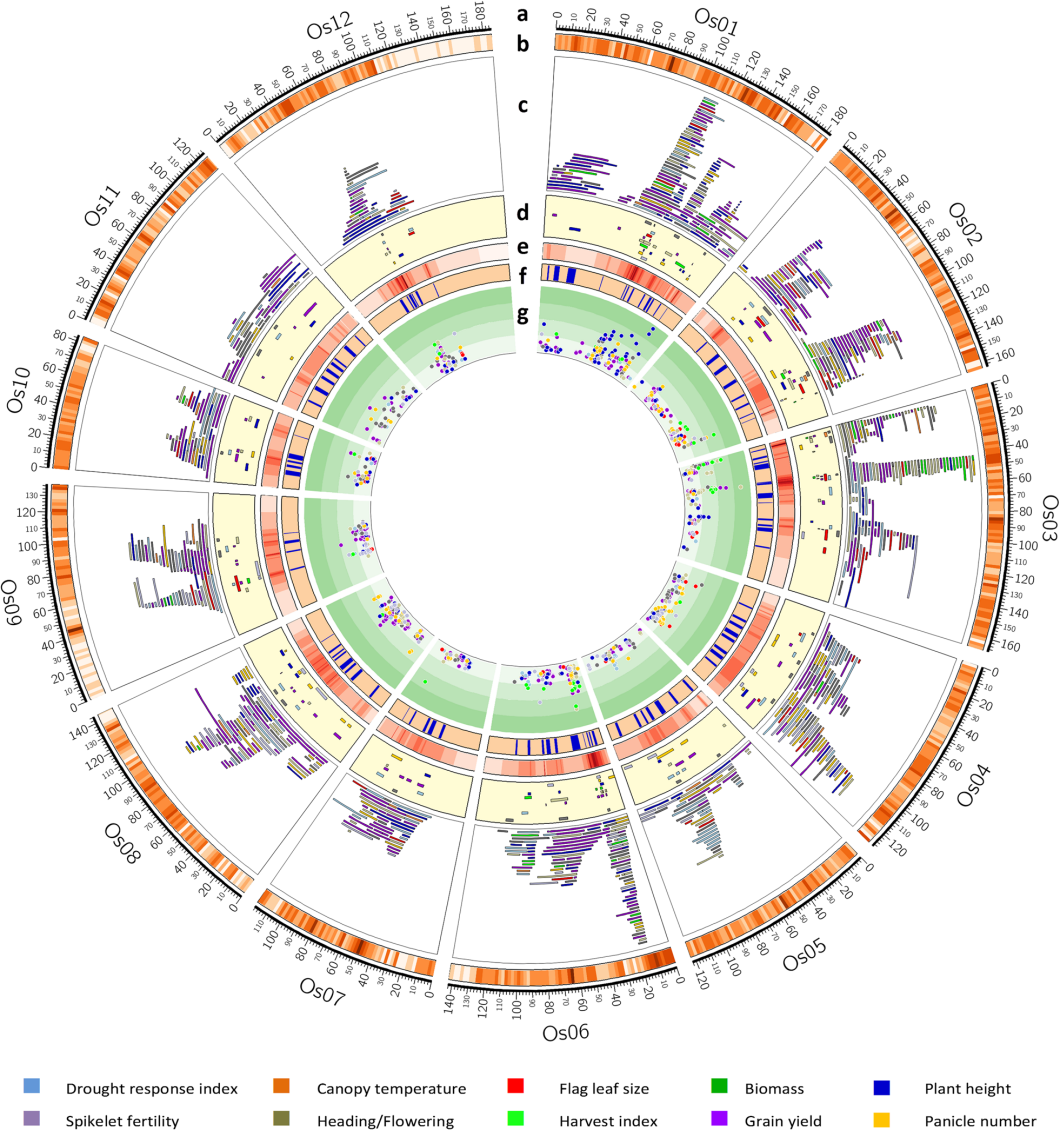
Concentric circles indicate different features drawn in Circos (Krzywinski et al. 2009). **a** Genetic positions (cM) of rice chromosomes represented by bars. **b** Molecular markers density on rice chromosomes shown on a scale from white to orange to indicate the lowest to highest density. **c** Distribution of QTLs across the twelve rice chromosomes. **d** Genetic positions of MQTLs for each distinct trait with 95% CIs. **e** Heatmap illustrating the QTL-overview index, which is estimated by the frequency of QTLs for yield components and DT-associated traits on each 0.5-cM segment of the rice consensus map. **f** Genetic positions of inclusive MQTLs with 95% CIs. **g** Proportion of phenotypic variance explained (*R*^2^) by each QTL

The collected original QTLs were categorized into 10 major traits and subjected to MQTL analysis to detect MQTLs controlling each trait associated with DT and yield components under drought stress in rice. The meta-analysis resulted in the identification of 213 MQTLs with at least eight MQTLs on each chromosome (Fig. 2d; Table 2 and 3). The identified MQTLs included 50 MQTLs for GY, 29 MQTLs for BY, 27 MQTLs for HD, 25 MQTLs for PN, 23 MQTLs for PH, 24 MQTLs for DRI, 13 MQTLs for SF, 10 MQTLs for FLZ and HI and two MQTLs for CT. MQTL_GY1.4 was recognized as the most stable MQTL with the greatest number of original QTLs (22) (Fig. 3; Table 3). In addition, the results indicated that 29 MQTLs out of the detected 213 MQTLs were involved in the genetic control of more than one trait (Additional file 12: Table 9). These MQTLs were distributed across the rice chromosomes, with the number of MQTLs per chromosome varying, ranging from 29 MQTLs on chromosome 1 to eight MQTLs on chromosome 11 (Fig. 2d; Tables 2 and 3). The results indicated that all the chromosomes of rice were engaged in controlling yield components and DT-related traits. The number of initial QTLs grouped in a MQTL varied from two to 22 QTLs (Table 3). The proportion of phenotypic variance explained by these MQTLs displayed a range from 3.05% to 70.1%, with an average of 12.76% (Table 3). When it comes to the 95% CIs for these identified MQTLs, they spanned from a minimal 0.12 cM for the RM482-RM213 interval on chromosome 2 to a maximal 19.7 cM for the E2801S-HSP70A interval on chromosome 5 (Table 3).Notably, a narrower confidence interval was observed for each MQTL when compared to the average CI of the original QTLs clustered within that specific MQTL. Specifically, the confidence interval of 17 MQTLs was diminished to less than 1 cM, effectively reducing the length of these MQTLs by approximately 15.66 times when compared to the average CI of the original QTLs. The mean phenotypic variance for these 17 MQTL regions was calculated at 20.3. It's worth noting that the flanking markers of these aforementioned MQTLs present as promising candidates for deploying molecular breeding and marker-assisted selection strategies aimed at bolstering drought tolerance in rice (Table 3).

**Table 3:** Summary of the identified trait specific MQTLs. The initial QTLs for yield components and drought tolerance associated traits under drought stress in rice were categorized into ten major traits* and subjected to meta-QTL analysis independently

MQTL	Chr.	Position	CI (95%)	Mean CI of initial QTL	Mean variance of initial QTL (*R*^2^)	Initial QTL number	No. of study	Left marker	Right marker	Left position	Right position	Number of genes
MQTL_GY1.1	1	9.34	4.17	23.5	7.81	8	8	RM3148	RM1141	746,170	1,622,487.5	140
MQTL_PH1.1	1	31.70	11.27	24.35	8.56	5	5	R1944	RM5359	4,545,326	7,178,518.5	326
MQTL_GY1.2	1	75.26	2.48	6.49	10.58	4	4	RM600	G1184A	9,463,704.5	13,565,767	359
MQTL_BY1.1	1	100.05	6.04	18.82	13.16	6	6	RZ776	OSR23	23,118,504	24,263,243	136
MQTL_HD1.1	1	101.69	6.22	7.17	10.45	6	6	M163A	G302	23,546,970	24,908,092	191
MQTL_GY1.3	1	101.83	1.84	19.07	9.50	4	4	L588	RM104	23,852,251	25,067,017	165
MQTL_HI1.1	1	104.93	8.02	13.35	5.66	2	2	L588	RM3475	23,852,251	26,041,116	270
MQTL_PN1.1	1	104.96	4.38	14.21	17.49	6	6	RM104	C949A	24,188,607	25,067,017	125
MQTL_FLZ1.1	1	105.81	5.35	12.07	8.55	4	4	GA273	E26M47.361-P1	24,188,607	27,394,545	402
MQTL_BY1.2	1	106.46	0.73	4.39	24.38	5	5	RG345	C949A	25,067,017	25,637,999	64
MQTL_HD1.2	1	106.65	6.97	10.51	5.53	3	3	G3001	Est15	24,735,562	25,975,208	152
MQTL_PH1.2	1	106.74	1.08	7.73	18.19	10	10	RM3642	C949A	24,865,235	25,067,017	131
MQTL_DRI1.1	1	107.91	3.86	13.61	11.30	10	10	RG233	ME10_14	25,067,017	25,975,208	142
MQTL_GY1.4	1	108.43	0.88	8.87	12.46	22	22	C808	RM3475	25,637,999	26,041,116	44
MQTL_SF1.1	1	110.52	6.17	12.27	10.01	3	3	C949A	C49	25,067,017	26,884,639	210
MQTL_PH1.3	1	114.73	1.94	5.44	28.33	6	6	RG345	C49	25,067,017	26,884,639	211
MQTL_HD1.3	1	115.37	3.02	6.59	10.49	2	2	C49	RG957	26,884,639	27,147,779	32
MQTL_PH1.4	1	129.85	1.46	5.57	21.54	11	11	RM1183	RM3411	30,979,208	31,311,412	48
MQTL_BY1.3	1	129.94	4.95	10.07	9.90	3	3	RZ730	RM6666	29,895,513	31,706,943.5	302
MQTL_PN1.2	1	130.63	4.17	7.52	22.34	3	3	E21042S	Y4564L	30,820,894	31,712,003	135
MQTL_HD1.4	1	131.40	6.86	10.47	5.21	6	6	G1372	R3347	30,498,428	32,455,343.5	306
MQTL_GY1.5	1	139.11	1.71	10.59	12.86	9	9	Y6185LB	C2340	33,477,084	34,473,168	149
MQTL_HI1.2	1	147.16	10.10	14.58	6.0	2	2	RM6608	RM8235	34,689,018	38,438,231	560
MQTL_PH1.5	1	150.04	1.44	4.56	29.33	2	2	RM5382	RM6696	38,053,956	38,220,799	122
MQTL_PH1.6	1	153.61	1.86	13.36	19.77	2	2	RM8235	RM5759	38,438,231	39,019,522.5	94
MQTL_GY1.6	1	157.09	0.17	12.71	11.31	9	9	RM3561	RM6827	39,079,856	39,202,876	17
MQTL_BY1.4	1	161.40	1.73	5.06	18.15	4	4	G7003	S10304	39,949,325	40,362,513	55
MQTL_PH1.7	1	161.40	0.57	1.0	70.10	3	3	T91	S10304	40,142,055	40,362,513	28
MQTL_PN1.3	1	161.40	2.11	3.93	18.66	3	3	G7003	B183	39,949,325	40,327,076	52
MQTL_PN2.1	2	31.46	3.46	9.54	15.53	4	4	RG509	RM259	5,477,602	7,260,366	234
MQTL_GY2.1	2	32.41	1.29	9.55	11.50	14	14	RM211	RZ87	6,586,865	6,786,804	33
MQTL_DRI2.1	2	33.85	5.55	7.89	11.51	2	2	RM2483	RM3828	6,169,421	7,646,650	175
MQTL_PH2.1	2	40.36	5.47	11.91	11.88	4	4	V5A	ME10_18	8,265,516	9,416,543	105
MQTL_BY2.1	2	48.31	10.12	18.14	5.56	3	3	RM174	RG171	7,006,218	17,484,902	776
MQTL_FLZ2.1	2	52.34	11.83	16.72	5.66	2	2	E13M59.M003-P1	RM6844	9,563,380	18,063,821	578
MQTL_GY2.2	2	52.39	8.75	24.33	5.60	4	4	RM3501	R1736	10,187,595	16,441,264	416
MQTL_DRI2.2	2	53.51	9.97	18.16	6.59	3	3	RM3501	RM6844	10,187,595	18,063,821	526
MQTL_PN2.2	2	60.35	5.74	10.37	8.6	3	3	RM7426	Sbe3	16,672,602	20,795,042	352
MQTL_GY2.3	2	67.87	11.82	19.09	11.88	2	2	RM4499	G1314A	18,529,899	21,006,985	151
MQTL_PN2.3	2	104.24	8.76	12.35	8.0	2	2	RM6617	RM526	24,761,070	26,665,000	254
MQTL_HD2.1	2	107.32	3.45	9.18	6.82	4	4	EMP2_8	RM5706	26,084,783	26,479,677	55
MQTL_GY2.4	2	109.87	2.43	10.21	6.09	13	13	RM526	RM5470	26,665,001	27,148,197	70
MQTL_HI2.1	2	110.52	5.93	11.26	6.79	3	3	RM7245	L107	26,443,082	27,598,462	156
MQTL_BY2.2	2	119.65	4.45	12.01	8.49	5	5	RG102	CDO686	27,483,174	30,916,360	469
MQTL_PH2.2	2	119.67	7.93	23.12	12.53	2	2	RM573	RM318	27,940,926	29,631,619	225
MQTL_GY2.5	2	120.16	2.79	11.16	6.69	6	6	RM1342	CDO686	28,159,577	28,947,586	400
MQTL_HD2.2	2	121.11	11.65	11.27	7.65	2	2	RM221	C92	27,609,970	29,818,949	284
MQTL_DRI2.3	2	121.57	3.90	7.00	10.67	3	3	E13M60.258-P1	RM318	28,415,795	29,631,619	171
MQTL_CT2.1	2	122.33	5.13	8.34	8.86	2	2	E13M60.258-P1	Amy1	28,415,795	29,028,110	93
MQTL_FLZ2.2	2	130.08	9.70	17.3	9.57	3	3	RM497	RG256	29,028,110	33,938,928	772
MQTL_PN2.4	2	133.04	1.21	11.30	12.33	6	6	E26M47.586-P2	RM6519	31,262,234	33,828,029	371
MQTL_SF2.1	2	133.04	1.17	17.68	14.77	2	2	E26M47.586-P2	RM6519	31,262,234	33,828,029	371
MQTL_GY2.6	2	139.29	0.12	7.02	14.94	4	4	RM482	RM213	32,486,713	34,652,409	325
MQTL_BY2.3	2	139.64	0.93	10.80	17.56	3	3	RM482	RM208	32,486,713	35,135,925	403
MQTL_HI2.2	2	139.68	0.75	2.46	25.5	2	2	C560	RM208	34,652,409	35,135,925	79
MQTL_BY3.1	3	4.47	1.98	4.19	20.0	4	4	V10A	S12021	797,086	1,269,836	78
MQTL_HI3.1	3	4.72	1.41	5.98	22.44	5	5	RM3894	S12021	1,117,031	1,269,836	17
MQTL_HD3.1	3	4.74	0.95	6.60	28.16	7	7	RM3894	S12021	1,117,031	1,269,836	17
MQTL_GY3.1	3	4.80	1.31	7.92	21.42	7	7	RM3894	S12021	1,117,031	1,269,836	17
MQTL_CT3.1	3	6.08	4.36	12.07	8.48	3	3	RM3894	RM4266	1,117,031	1,730,466	93
MQTL_HD3.2	3	8.24	4.20	14.04	9.92	2	2	RM4853	C1279	1,396,256	2,143,176	150
MQTL_BY3.2	3	11.94	6.27	17.13	4.37	5	5	RM4266	d14	1,730,466	3,285,827	269
MQTL_PN3.1	3	12.89	4.79	6.91	9.55	2	2	C1279	RM6849	2,143,176	3,285,827	187
MQTL_GY3.2	3	14.94	2.11	3.02	15.0	4	4	RM6301	RM2421	2,651,426	3,453,460	116
MQTL_HD3.3	3	20.0	0.84	1.46	31.0	3	3	B224	V166	4,096,077	4,481,736	85
MQTL_HD3.4	3	44.8	1.59	8.39	27.95	2	2	S1524	RM489	9,312,909	10,141,494	176
MQTL_BY3.3	3	45.68	1.40	9.34	13.15	7	7	G175	RM218	5,181,531	8,405,561	440
MQTL_GY3.3	3	45.72	0.63	10.49	14.97	12	12	RM545	RM218	4,948,027	8,405,562	476
MQTL_HI3.2	3	46.10	2.02	9.06	20.88	12	12	RG100	E25M59.156-P1	9,439,157	10,444,810	171
MQTL_FLZ3.1	3	47.20	6.65	9.93	9.46	2	2	S1524	RM282	9,312,909	12,407,446	448
MQTL_HD3.5	3	47.61	0.34	11.68	21.18	18	18	S804	RG369	10,444,810	10,464,780	34
MQTL_DRI3.1	3	47.84	4.04	14.62	10.62	4	4	RM389	RZ574	9,888,598	10,600,298	117
MQTL_GY3.4	3	48.63	1.53	8.50	13.97	17	4	RG369	RG369A	8,964,666	11,308,981	364
MQTL_DRI3.2	3	60.97	7.67	25.75	8.85	4	4	RM5928	RZ399	12,574,977	14,291,272	173
MQTL_HD3.6	3	69.75	2.56	5.13	9.66	3	3	E4M19_3	C11260S	14,860,539	15,471,989	55
MQTL_GY3.5	3	71.90	3.96	25.43	9.98	2	2	RM55	RM411	15,335,803	15,646,621	32
MQTL_PH3.1	3	91.49	1.88	7.24	17.20	5	5	C2394	RG96B	23,048,637	23,825,893	65
MQTL_DRI3.3	3	96.05	3.5	13.15	13.94	5	5	RZ745	G200	23,088,637	24,595,174	116
MQTL_HD3.7	3	98.49	0.60	22.06	9.88	2	2	G200	S10742	24,595,174	24,848,573	30
MQTL_GY3.6	3	98.93	0.34	17.97	8.42	8	8	RM293	C50029S	24,848,574	24,994,874	10
MQTL_BY3.4	3	102.02	5.69	14.04	9.94	5	5	RM5626	E24M50 159-P2	24,864,440	25,853,668	104
MQTL_FLZ3.2	3	104.01	10.49	15.0	6.08	2	2	RM293	RM571	24,848,574	25,128,711	31
MQTL_SF3.1	3	106.49	7.25	25.29	10.60	3	3	RZ261	RZ598	25,091,445	26,874,145	187
MQTL_FLZ3.3	3	119.49	5.84	16.52	11.0	3	3	RM3350	RM85	26,669,725	28,024,209	151
MQTL_BY4.1	4	22.75	2.11	10.47	13.86	4	4	E2466S	S1621	13,074,907	14,669,542	77
MQTL_FLZ4.1	4	28.22	2.75	4.33	20.34	2	2	E13M59.423-P2	E26M49.494-P2	13,154,408	13,634,944	20
MQTL_DRI4.1	4	29.87	8.64	15.66	10.80	3	3	RM3317	G318	13,645,141	16,774,391	117
MQTL_PN4.1	4	41.24	6.69	11.54	16.23	3	3	EMP3_1c	E61747S	16,966,299	19,053,076	133
MQTL_GY4.1	4	41.58	2.96	22.25	7.03	3	3	RM417	tsv1	18,391,172	18,674,598	28
MQTL_PN4.2	4	48.43	6.28	8.99	20.75	2	2	E61747S	CDO456	18,674,598	20,087,233	141
MQTL_DRI4.2	4	48.81	4.64	17.28	11.82	4	4	Y34L	RG788	18,674,598	20,087,232	141
MQTL_BY4.2	4	53.01	3.57	10.93	8.61	7	7	RG788	RM564	19,259,874	19,948,178	69
MQTL_GY4.2	4	63.88	5.44	20.70	8.60	8	8	ME10_11	C558	21,101,000	22,041,773	111
MQTL_BY4.3	4	67.76	4.65	11.20	7.70	5	5	E60696S	RM2439	21,817,873	22,912,474	176
MQTL_SF4.1	4	85.50	4.61	11.94	12.0	2	2	Gm6	v15	26,793,973	27,865,967	142
MQTL_PN4.3	4	86.09	4.21	10.63	13.01	5	5	C11378	V15	27,171,556	27,865,967	110
MQTL_BY4.4	4	97.30	4.68	15.46	9.16	7	7	RM348	RM3474	28,907,417	29,709,214.5	108
MQTL_SF4.2	4	103.75	4.21	15.89	10.78	10	10	R2017	cul5	30,189,857	30,853,328	98
MQTL_DRI4.3	4	103.84	9.0	22.0	9.26	3	3	RM3474	RM3843	29,709,215	31,498,619.5	284
MQTL_FLZ4.2	4	105.18	2.84	8.97	20.34	3	3	M255	G379B	30,772,481	31,478,421	121
MQTL_PN4.4	4	106.37	3.33	11.66	15.97	11	11	RZ905	RM3836	30,853,328	31,626,440.5	127
MQTL_PH4.1	4	108.62	8.8	15.80	8.90	3	3	V65	RM5950	30,853,328	32,846,064	337
MQTL_GY4.3	4	112.70	12.54	33.96	7.66	3	3	V125	S15892	31,478,421	33,595,720	323
MQTL_HD5.1	5	7.34	2.51	3.57	10.50	2	2	RM4777	V82	453,378	948,313	82
MQTL_BY5.1	5	30.48	2.99	6.25	12.75	4	4	S12447	RM3322	3,500,387	4,262,806	83
MQTL_PN5.1	5	50.64	8.31	26.82	15.07	2	2	RM5994	RM4959	6,883,642	13,008,173	281
MQTL_GY5.1	5	51.29	7.53	11.06	9.50	2	2	E11511S	RG776B	7,271,761	13,008,173	242
MQTL_DRI5.1	5	65.84	3.64	5.55	14.12	2	2	E2801S	RM1237	16,960,667	17,956,138	106
MQTL_GY5.2	5	66.36	8.84	15.65	7.50	2	2	RM465C	RM163	16,751,228	19,189,515	247
MQTL_DRI5.2	5	72.71	3.17	10.76	19.33	6	6	RM6628	RM8211	18,911,700	19,005,255	10
MQTL_PN5.2	5	74.22	19.66	32.42	11.61	2	2	E2801S	HSP70A	16,960,667	22,671,315	644
MQTL_HD5.2	5	75.31	3.50	14.32	11.15	4	4	V163	RM173	18,875,245	21,644,524	324
MQTL_BY5.2	5	75.87	4.66	18.71	12.76	5	5	V163	RZ70	18,875,245	20,420,897	169
MQTL_DRI5.3	5	80.29	6.13	19.02	8.54	5	5	RM173	HSP70A	21,644,525	22,671,315	125
MQTL_PH5.1	5	83.16	3.01	7.46	9.90	2	2	S10569	C308	20,810,731	21,064,677	30
MQTL_SF5.1	5	87.8	10.21	15.95	5.90	2	2	BCD454	RM334	19,656,235	26,848,229	955
MQTL_SF5.2	5	115.66	8.61	15.53	12.33	3	3	RM7653	S11036	27,359,989	29,077,289	289
MQTL_PN6.1	6	3.20	6.40	11.64	9.94	2	2	V83	hst1	485,985	1,675,988	174
MQTL_HD6.1	6	12.88	1.49	8.00	11.68	4	4	G89-2B	Amp5	3,081,988	3,920,872	152
MQTL_GY6.1	6	13.53	1.21	4.08	19.03	3	3	RM8200	AID1	3,197,784	4,016,645	153
MQTL_PH6.1	6	14.99	3.56	9.90	10.08	5	5	B67	WC	3,168,238	3,459,621	50
MQTL_BY6.1	6	15.16	2.33	9.80	8.81	11	11	S1434	RM204	3,920,871	4,886,363	136
MQTL_HD6.2	6	16.41	1.44	3.78	20.77	7	11	L533	RM204	4,224,235	4,288,983	117
MQTL_PN6.2	6	16.65	1.94	5.37	12.87	7	7	L533	RM197	4,224,235	4,886,363	92
MQTL_SF6.1	6	16.73	3.78	9.96	7.15	6	6	AID1	L1092	4,016,645	4,886,362	115
MQTL_HI6.1	6	16.75	2.06	5.86	13.33	3	3	RM4608	RM197	3,818,593	4,886,363	152
MQTL_HD6.3	6	17.24	1.72	4.0	17.65	2	2	RM204	R1966	4,346,386	4,886,363	266
MQTL_GY6.2	6	17.76	2.92	9.33	8.14	11	11	RM204	L1092	4,346,386	4,886,362	259
MQTL_GY6.3	6	21.82	1.90	6.81	18.87	4	4	L1092	RM253	4,886,362	5,425,505	82
MQTL_HI6.2	6	21.97	1.65	2.93	32.55	2	2	OsScS1	RM111	4,886,362	5,096,805	50
MQTL_GY6.4	6	55.10	1.97	25.87	8.93	10	10	RM6836	GA21	9,309,026	9,537,499	16
MQTL_BY6.2	6	59.19	8.29	16.9	7.90	2	2	Y2145L	R2123	9,537,499	11,680,746	143
MQTL_PH6.2	6	60.14	1.88	16.04	14.77	4	4	RM5427	RG648	9,862,362	19,337,857	567
MQTL_HD6.4	6	61.21	17.09	28.35	8.17	3	3	RM173	R2654	8,745,047	24,454,963	1129
MQTL_SF6.2	6	68.78	6.19	10.95	8.15	2	2	EMP3_6	CDO17B	13,842,895	20,402,199	361
MQTL_GY6.5	6	74.9	0.27	12.21	10.91	4	4	EM17_4	Hwg1	21,164,176	21,731,459	48
MQTL_PH6.3	6	91.41	3.10	6.67	6.80	4	4	W475A	DI2	24,521,612	25,314,832	74
MQTL_HI6.3	6	92.73	6.95	14.95	7.11	3	3	TP1D2D7D	RM5371	24,188,952	25,825,476.5	167
MQTL_DRI6.1	6	95.90	6.14	15.69	11.13	5	5	P12	RM3567	24,937,632	25,996,208.5	103
MQTL_BY6.3	6	110.40	7.14	17.87	11.33	3	3	RG244	RM5183	27,377,223	28,679,518	181
MQTL_PH7.1	7	47.0	5.99	24.58	6.29	4	4	Rc	RM2878	6,065,104	14,636,863	478
MQTL_DRI7.1	7	47.52	5.67	19.04	8.86	4	4	RM1253	RM214	6,967,952	12,783,566	326
MQTL_GY7.1	7	50.18	6.27	10.95	7.87	3	3	L538T3	RM2530	4,086,224	15,569,337	749
MQTL_GY7.2	7	62.75	4.43	13.86	7.10	4	4	cul7	V209	17,526,630	19,357,439	221
MQTL_GY7.3	7	70.89	4.37	13.22	7.22	5	5	RM11	RM3826	19,256,977	20,807,448	155
MQTL_BY7.1	7	71.07	4.40	7.74	9.16	3	3	RM2966	RM3826	19,852,944	20,807,448	99
MQTL_PN7.1	7	84.23	11.56	16.35	11.40	2	2	RM560	EM11_1	19,583,162	25,949,623	813
MQTL_GY7.4	7	88.24	4.56	20.88	9.31	5	5	ME10_6	EM11_1	24,790,186	25,949,623	149
MQTL_HD7.1	7	89.14	1.88	15.39	10.93	4	4	Ctb12	EM11_1	24,809,203	25,949,623	146
MQTL_DRI7.2	7	90.68	7	19.46	8.86	3	3	RM47	C50076S	24,809,203	25,414,203	77
MQTL_PN8.1	8	15.74	3.77	5.36	30.45	2	2	RM1019	T77	201,439	680,624	80
MQTL_GY8.1	8	18.34	8.11	17.53	8.33	3	3	T92	RZ597	151,275	1,719,825	236
MQTL_PN8.2	8	24.25	5.66	12.42	7.68	2	2	Sdr5	R2007	680,624	1,719,807	148
MQTL_GY8.2	8	48.55	3.89	23.03	10.06	5	5	RM3572	S1850A	3,927,397	4,999,050	107
MQTL_SF8.1	8	60.71	16.07	32.01	4.70	3	3	RZ562	RM483	5,423,216	15,659,785	617
MQTL_HD8.1	8	61.70	2.25	10.66	9.37	5	5	PI11	HSA2	7,552,919	8,669,778	100
MQTL_DRI8.1	8	66.0	4.96	17.31	8.42	4	4	S10588	C10983S	8,669,777	15,126,603	338
MQTL_GY8.3	8	67.18	2.55	8.94	13.90	7	7	RM3395	C1356	10,293,807	15,700,926	260
MQTL_PN8.3	8	74.44	5.35	17.89	8.73	2	2	S21441S	ME5_5	16,916,687	19,381,569	182
MQTL_BY8.1	8	78.02	6.73	15.70	10.12	3	3	RG1034	RM342A	16,371,454	19,953,061	279
MQTL_GY8.4	8	80.72	2.04	6.21	21.98	10	10	RM4815	G192	19,006,601	19,953,061	101
MQTL_GY8.5	8	84.94	1.45	11.10	11.80	11	11	RM33	RM350	20,094,614	20,650,152	58
MQTL_BY8.2	8	90.92	4.50	11.29	11.73	3	3	RM3459	RG418B	20,662,517	22,471,934	170
MQTL_DRI8.2	8	91.23	7.56	13.95	8.42	2	2	ME9_1	RM458	20,662,517	22,339,914	157
MQTL_SF8.2	8	91.49	6.45	16.77	8.18	6	6	RM284	RM447	21,142,459	26,547,047	639
MQTL_PH8.1	8	91.62	4.7	7.50	16.55	2	2	RM264	S11114	20,971,442	21,475,767	60
MQTL_PN8.4	8	92.99	3.85	8.55	16.17	4	4	RM256	RM458	21,218,824	22,339,914	314
MQTL_GY8.6	8	102.9	0.44	18.53	9.08	3	3	S11102	M235	22,876,619	23,115,032	30
MQTL_PH8.2	8	103.93	5.57	9.66	19.3	3	3	E23M50.101-P1	RZ70A	22,332,152	24,185,871	20
MQTL_PH8.3	8	119.77	6.31	8.91	20.90	2	2	S5064S	R3961S	25,263,639	26,644,393	20
MQTL_PN8.5	8	119.77	1.62	10.31	18.47	4	4	C10122S	RM1345	25,759,900	26,145,035	121
MQTL_HD9.1	9	39.06	4.0	16.70	8.52	3	3	G338	E13M60.130-P2	1,000,000	2,351,510	76
MQTL_GY9.1	9	47.44	2.92	6.32	16.61	3	3	RM6021	DP	5,307,742	6,387,633	71
MQTL_SF9.1	9	47.50	2.62	14.43	13.28	7	7	RM6021	DP	5,307,742	6,387,633	69
MQTL_FLZ9.1	9	47.69	15.91	26.48	3.82	2	2	MGD9	E13M59.099-P2	1,000,000	6,387,633	310
MQTL_HI9.1	9	47.69	5.09	7.29	12.51	2	2	RM316	RM219	3,762,781	6,387,633	163
MQTL_DRI9.1	9	47.92	2.42	6.97	16.88	4	4	RM8206	DP	5,919,594	6,387,633	29
MQTL_SF9.2	9	57.78	15.87	22.45	8.30	2	2	C50257S	RM105	6,021,201	14,372,099	527
MQTL_GY9.2	9	66.44	2.05	3.34	23.06	3	3	RG553	EM14_6	9,216,583	14,372,099	471
MQTL_GY9.3	9	70.13	1.68	11.31	10.50	6	6	RM409	ME4_13	14,372,099	15,547,337	140
MQTL_BY9.1	9	79.22	6.33	15.57	11.58	5	5	RZ422	RM288	9,629,862	12,289,109	189
MQTL_DRI9.2	9	82.94	5.48	12.71	10.72	4	4	V214	RM6475	12,289,109	12,838,988	57
MQTL_GY9.4	9	84.70	1.68	15.97	11.42	7	7	ME5_8	RM6475	18,562,785	19,788,313	206
MQTL_HD9.2	9	90.29	6.37	13.88	13.24	3	3	R742A	RG667	20,068,777	21,189,197	177
MQTL_BY9.2	9	92.45	2.73	15.68	11.08	5	5	ME9_3	RM6839	19,788,341	20,481,896	119
MQTL_PH10.1	10	18.50	7.35	13.74	12.62	3	3	S12669SA	Hst39	5,727,479	11,602,015	326
MQTL_GY10.1	10	26.30	7.70	24.09	11.72	4	4	S1837	S11069	11,602,015	14,025,364	250
MQTL_BY10.1	10	28.47	2.54	6.46	16.98	3	3	EM14_10	RM271	13,480,470	14,025,364	46
MQTL_DRI10.1	10	30.37	4.63	6.83	11.84	2	2	EM14_10	RM269	13,480,470	14,607,560	95
MQTL_PN10.1	10	32.77	6.01	14.24	12.04	4	4	RM271	RM4915	14,025,364	15,587,785	146
MQTL_HD10.1	10	33.55	2.71	3.82	11.80	2	2	RM1083	S21636S	14,605,820	15,261,951	64
MQTL_GY10.2	10	33.62	3.12	13.20	8.83	3	3	RM1083	S21636S	14,605,820	15,261,951	52
MQTL_PH10.2	10	65.16	9.10	14.64	8.30	2	2	C121	V37B	20,310,843	21,100,387	119
MQTL_GY10.3	10	65.58	2.67	20.17	8.88	6	6	RM228	RM496	22,243,253	22,430,227	30
MQTL_HD10.2	10	67.56	4.65	9.56	11.84	3	3	RM333	V37B	20,947,966	21,120,633	24
MQTL_GY11.1	11	17.14	1.77	15.72	13.35	4	4	Rcn1	RG98	2,449,218	3,041,250	79
MQTL_BY11.1	11	36.54	6.53	17.29	13.14	5	5	M136A	RM441	3,853,690	6,081,266	224
MQTL_PN11.1	11	48.76	3.37	10.19	13.33	3	3	RM54	RM47	8,210,074	8,985,276	35
MQTL_DRI11.1	11	59.74	6.43	5.68	19.47	2	2	E25M50.M004-P2	RM3428	9,068,620	13,481,859	459
MQTL_BY11.2	11	65.52	4.06	16.04	10.57	4	4	L1067	RZ797	14,276,646	18,468,941	239
MQTL_GY11.2	11	76.32	1.86	13.17	16.71	3	3	RM21	E60570	17,353,970	17,985,194	37
MQTL_PH11.1	11	90.9	7.18	25.15	9.37	4	4	RG103	E1981S	20,337,358	21,994,183	158
MQTL_GY11.3	11	100.0	17.34	33.13	7.32	3	3	RG2	RM2884	18,320,004	24,850,396	596
MQTL_HD12.1	12	58.38	2.24	6.11	9.16	2	2	RM260	RM313	15,327,972	15,776,997	9
MQTL_PN12.1	12	61.31	1.45	2.25	22.50	2	2	EM14_2	G1184F	15,120,656	16,193,811	65
MQTL_DRI12.1	12	61.75	2.14	16.56	11.07	4	4	EM14_2	bph2	15,120,656	16,193,811	65
MQTL_GY12.2	12	61.93	1.0	5.53	19.91	6	6	RM86	G1184C_4	15,120,656	16,193,811	36
MQTL_HD12.2	12	62.21	1.0	11.40	15.30	3	3	RM1261	bph2	17,566,476	18,827,749	79
MQTL_BY12.1	12	64.05	1.77	12.91	13.56	6	6	bph2	RM151	18,827,749	22,092,161	73
MQTL_PH12.1	12	71.17	4.43	34.77	3.05	4	4	CDO344	ME6_6	19,875,599	21,197,246	62
MQTL_FLZ12.1	12	89.94	5.67	14.28	12.0	3	3	R10289S	RM312	23,063,712	23,704,905	96
MQTL_DRI12.2	12	90.90	4.19	8.81	18.30	2	2	C2808	RM6947	23,318,032	23,974,182	87

^*^BY; biomass yield, CT; canopy temperature, DRI; drought response index, FLZ; flag leaf size, GY; grain yield, HD; heading/days to flowering, HI; harvest index, PH; plant height, PN; panicle number and SF; spikelet fertility

### Identification of DEGs Involved in Drought Stress Response of Rice

RNA-seq and microarray datasets were used to identifydrought-responsive genes in the rice (Additional file 8: Table S5). In total, 1.4814 and 23.722 genes were found to be drought responsive by RNA-seq and Microarray analysis (Additional file 13: Tables S10 and Additional file 14: S11 and Additional file 1: Fig. S1). On the other hand, MQTL regions identified to control DT-associated traits and yield components were investigated to discover the genes placed in those regions. Comparing the DEGs discovered by RNA-seq and microarray analysis with genes located within all 213 identified MQTL regions using Venn diagrams revealed the presence of 6,877 common genes. Furthermore, 375 genes were shared among the DEGs identified by RNA-seq and microarray analysis and genes within the 17 MQTLs with CI < 1 cM (Additional file 1: Fig. S1). The mentioned genes are recognized as differentially expressed candidate genes due to being both drought responsive and locating in MQTL regions.

### Estimating QTL-Overview Index for DT-Associated QTLs in Rice and Validating MQTLs Using GWAS Studies

To strongly associate genomic regions with yield components and DT associated traits in rice, the QTL-overview index was estimated. The QTL-overview index, representing QTL density, was calculated for every 0.5 cM segment on each chromosome (Additional file 2: Fig. S2). The findings revealed that 213 overview index peaks exceeded the genome-wide mean value (Chardon et al. 2004), showing the presence of real QTLs controlling yield components and DT-associated traits in rice (Additional file 2: Fig. S2). In addition, 113 peaks (out of the 213 peaks) were higher than the high-value threshold (Chardon et al. 2004) and were considered as QTL hotspots (Additional file 2: Fig. S2).

Significant concurrences were observed between the MQTLs identified through a comprehensive analysis and the SNPs uncovered via the GWAS approach concerning traits associated with DT within the rice genome. Notably, among the total of 213 identified MQTLs, 63 were found to be colocated with 130 peak SNP positions, which were detected through the GWAS approach for traits associated with DT in rice (Additional file 10: Table 7). Totally, 765 rice genes were identified in the SNP peak positions (± 25 kb) overlapped with MQTLs. Furthermore, 61 SNP peak positions (out of the 130 SNP peak positions) overlapped with high-overview-index MQTLs (QTL hotspots). In this study, we identified novel candidate genes among those situated within the significant SNP peaks and regions characterized by high QTL-overview indices associated with yield components and DT traits. Specifically, we discovered nineteen genes within SNP peak positions that coincided with QTL hotspots, thereby designating them as novel candidate genes (Fig. 3, Additional file 10: Table S7).

Conclusively, we integrated the results achieved by the meta-analysis of QTLs, GWAS studies, and the transcriptome data analysis leading to the discovery of 231 candidate genes (Additional file 3: Fig. S3, Additional file 15: Table S12), which might play key roles in rice DT and yield-associated traits under drought stress. In addition, 9 genes were common between GWAS studies results for DT and yield-related traits, DEGs discovered by RNA-seq and microarray analysis and the genes placed in the 17 MQTLs having CI < 1 cM (Additional file 3: Fig. S3, Additional file 15: Table S12). Following functional analysis, the identified candidate genes hold promise for applications in genetic engineering efforts targeting improvements in yield potential, stability, and performance under water deficit conditions, specifically focusing on DT and yield-related traits.

### Inclusive Meta-Analysis of Traits, QTL-Overview Index and Identification of Breeding MQTLs

Totally, 96 MQTLs were detected by inclusive meta-analysis of all the 1,087 original QTLs related to yield components and DT-associated traits in rice. The number of the detected MQTLs on each chromosome varied from 13 MQTL on chromosome 3 to 4 MQTLs on chromosome 9 (Fig. 3f; Table 4). The CI of the identified MQTLs was between 0.01 and 9 cM, having a mean of 2.33 cM, which was 4.66 times narrower than the CI average of the initial QTLs. Only 8 MQTLs (out of the 96 identified MQTLs) had a confidence interval > 5 cM. The CI values of 52 and 25 MQTLs were less than 2 and 1 cM, respectively (Table 4, Fig. 3). The number of the initial QTLs clustered in a MQTL ranged from 2 to 59 QTLs (Table 4). Forty MQTLs included more than 10 initial QTLs. For 71 MQTLs, the PVE mean of the initial QTLs was higher than 10 (Table 4, Fig. 3). There were 59 common MQTLs between the obtained results by inclusive meta-analysis for all the traits and meta-analysis for each trait (Fig. 3).

**Table 4:** Summary of the identified general MQTLs for yield components and drought tolerance associated traits under drought stress in rice

MQTL	Chr.	Position	CI (95%)	Mean CI of initial QTL	Mean variance of initial QTL (*R*^2^)	Initial QTL number	No. of study	Left marker	Right marker	Left position	Right position	Trait involved	Number of genes
MQTL1-1	1	7.80	1.50	6.32	15.39	5	4	RM1282	C1679	548,711	1,171,021	BY, GY, PH, SF	114
MQTL1-2	1	18.34	5.76	21.39	6.15	6	3	C804	RM3426	3,099,744	4,051,819	GY, PH, PN	135
MQTL1-3	1	36.59	9.00	29.67	6.76	8	8	RM7086	RM8142	6,192,801	7,457,624	BY, GY, HI, PH	175
MQTL1-4	1	75.26	0.40	6.50	10.59	4	2	RM6681	C585	15,898,001	17,584,841	GY	83
MQTL1-5	1	105.35	0.56	12.78	13.36	54	21	C409	RG233	24,941,627	27,050,163	BY, CT, HD, FLZ, HI, DRI, PH, SF, PN	250
MQTL1-6	1	108.44	0.77	6.79	12.64	27	8	C808	RM3475	25,637,999	26,041,116	BY, DRI, HD, GY, PN, SF, PH	44
MQTL1-7	1	114.79	1.58	5.62	25.66	8	4	C10728S	S1457	26,884,639	27,084,363	HD, DRI, PH	24
MQTL1-8	1	129.93	1.26	9.83	16.86	26	14	RM7643	RM8096	31,138,070	31,363,367	BY, HD, GY, DRI, PH, PN	30
MQTL1-9	1	137.96	2.24	4.56	14.77	3	1	R655	C11234S	32,597,267	34,148,691	GY	233
MQTL1-10	1	142.12	1.37	3.35	30.30	3	1	RM5811	R836	34,635,439	35,624,461	GY, PH	139
MQTL1-11	1	152.22	0.94	8.76	17.17	12	5	RM6696	S2523	38,220,799	38,511,992	DRI, GY, HI, PH, SF	52
MQTL1-12	1	161.72	0.02	11.48	22.41	20	4	RM1387	S877A	40,206,787	40,362,513	BY, HD, GY, DRI, PH, PN	17
MQTL2-1	2	30.80	1.11	10.00	11.86	27	16	RM6247	C365	5,797,995	6,586,865	BY, CT, DRI, FLZ, GY, PH, PN, SF	105
MQTL2-2	2	51.74	3.94	18.42	6.70	17	12	G1340	RM300	10,425,113	13,191,461	BY, DRI, FLZ, GY, PH, SF, PN	214
MQTL2-3	2	67.85	4.25	12.98	9.14	6	4	RM7413	E30251S	18,452,888	19,879,123	FLZ, GY, PH, PN	156
MQTL2-4	2	89.05	3.86	7.81	12.89	2	2	R3041	R1424	21,924,973	22,816,467	HD, GY	125
MQTL2-5	2	108.78	1.73	10.33	6.83	24	10	RM7245	RM6366	26,443,082	26,965,400	HD, FLZ, HI, DRI, SF, PN	70
MQTL2-6	2	120.6	1.73	13.19	8.26	27	14	RM6122	CDO686	28,429,802	30,916,361	BY, CT, DRI, HD, GY, HI, FLZ, PH, PN, SF	368
MQTL2-7	2	133.23	0.83	11.13	13.36	9	6	Lsi1	RM6519	31,262,234	33,828,030	BY, FLZ, GY, PN, SF	371
MQTL2-8	2	139.66	0.01	2.93	27.10	10	4	RM213	RM208	34,652,409	35,135,926	BY, GY, HD, HI, PH, SF, PN	79
MQTL3-1	3	4.83	0.62	8.12	20.80	7	4	C51477S	S12021	1,012,713	1,269,836	DRI, PH	32
MQTL3-2	3	14.4	1.84	11.11	7.65	11	2	RM6301	E60866S	2,651,426	3,658,280	HD, GY, DRI, BY	180
MQTL3-3	3	20.0	0.84	1.46	31.00	9	3	RM4352	RM3195	4,314,114	4,481,736	BY, FLZ, GY, PH, SF	32
MQTL3-4	3	25.43	5.53	7.57	8.32	10	2	RM6038	RM5755	4,828,438	5,958,909	BY, GY, FLZ, DRI, PH, SF	178
MQTL3-5	3	45.16	0.64	2.39	26.72	28	4	RG409	RM489	3,475,375	4,333,835	BY, CT, HD, GY, HI,	147
MQTL3-6	3	46.51	0.97	1.55	33.80	10	2	RM6496	R1690	10,141,494	10,424,161	BY, CT, GY, PH, PN	50
MQTL3-7	3	47.66	0.33	11.81	15.33	59	18	S804	S1828	10,444,810	10,809,230	HD	53
MQTL3-8	3	61.64	5.86	8.53	10.49	2	2	RM5928	RM6594	12,574,977	14,096,202	GY, SF	151
MQTL3-9	3	70.50	0.29	9.77	11.90	6	4	R2982	C11260S	15,212,945	15,471,990	BY, HD, GY, HI, BY	18
MQTL3-10	3	91.14	1.85	8.88	17.16	2	4	L481	RZ403	22,816,752	23,088,637	BY, GY	25
MQTL3-11	3	98.53	1.65	12.16	11.10	9	5	R19	R250	24,595,174	25,117,296	BY, DRI, HD, FLZ, GY, HI, PH	59
MQTL3-12	3	103.71	4.21	12.86	7.79	6	2	RZ761	RM468	29,923,232	32,675,044	DRI, PH	454
MQTL3-13	3	125.62	1.44	24.38	9.20	7	6	C1351	RM1350	28,351,568	28,676,461	HD, GY, DRI, PH	37
MQTL4-1	4	24.05	1.50	13.42	13.44	14	11	V145	C10543S	13,694,349	14,669,542	BY, DRI, HD, FLZ, GY, PH, SF	51
MQTL4-2	4	41.67	2.59	11.18	12.52	6	4	C12216S	E61747S	18,391,172	18,674,598	GY, DRI, PN	28
MQTL4-3	4	50.66	3.27	8.57	14.93	8	3	S10644	C975	19,053,076	19,733,943	BY, DRI, PN	72
MQTL4-4	4	61.82	4.78	14.39	7.45	12	4	RM3397	RM3337	20,452,466	21,732,917	BY, GY, HI	146
MQTL4-5	4	69.62	2.76	12.22	12.26	10	6	RM3524	C12247S	22,708,799	23,187,177	BY, CT, GY, HD, PN, SF	86
MQTL4-6	4	86.49	3.12	8.33	16.09	7	3	RM5714	RM451	27,286,876	28,386,220	HD, PH, PN, SF	153
MQTL4-7	4	94.34	3.92	12.02	9.87	11	7	RM303	C79	28,574,911	29,304,018	BY, CT, FLZ, PN, SF	87
MQTL4-8	4	105.92	2.12	16.40	12.78	33	12	RG329	RM1100	30,853,328	31,442,408	BY, FLZ, GY, HD, DRI, PH, PN, SF	104
MQTL4-9	4	134.96	1.03	6.99	12.60	3	1	RM6303	G177	35,112,751	35,502,694	GY, PN, SF	62
MQTL5-1	5	7.35	2.52	3.57	10.50	2	1	RM5693	S1548	463,910	742,277	HD	45
MQTL5-2	5	30.97	2.53	12.00	11.06	9	7	S12447	C61983S	3,500,388	4,331,034	BY, HD, GY, DRI, PH, BY, SF	90
MQTL5-3	5	51.28	5.36	11.94	12.25	4	3	RM7293	RM3381	7,526,843	9,585,978	BY, GY, PN	125
MQTL5-4	5	66.06	3.29	16.28	9.69	5	4	E2801S	RM6024	16,960,667	17,752,315	CT, GY, DRI, PN	77
MQTL5-5	5	74.27	2.02	15.99	13.23	21	7	RM430	RZ649	18,691,482	19,545,619	BY, DRI, HD, FLZ, GY, PN	99
MQTL5-6	5	83.68	2.49	11.91	9.44	7	7	R1320	C308	20,744,914	21,135,716	HD, FLZ, DRI, PH, SF	49
MQTL5-7	5	109.82	2.75	16.28	10.94	5	3	RM3790	RM6952	26,195,524	27,321,197	PH, SF, BY	186
MQTL6-1	6	8.93	2.08	7.50	14.13	10	4	RM588	C688	1,611,462	2,198,480	BY, HD, GY, HI, DRI, PH, PN	105
MQTL6-2	6	13.53	0.97	3.38	19.78	4	1	RM8125	AID1	3,168,470	4,016,646	HD, GY	142
MQTL6-3	6	16.78	0.77	8.29	10.84	56	8	RM204	RM225	3,168,461	3,416,631	BY, HD, FLZ, HI, PH, PN, SF	43
MQTL6-4	6	21.94	0.05	3.26	30.04	8	1	RZ450	RM314	4,212,619	4,845,268	HD, GY, HI, DRI, PN	90
MQTL6-5	6	33.52	8.86	18.03	7.96	3	3	RG213	C1478	6,283,659	6,822,392	GY, PH, BY	73
MQTL6-6	6	57.77	2.76	22.70	10.33	18	10	C235	RM527	9,282,848	9,874,178	HD, GY, PH, BY	33
MQTL6-7	6	67.45	4.29	10.29	9.34	6	4	C288B	RM275	18,498,356	23,942,122	FLZ, GY, HI, PN, SF, HD	438
MQTL6-8	6	74.16	2.08	6.55	12.00	2	2	RM3187	G2028	20,579,037	22,609,459	GY, DRI	190
MQTL6-9	6	91.65	2.50	13.60	8.81	15	7	RM5957	RM8239	24,138,957	24,555,015	CT, DRI, GY, HI, PH, SF, BY	64
MQTL6-10	6	107.13	1.52	13.29	10.54	6	4	E4392S	RM400	27,377,223	28,431,750	BY, DRI, HD, PH, BY	137
MQTL7-1	7	49.22	2.76	15.11	7.67	16	9	S21852S	RG30	8,333,742	12,786,724	CT, GY, HD, DRI, FLZ, PH, SF, PN	210
MQTL7-2	7	62.89	4.44	12.26	8.81	3	2	G20	RM432	17,526,630	18,958,690	GY	123
MQTL7-3	7	70.36	2.84	14.52	7.40	11	5	RM11	RM3826	19,256,977	20,807,448	BY, CT, HD, GY	155
MQTL7-4	7	89.75	1.68	17.21	10.13	14	9	E3930S	RM3589	24,809,203	25,107,169	HD, GY, DRI, PN, SF	34
MQTL7-5	7	99.58	0.04	7.45	24.20	2	2	RM5455	R2358	26,461,046	26,637,818	FLZ, HI	26
MQTL8-1	8	18.91	2.27	12.16	12.96	12	8	R3786	RZ143	680,624	1,527,864	HD, GY, PN, SF, PH, BY	117
MQTL8-2	8	48.87	3.69	16.18	10.60	5	3	RM3231	S1850A	3,838,208	4,999,051	GY, HD	112
MQTL8-3	8	60.50	2.40	3.49	13.00	2	1	RM3181	RM6429	7,552,919	8,384,391	HD	76
MQTL8-4	8	65.80	0.98	14.75	13.37	22	12	E3835S	E40306S	9,432,624	10,286,626	BY, CT, GY, HD, DRI, PN, SF	44
MQTL8-5	8	68.45	3.70	5.82	18.45	2	1	RM331	RM7027	12,294,440	15,844,928	GY,	160
MQTL8-6	8	80.01	1.74	7.29	18.36	11	6	RM3689	R727	18,818,894	19,337,282	CT, DRI, GY, PH, BY	43
MQTL8-7	8	84.31	1.52	4.85	15.82	4	3	RM8264	RM1109	19,833,287	20,483,338	GY, BY	63
MQTL8-8	8	91.42	1.76	11.44	10.55	26	14	RM7049	RG1	20,812,460	21,647,205	CT, DRI, HD, GY, HI, PH, PN, SF	92
MQTL8-9	8	107.47	0.26	12.58	16.17	13	3	RM6976	R202	23,555,676	24,185,871	BY, GY, PH, PN	85
MQTL9-1	9	47.81	1.39	14.45	12.12	26	9	RM8206	R10783S	5,919,594	6,387,633	BY, HD, FLZ, GY, HI, DRI, PH, SF	29
MQTL9-2	9	69.99	1.31	10.71	9.27	9	6	RM409	RM566	14,372,099	14,704,835	GY, PH, PN	47
MQTL9-3	9	81.68	2.93	13.31	11.45	14	8	RM257	RM288	17,719,767	18,562,785	CT, GY, DRI, BY	124
MQTL9-4	9	92.43	0.37	16.63	10.51	15	11	S4677S	RM5657	13,625,363	14,366,848	BY, CT, DRI, HD, DRI, PH, PN, SF, GY	71
MQTL10-1	10	19.40	5.04	11.74	13.17	5	4	C63979S	S3578S	9,921,318	11,579,066	CT, HD, PH, PN	125
MQTL10-2	10	27.66	2.36	12.60	12.43	10	7	RM596	RZ625	15,209,035	16,620,262	BY, DRI, FLZ, GY, PH	138
MQTL10-3	10	33.45	1.87	6.81	12.17	8	5	C1633	Y1053R	15,135,545	16,067,185	HD, GY, DRI, BY, PN	113
MQTL10-4	10	40.01	5.41	12.18	9.46	5	5	Y1053L	C1286	16,345,398	17,134,742	BY, GY, PH, PN, SF	76
MQTL10-5	10	65.84	0.27	18.75	9.39	16	13	RM333	RM496	21,924,145	21,982,201	CT, HD, FLZ, GY, FLZ, HI, DRI, PH	13
MQTL11-1	11	16.71	1.74	14.43	11.66	5	2	R1938	RM1812	2,339,661	2,405,238	HD, GY	12
MQTL11-2	11	34.38	4.26	14.99	13.92	7	5	S20163S	S21074S	5,375,533	5,601,997	BY, GY, DRI, PN	23
MQTL11-3	11	49.58	3.27	12.91	14.57	7	5	RM479	S2137	7,692,852	8,296,761	HD, GY, DRI, PN, BY	55
MQTL11-4	11	60.10	3.80	6.61	19.67	3	2	RG247	E61044S	10,132,148	13,042,924	DRI, PN, BY	344
MQTL11-5	11	69.87	3.30	7.40	13.07	5	4	R10329S	RM457	18,333,609	19,064,898	GY, DRI, BY	46
MQTL11-6	11	76.66	1.81	4.67	21.82	4	2	G257	E60570	19,646,583	20,277,407	GY, PH	58
MQTL11-7	11	95.21	4.03	22.95	9.13	9	5	C189	R2458	23,734,008	24,909,396	CT, HD, GY, PH, SF, BY	94
MQTL11-8	11	113.29	0.94	9.64	11.20	3	3	L833	C61639	27,661,357	28,153,714	HD, PH, BY	42
MQTL12-1	12	46.82	2.92	9.09	12.71	5	5	RG869	C61563S	7,729,610	8,670,795	HD, GY, SF, BY	42
MQTL12-2	12	55.40	0.93	5.34	17.56	11	6	S13126S	S10043S	15,120,656	15,327,972	GY, HI, DRI, BY, PH	9
MQTL12-3	12	59.19	2.53	3.26	12.50	2	1	RM1261	S1436	16,344,411	17,531,220	HD, PN	38
MQTL12-4	12	61.89	0.58	9.98	17.58	20	8	E4418S	C50732S	17,566,476	19,000,965	BY, HD, GY, HI, DRI, PN, SF, PH	76
MQTL12-5	12	67.84	2.05	6.48	9.00	3	1	S21024S	CDO344	19,491,413	23,603,292	BY, HD, PH	344
MQTL12-6	12	90.89	0.24	12.48	15.26	8	7	RM7376	RM1103	23,443,538	23,606,725	DRI, FLZ, HD, PH, SF	27

After mapping the 1,087 QTLs on the consensus genetic map, the QTL density, described as the "QTL-overview index", was calculated for a 0.5 cM distance on each chromosome to find genomic regions significantly related to the studied traits (Additional file 2: Fig. S2 a). Ninety-six overview index peaks were attained, which were exceeded 0.0043 as the average of the genome-wide statistic and indicated the “Real QTLs” affecting all yield traits in rice. Based on the Additional file 2: Fig. S2 a, out of the 96 peaks considered as “Real QTLs”, 49 peaks overpassed 0.0219 as the high-value threshold and thus can be considered as “QTL hotspots”.

According to the criteria presented by Löffler et al. 2009, an MQTL with a narrow confidence interval, a high number of the original QTLs and a high PVE value for the original QTLs are considered suitable for marker-assisted breeding (Löffler et al. 2009). Based on the achieved results, 13 MQTLs with CI < 1 cM, sum of the original QTLs > 10 and mean PVE value of the original QTLs > 10, identified using the inclusive MQTL analysis of all the investigated traits, can be considered as “Breeding MQTLs” (Table 5, Fig. 3). Interestingly, these thirteen MQTL regions are located in the QTL hotspots.

**Table 5:** The MQTLs with more than 10 initial QTLs, CI < 1 cM and a PVE average of the original QTLs > 10, which were considered as “Breeding MQTLs”

MQTL	Chr.	Position	CI (95%)	Mean CI of initial QTL	Mean variance of initial QTL (*R*^2^)	Initial QTL number	No. of study	Left marker	Right marker	Left position	Right position	Trait involved	Number of genes
MQTL2-8	2	139.66	0.01	2.93	27.10	10	4	RM213	RM208	34,652,409	35,135,926	BY, GY, HD, HI, PH, SF, PN	79
MQTL1-12	1	161.72	0.02	11.48	22.41	20	4	RM1387	S877A	40,206,787	40,362,513	BY, HD, GY, DRI, PH, PN	17
MQTL8-9	8	107.47	0.26	12.58	16.17	13	3	RM6976	R202	23,555,676	24,185,871	BY, GY, PH, PN	85
MQTL9-4	9	92.43	0.37	16.63	10.51	15	11	S4677S	RM5657	13,625,363	14,366,848	BY, CT, DRI, HD, DRI, PH, PN, SF, GY	71
MQTL1-5	1	105.35	0.56	12.78	13.36	54	21	C409	RG233	24,941,627	27,050,163	BY, CT, HD, FLZ, HI, DRI, PH, SF, PN	250
MQTL12-4	12	61.89	0.58	9.98	17.58	20	8	E4418S	C50732S	17,566,476	19,000,965	BY, HD, GY, HI, DRI, PN, SF, PH	76
MQTL3-5	3	45.16	0.64	2.39	26.72	28	4	RG409	RM489	3,475,375	4,333,835	BY, CT, HD, GY, HI,	147
MQTL6-3	6	16.78	0.77	8.29	10.84	56	8	RM204	RM225	3,168,461	3,416,631	BY, HD, FLZ, HI, PH, PN, SF	43
MQTL1-6	1	108.44	0.77	6.79	12.64	27	8	C808	RM3475	25,637,999	26,041,116	BY, DRI, HD, GY, PN, SF, PH	44
MQTL12-2	12	55.40	0.93	5.34	17.56	11	6	S13126S	S10043S	15,120,656	15,327,972	GY, HI, DRI, BY, PH	9
MQTL1-11	1	152.22	0.94	8.76	17.17	12	5	RM6696	S2523	38,220,799	38,511,992	DRI, GY, HI, PH, SF	52
MQTL3-6	3	46.51	0.97	1.55	33.80	10	2	RM6496	R1690	10,141,494	10,424,161	BY, CT, GY, PH, PN	50
MQTL8-4	8	65.80	0.98	14.75	13.37	22	12	E3835S	E40306S	9,432,624	10,286,626	BY, CT, GY, HD, DRI, PN, SF	44

### Candidate Genes with Inclusive/Distinct Analysis of Traits

**Fig. 4:**
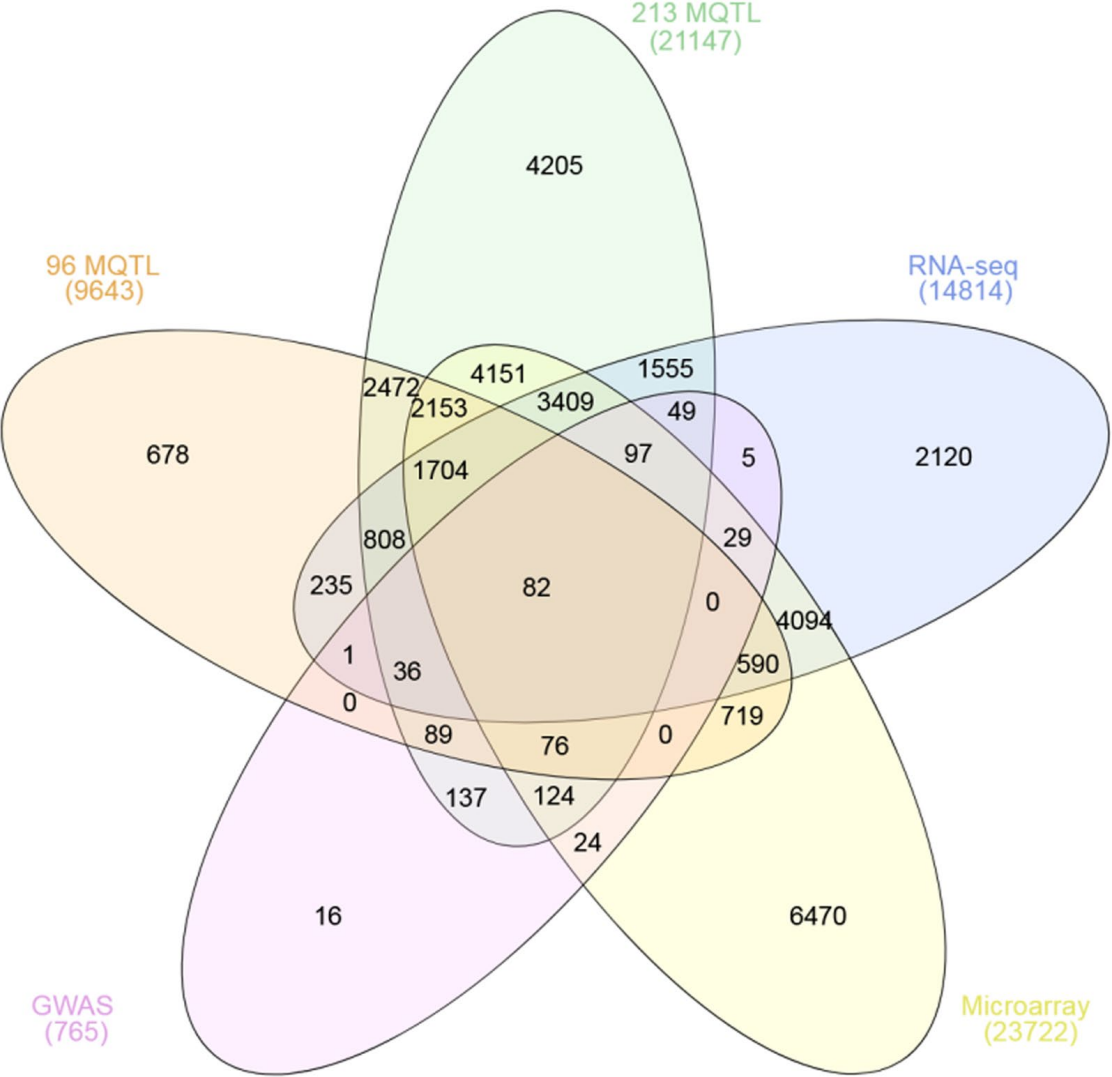
Venn diagram showing the genes placed in the detected MQTLs through inclusive MQTL analysis on all the traits (orange) and distinct MQTL analysis for each individual trait (green), genes placed in SNP peak positions based on GWAS studies (pink) for yield and DT-associated traits under drought stress conditions, and the drought responsive genes based on RNA-seq (blue) and microarray (yellow) data

Combining the results of MQTL analysis for yield and DT-related traits (both inclusive and distinct analysis of traits), GWAS studies, and transcriptome data resulted in identification of 82 candidate genes (Fig. 4 and Additional file 16: Table S13). Among these candidate genes, two genes including *Os02g0700700* (*OsBRXL2*) and *Os04g0431200* (*OsDRF1*) are placed both on the SNP peak positions and in the high-overview-index MQTLs i.e. MQTL_BY2.2 and MQTL_GY4.2, respectively (Additional file 10: Table S7).

## Discussion

### Candidate MQTLs/Genes for Yield Maintenance Under Drought with Potential Application in Breeding Programs

One effective statistical approach for accurately detecting QTLs that control yield components and DT-associated traits in the genome could involve conducting a meta-analysis on a large number of independent QTLs associated with these traits. In this particular study, a meta-analysis was performed on 1,087 QTLs obtained from 76 different studies and 134 distinct rice populations, all related to yield components and DT-associated traits (Additional file 11: Table S8). This analysis led to the identification of 213 MQTLs (Table 3). The utilization of MQTL analysis resulted in a significant reduction in the CI, enabling the identification of a more precise set of candidate genes potentially involved in controlling the investigated traits. The average CI of MQTLs was 4.68 cM, representing a 2.74-fold decrease compared to the average CI of the original QTLs, which was 12.86 cM (Table 3). Notably, 62.4% of the MQTLs had a CI of less than 5 cM, while 17 MQTLs displayed a CI of less than 1 cM (Table 3). Apart from having a narrow CI, an MQTL selected for breeding should possess a high number of original QTLs and a high PVE value. In this study, 17 MQTLs (including MQTL_PH1.2, MQTL_GY6.4, MQTL_GY8.4, MQTL_DRI1.1, MQTL_SF4.2, MQTL_HD6.2, MQTL_GY8.5, MQTL_PH1.4, MQTL_BY6.1, MQTL_GY6.2, MQTL_PN4.4, MQTL_GY3.3, MQTL_HI3.2, MQTL_GY2.4, MQTL_GY2.1, MQTL_HD3.5, and MQTL_GY1.4) fulfilled these criteria, with more than 10 original QTLs, an average PVE of 14.2, and a mean CI of 1.98 cM. Consequently, they can be regarded as potential MQTLs for future breeding programs aimed at enhancing yield and drought tolerance in rice.

**Fig. 5:**
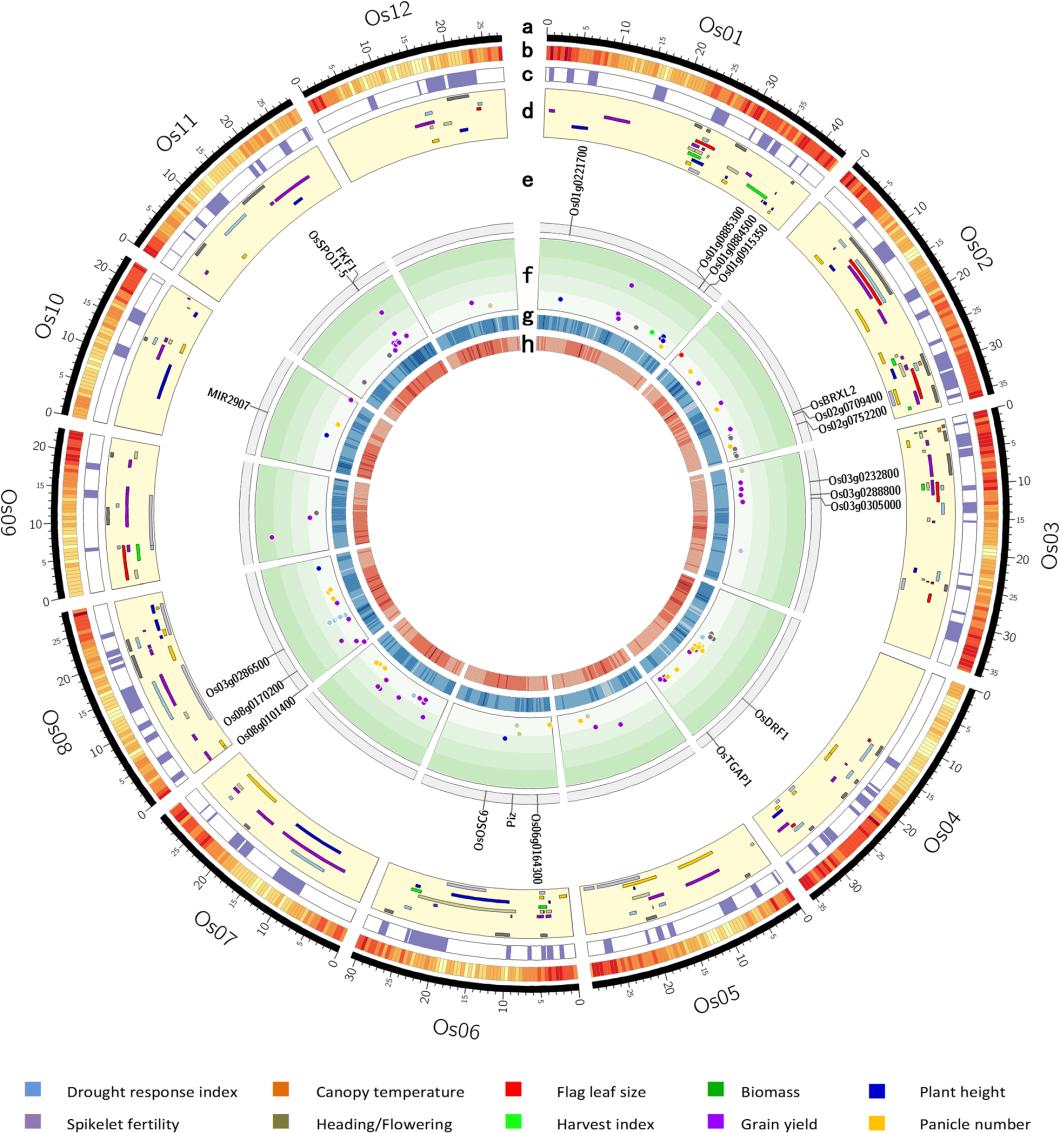
The Circos diagram illustrates the positioning of MQTLs and GWAS-based SNPs associated with yield and drought tolerance (DT)-related traits under water deficit conditions on the physical map of *Oryza sativa* japonica (Nipponbare). **a** The twelve rice chromosomes are arranged in a clockwise direction. **b** The gene density on rice chromosomes shown in yellow to red scale for the lowest to the highest density in 500 kb windows. **c** The locations of identified MQTLs through comprehensive MQTL analysis on the physical map of each chromosome. **d** Position of the identified MQTLs for each distinct trait on the physical map of each chromosome. **e** Showing the genes locating inside both the SNP peak positions and the QTL-overview peaks for yield and DT-associated traits under drought stress conditions as novel candidate genes (Supplementary Table S7). **f** Points having various colors symbolize significant SNPs discovered through GWAS studies for yield and DT-associated traits under water deficit conditions. **g** The heatmap representing the Indels density in white to dark blue color scale for the lowest to the highest density. **h** The heatmap representing the structural variants (SV) density in white to dark red color scale for the lowest to the highest density. Physical positions of all markers (including those used in the flanking markers each MQT and map markers) were determined using the genome assembly IRGSP-1.0

To identify candidate genes involved in controlling yield components and DT-related traits in rice, the genes located within the MQTL regions were compared with differentially expressed genes (DEGs) identified through RNA-seq and microarray data analysis (Additional file 9: Table S6). The genes responsible for regulating various investigated traits are discussed below. Furthermore, following the criteria defined by Löffler et al. in 2009, MQTLs exhibiting a high number of original QTLs, a high PVE value, and a narrow CI were identified as "Breeding MQTLs". Additionally, QTL-overview peaks that overlapped with SNP peaks reported in GWAS studies were considered, and the genes situated within these regions were recognized as potential candidate genes involved in controlling yield components and DT-related traits (Fig. 5, Table 3, and Additional file 10: Table S7).

### MQTLs and Candidate Genes for Grain Yield

Drought stress during the reproductive stage of rice leads to a significant reduction in grain yield (Palanog et al. 2014). According to the obtained results, 50 MQTLs were identified for the GY trait (Table 3). Chromosomes 1, 2, 3, 6 and 8 had the highest number of GY-associated MTQLs with 6 MTQLs per chromosome. The most stable GY-associated MQTLs were MQTL_GY1.4, MQTL_GY3.4, MQTL_GY2.1, MQTL_GY2.4, MQTL_GY3.3, MQTL_GY6.2, MQTL_GY8.5, MQTL_GY6.4, MQTL_GY8 having the uppermost number of original QTLs from 22, 17, 14, 13, 12, 11, 11, 10 and 10 different studies, respectively. The confidence interval of 8 MQTLs including MQTL_GY2.6, MQTL_GY1.6, MQTL_GY6.5, MQTL_GY3.6, MQTL_GY8.6, MQTL_GY3.3, and MQTL_GY1.4 was decreased to less than 1 cM (Table 3), indicating the potential of the mentioned MQTLs to be used in breeding programs to enhance GY under drought stress in rice.

The genes located within each MQTL interval are listed in Additional file 6: Table S3. One of these genes, *OsTF1L*, located within the MQTL_GY8.3 interval, is engaged in controlling GY in rice under drought stress treatment. Transgenic plants overexpressing *OsTF1L* indicated more tolerance to drought stress in comparison with wild type plants at the reproductive stage. In addition, transgenic plants produced higher GY compared to wild type plants under drought stress. Encouraging lignin biosynthesis and stomatal closure by the HD-Zip transcription factor of *OsTF1L* is the reason for improved drought tolerance in transgenic plants (Bang et al. 2019).

*OsCBL8* gene, detected in MQTL_GY2.2 interval, is engaged in rice response to abiotic stresses. Up- and down-regulation of *OsCBL8* in sense (salt tolerant) and anti-sense (drought tolerant) transgenic lines resulted in a remarkable reduction in both the number of filled grains per panicle and the seed setting rate in rice (GU et al. 2010).

*OsGRF4* gene, located in MQTL_GY2.5, encodes a transcriptional regulator and is regulated by *OsmiR396c*. It has been reported that the module of *OsmiR396c-OsGRF4-OsGIF1* is involved in determining GY and size in rice. It was demonstrated that a 2 bp substitution mutation in *OsGRF4* disturbs its regulation by *OsmiR396c*, leading to enhanced GY through increasing the weight and size of grains. In addition, there is direct interaction between *OsGRF4* and *OsGIF1* and it has been reported that overexpression of *OsGIF1* enhanced grain size (Li et al. 2016). Furthermore, increased expression of *OsGRF4* caused by mutation, activates brassinosteroid responses, promoting grain development (Hu et al. 2015).

*OsLHT1,* discovered in the MQTL_GY8.1 interval, encodes Lysine-Histidine-type Transporter 1, which is involved in translocating amino acids from vegetative organs to reproductive ones, determining GY. High GY and N use efficiency are determined by the suitable allocation of nitrogen from source leaves to grains. Panicle length, the grain number per panicle and total grain weight were reduced in knockout mutants of *OsLHT1* (Guo et al. 2020). *SAPK2,* located within MQTL_GY7.4 interval, encodesfor a serine/threonine-protein kinase and is able to increase GY via regulating nitrogen use efficiency under drought stress in the reproductive stage. In addition, contents of nitrogen, phosphorus, and potassium in rice grain are remarkably influenced by *SAPK2* (Lou et al. 2020). *RL9* (*SLL1*, *AH2*, *OsADD1*), found in MQTL_GY9.2 interval, codes for a MYB domain protein that is involved in the development of hull and grain. *RL9* influences on GY, grain size and quality. Rice plants overexpressing *SLL1* had longer lateral roots, indicating the potential of *SLL1* gene to be used for improving root architecture in rice (Ren et al. 2019; Shelley et al. 2013). *RAG2*, located in MQTL_GY7.1 interval, codes for a 14–16 kDa α-amylase/trypsin inhibitor. Overexpression of *RAG2* led to improved GY and grain quality in rice (Zhou et al. 2017). *PLANT ARCHITECTURE AND YIELD 1* (*PAY1*), located in MQTL_GY8.4 interval, plays a role in enhancing plant architecture and GY in rice. This gene can be used for establishing perfect plant architecture and breeding rice varieties for high yield (Zhao et al. 2015). *OsNPF7.2*, located in MQTL_GY2.5 interval, which codes for a nitrate transporter, plays a positive role in regulating number of tillers and GY in rice (Wang et al. 2018). *OsbHLH107* is located in MQTL_GY2.6 interval, and its homologs play important roles in regulating grain size development and can be used for improving GY in rice (Yang et al. 2018b). *OsABCG18,* detected in MQTL_GY8.2 interval, codes for an ABC transporter and is involvedin controlling cytokinins transport into shoots and GY in Rice. Enhanced cytokinins in the shoot and increased GY were obtained by overexpression of *OsABCG18* (Zhao et al. 2019).

Among the 50 MQTLs identified for the grain yield trait, a total of 22 MQTLs overlapped with 49 SNP peak positions associated with yield-related traits based on GWAS studies (Fig. 5, Additional file 10: Table S7). The genes located both in QTL-overview and SNP peaks, including *OsSPO11-5*, *OsDRF1*, *FKF1, Os03g0197175*, *Os03g0197200*, *Os03g0305000*, *Os03g0305050, Os03g0232800* and *Os08g0170200* are considered as candidate genes for GY under drought stress.

### MQTLs and Candidate Genes for Heading Date

Heading date, which is regulated by numerous environmental signals and endogenous cues, plays a crucial role in crop reproduction, yield, and regional adaptability (Wei et al. 2020). Yield and drought tolerance are highly correlated with HD (Xu et al. 2018). We identified 27 MQTLs for HD under drought conditions, with a maximum of 7 MQTLs on chromosome 3, and 4 MQTLs on each of the chromosomes 1 and 6 (Table 3). The highest number of original QTLs (18 QTLs) was observed for MQTL_HD3.5 on chromosome 3 (Table 3). In five MQTLs including MQTL_HD3.5, MQTL_HD3.7, MQTL_HD3.3, MQTL_HD3.1 and MQTL_HD12.2, the CI was decreased to less than 1 cM (Table 3). Out of the 27 identified MQTLs for the HD trait, 4 MQTLs overlapped with 6 SNP peak positions reported by GWAS studies and one MQTL (MQTL_HD3.1) was recognized as QTL-overview peaks overlapped with SNP peaks reported by GWAS studies (Fig. 5, Additional file 10: Table S7).

Some of the genes located in HD-related MQTLs are discussed here. *OsMFT*, located in the MQTL_HD6.4 interval, increased drought tolerance in rice by interacting with *OsbZIP66* and *OsMYB26,* recognized as main drought-related transcription factors, and regulating their binding to drought-responsive genes (Chen et al. 2021). *OsMFT1* suppressed *Ehd1*, *FZP,* and *SEPALLATA*-like genes, resulting in delayed heading date and enhanced spikelets per panicle in rice (Song et al. 2018). *Ehd2* gene, located in the MQTL10-1 interval, is involved in adjusting flowering time in rice (Brambilla and Fornara 2013). *Ehd2* gene was also detected on a HD-related MQTL on chromosome 10 under drought stress conditions in rice by Khahani et al. 2021 (Khahani et al. 2021).

### MQTLs and Candidate Genes for Plant Height

For the PH trait under drought stress in rice, 23 MQTLs were obtained. Chromosome 1 with 7 MQTLs had the most number of MQTLs per chromosome, followed by chromosomes 6 and 8 with 3 MQTLs per each chromosome. The most stable MQTLs for PH were MQTL_PH1.4, MQTL_PH1.2 and MQTL_PH1.7. MQTL_PH1.4 and MQTL_PH1.2 were considered as the most stable MQTLs for PH, because of having the most number of original QTLs with 10 and 11 original QTLs, respectively. MQTL_PH1.7 was identified as one of the most stable MQTLs due to having CI < 1 cM (Table 3).

Some of the genes located in PH-related MQTLs are discussed here. Auxin is involved in regulating plant height (Ma et al. 2016b). Amidase is engaged in the metabolic pathway of indole acetic acid (IAA). The crucial role of IAA phytohormone in cell division, differentiation, elongation, root development and plant height regulation has been reported (Petersson et al. 2009). In a prior investigation, specific genes associated with the auxin metabolic pathway, namely *OsYUCCA1*, *OsYUCCA8*, *WOX6*, and *OsRR2*, were identified within the genetic interval linked to MQTLs that are correlated with root system architecture in rice. (Daryani et al. 2022). In the current research, the same MQTLs and genes were detected for PH trait. This means that the common MQTLs and genes are involved in controlling both traits of root system architecture and PH in rice. In this study, *YUCCA1*, *YUCCA6*, *YUCCA4*, *OsIAA20*, *OsIAA21*, *OsIAA2*, *IAA6* and *OsRR33* were detected in the intervals of MQTL_PH1.3, MQTL_PH7.1, MQTL_PH1.1, MQTL_PH6.1, MQTL_PH6.2, MQTL_PH1.1, MQTL_PH1.4 and MQTL_PH8.2, respectively.

Another detected gene is *OsFTL1*, which locates in MQTL_PH1.1 interval. Pleiotropic effects of *OsFTL1* on the total number of secondary rachides, grains number per panicle, plant height and flag leaf length have been reported (Wang et al. 2020). *OsbZIP49*, detected in MQTL_PH6.3 interval, codes for a transcription factor that is engaged in controlling tiller angle and plant architecture via IAA synthetase. Overexpression of *OsbZIP49* in rice led to tiller-spreading phenotype, reduced plant height and internode length (Ding et al. 2021).

Out of the 23 identified MQTLs for PH trait, seven MQTLs overlapped with 14 SNP peak positions found by GWAS studies. Two novel candidate genes for PH trait under drought stress in rice, including *Os01g0884500* and *Os01g0885300* were located both in QTL-overview and SNP peaks (Fig. 5, Additional file 10: Table S7)*.*

### MQTLs and Candidate Genes for Biomass Yield

Enhancing biomass in rice is a key breeding objective, yet it poses challenges due to the intricacies involved and labor-intensive nature of the trait phenotyping (Matsubara et al. 2016). Twenty-nine MQTLs were detected for BY. Chromosomes 1, 3, and 4 each contain four MQTLs, while chromosomes 2 and 6 each harbor three (Table 3). The most stable MQTLs detected for BY under drought stress included MQTL_BY6.1, MQTL_BY3.3, MQTL_BY4.2 and MQTL_BY4.4, having the highest number of original QTLs and two MQTLs of MQTL_BY1.2 and MQTL_BY2.3, having CI of less than 1 cM (Table 3).

*OsOFP6*, detected within the MQTL_BY2.2 interval, is involved in regulating growth and development, and responses to drought and cold stresses in rice. RNAi-mediated knockdown of *OsOFP6* led to semi-dwarf plants with changed grain shape and shorter lateral roots. In addition, slower water loss and less accumulation of H2O2 were observed in *OsOFP6* overexpressing plants in comparison with RNAi plants under drought stress, indicating the role of *OsOFP6* in both drought avoidance and drought tolerance in rice. As well, a thicker secondary cell wall with enhanced lignin content was noticed in *OsOFP6* overexpressing plants in rice (Ma et al. 2017; Sun et al. 2020). *OsHk6 (OsCKT1)*, located within the MQTL_BY2.2 interval, serves as a cytokinin receptor and plays a role in regulating various biological processes such as secondary metabolism, sucrose and starch metabolism, chlorophyll synthesis and photosynthesis (Ding et al. 2017). In addition, green pigmentation and shoot induction were promoted in rice calli by ectopic expression of *OsHk6* (Choi et al. 2012)*.* Two genes encoding for glutamine synthetases, *OsGS1;1* and *OsGS1,* both located within MQTL_BY2.2 interval, are probably engaged in drought tolerance in rice based on the previous studies. *OsGS1;1* plays a key role in normal growth and grain filling under water deficit conditions. Increased physiological tolerance and agronomic performance were obtained in rice plants co-overexpressing *OsGS1;1* and *OsGS2* isoforms under adverse abiotic stress conditions (James et al. 2018; Tabuchi et al. 2005). *OsBRI1 (D61, OsBRKq1)*, detected within the MQTL_BY1.3 interval, is engaged in several growth and developmental processes like internode elongation, lamina joint bending and skotomorphogenesis. Altogether, the kinase activity of *OsBRI1* is crucial for brassinosteroids to regulate normal plant growth and development in rice (Zhao et al. 2013). *OsBRKq1* has the potential to be utilized for increasing yield through enhancing grain size in rice. Additionally, *OsBRKq1* was found on chromosome1 through a QTL mapping analysis for 1000 kernel weight, kernel length, and kernel width conducted on SNDH113 populations in which grain sizes were diversely distributed (Park et al. 2021). Expression of rice *OsARGOS* (detected in MQTL_BY4.3) in Arabidopsis resulted in enhancedcell division and expansion and increased organ size. Considering the role of *OsARGOS* in organ enlargement, it has the potential to be used for biomass enhancement through genetic engineering (Wang et al. 2009).

Ten MQTLs (Out of the 29 detected MQTLs for BY trait) overlapped with 17 SNP peak positions discovered by GWAS studies (Fig. 5, Additional file 10: Table S7). Three candidate genes including *OsBRXL2*, *DEK1* (*ADL1*) and *Os06g0164300* were identified for PH trait under drought in rice (Fig. 5, Additional file 10: Table S7) that were located in SNP peak positions overlapped with QTL-overview peaks MQTLs.

### MQTLs and Candidate Genes for Canopy Temperature

Canopy temperature under stress conditions is a reliable predictor of GY performance (Melandri et al. 2020). We identified two MQTLs for CT consisting of one MQTL on chromosome 2 and another on chromosome 3 (Table 3).

### MQTLs and Candidate Genes for Drought Response Index

Twenty-four MQTLs were identified for DRI using the meta-analysis of QTLs. The highest count number of DRI-associated MQTLs per chromosome was 3 and related to chromosomes 2, 3, and 5 (Table 3). MQTL_DRI1.1 was recognized as the most stable DRI-related MQTL having the most number of original QTLs from 10 independent studies. *GF14c,* located in MQTL_DRI8.2, codes for a 14-3-3 protein. 14-3-3 proteins play main roles in regulating primary metabolism and transducing cellular signals (Ho et al. 2013). It has been demonstrated that overexpression of *GF14c* resulted in increased drought tolerance in transgenic seedlings of rice (Ho et al. 2013). Another gene that was found in MQTL_DRI2.2 interval is *OsDi19-4* (Dehydration-induced 19 homolog 4). The *OsDi19* codes for proteins that are engaged in response to abiotic stresses. Wang et al. 2014 indicated that overexpression of *OsDi19-4* led to increased drought tolerance in rice through increasing ROS-scavenging activity (Wang et al. 2014). In addition, the expression of some ABA-responsive genes was changed in rice plants overexpressing *OsDi19-4* which resulted in strong ABA-hypersensitive phenotypes (Wang et al. 2016). *OsDIS1*, detected in MQTL_DRI3.2, encodes a SINA-type E3 ligase. *OsDIS1* plays a negative role in drought stress tolerance via regulating transcription of several stress-associated genes and probably via regulating its interacting protein *OsNek6* at posttranslational level in rice (Ning et al. 2011). *OsETOL1*, located in the MQTL_DRI3.1 interval, encodes a homolog of ETHYLENE OVERPRODUCER. Two allelic mutants of *OsETOL1* indicated enhanced tolerance to drought stress at panicle development stage (Du et al. 2014). *OsGRAS23*, detected in MQTL_DRI4.3 interval, codes for a stress-responsive GRAS transcription factor. *OsGRAS23* plays a positive role in regulating drought tolerance in rice by inducing several stress responsive genes (Xu et al. 2015). *OsMT1a* (a type 1 metallothionein), found in MQTL_DRI12.2 interval, is engaged in zinc homeostasis and drought tolerance in rice. Yang et al. 2009 indicated that overexpression of *OsMT1a* resulted in increased drought tolerance in rice via taking part in ROS scavenging pathway directly and also through regulating the expression of zinc finger transcription factors (Yang et al. 2009). *OsTF1L*, detected in MQTL_DRI8.1, encodes a homeodomain-leucine zipper transcription factor. *OsTF1L* plays a key role in regulating drought tolerance mechanisms in rice. Up-regulation of drought-inducible genes and the genes involved in stomatal movement and lignin biosynthesis was observed in plants overexpressing *OsTF1L.* Under drought stress, rice plants overexpressing *OsTF1L* showed enhanced effective photosynthesis, reduced water loss rate and increased drought tolerance at the vegetative stage. Furthermore, enhanced drought tolerance together with increased GY was observed in the *OsTF1L* overexpressing plants than in non-transgenic plants at the reproductive stage (Bang et al. 2019).

Out of the 24 identified MQTLs for DRI trait, four MQTLs overlapped with 19 SNP peak positions discovered through GWAS studies (Fig. 5, Additional file 10: Table S7). *Os03g0288800* and *Os03g0286500* were identified as two novel candidate genes for DRI in rice that was detected in SNP peak positions overlapped with QTL-overview peaks.

### MQTLs and Candidate Genes for Flag Leaf Size

Meta-analysis of QTLs resulted in the identification of 10 MQTLs for FLZ in rice. The most number of MQTLs per chromosome was three MQTLs on chromosome3, followed by two MQTLs on each of chromosomes 2 and 4 (Table 3). MQTL_FLZ1.1 was identified as the most stable MQTL for FLZ having the most number of original QTLs from four independent studies.

Within the MQTL_FLZ1.1 interval, *OsFBK1* (*ORYZA SATIVA F-BOX KELCH 1*) was detected, which encodes an E3 ligase subunit. It has been demonstrated that EP3 and *OsFBK1*, both are functional orthologues of Arabidopsis F-box protein HAWAIIAN SKIRT, influence on plant architecture, organ size, number and size of floral organ, floral morphology, pollen viability, grain size, and weight and affect transcript accumulation of microRNA pathway genes and their targets (Borah and Khurana 2018). The most important mechanism engaged in environmental responses and developmental processes in plants is mitogen-activated protein kinase (MAPK) cascade. *OsMAPK2,* located in MQTL_FLZ3.1 interval, encodes *Oryza sativa MAP kinase 2* gene*. OsMAPK2* may be involved in the stress-signaling pathway and panicle development in rice. *OsMAPK2* plays a role in plant tolerance to various biotic/abiotic stresses based on previous studies. Hur and Kim (2014) indicated that overexpression of *OsMAPK2* affected root development and led to increased tolerance to phosphate deficiency in rice and Arabidopsis (Hur and Kim 2014). The other discovered gene is a cytokinin receptor, called *OsHk6 (OsCKT1*), detected in the MQTL_BY2.2 interval. *OsHk6* is involved in cytokinin regulation of biological processes like secondary metabolism, sucrose and starch metabolism, chlorophyll synthesis and photosynthesis (Choi et al. 2012).

### MQTLs and Candidate Genes for Harvest Index

Meta-analysis of QTLs led to the identification of 10 MQTLs for HI. The highest number of MQTLs per chromosome were three MQTLs on chromosome 6, followed by 2 MQTLs on each of the chromosomes 1, 2, and 3 (Table 3). MQTL_HI3.2 having the most number of original QTLs from 12 independent studies and MQTL_HI2.2 having CI < 1 cM was recognized as the most stable MQTLs for the HI under drought stress (Table 3). Out of the 10 detected MQTLs for HI trait, one MQTL overlapped with SNP peak positions discovered in GWAS studies (Fig. 5, Additional file 10: Table S7).

*OsNAC6, OsRPK1, OsZFP, OsCOI1a, OsPP15, OsKASI, OsETOL1* and *OsMSRMK2* detected in the intervals of MQTL_HI1.2, MQTL_HI1.1, MQTL_HI1.2, MQTL_HI1.2, MQTL_HI1.2, MQTL_HI6.2, MQTL_HI3.2 and MQTL_HI3.2, respectively, were recognized as potential candidate genes for HI under drought stress conditions.

The transcription factor of *OsNAC6* up-regulates the expression of the genes involved in several drought tolerance pathways such as genes engaged in membrane modification, nicotianamine biosynthesis, glutathione translocation, 3′-phophoadenosine 5′-phosphosulfate accumulation and glycosylation. Altogether, molecular drought tolerance mechanisms are arranged by *OsNAC6*, indicating its potential to be used for developing high-yielding crops under drought stress conditions (Lee et al. 2017). The *OsRPK1* gene, encoding a Ca^2+^-independent Ser/Thr kinase, was induced by auxin, ABA, cold and drought stresses. Knockdown of *OsRPK1* led to enhanced growth, plant height and tiller number in transgenic rice plants. Furthermore, polar auxin transport and development of root are negatively regulated by *OsRPK1* in rice (Zou et al. 2014)*. OsZFP* encodes a C2HC-type zinc finger protein that plays a role in regulating the development of lateral roots through IAA pathways (Cui et al. 2017). The F-box protein *OsCOI1* is involved in drought tolerance in rice through participating in the signaling module of *OsbHLH148-OsJAZ-OsCOI1* (Seo et al. 2011). *OsPP15 (OsPP2C09*), encoding a clade A type 2C protein phosphatase, had a positive effect on plant growth but negatively regulated drought tolerance via ABA signaling. On the other hand, *OsPP2C09* interacts with DREB TFs and activates DRE-containing promoters. So, drought response regulon is positively regulated by *OsPP2C09*, leading to the activation of an ABA-independent signaling pathway. Altogether, *OsPP2C09* is involved in both ABA-dependent and independent abiotic stress signaling pathways as a bifunctional regulator (Chen et al. 2014; Min et al. 2021). *OsKASI* encodes β-ketoacyl-[acyl carrier protein] synthase I. *OsKASI* deficiency led to decreased fertility and a considerable change in the composition and contents of fatty acids in roots and seeds. It was demonstrated that the involvement of *OsKASI* in fatty acid synthesis is of great importance for rice root development (Ding et al. 2015). *OsETOL1* codes for a homolog of ETHYLENE OVERPRODUCER. Two allelic mutants of *OsETOL1* indicated enhanced tolerance to drought stress during panicle development stage (Du et al. 2014). Diverse biotic/abiotic stresses resulted in changes in the expression of *OsMSRMK2,* indicating its role in defense/stress response pathways of rice (Agrawal et al. 2002).

### MQTLs and Candidate Genes for Panicle Number

We identified 25 MQTLs for the PN trait using MQTL analysis. The highest number of MQTLs per chromosome was four MQTLs on each of the chromosomes 2 and 4 (Table 3). MQTL_PN4.4 was recognized as the most stable MQTL for PN having the highest number of original QTLs from 11 independent studies.

*hbd2*, *IAA6*, *OsAHL1*, *OsC3H10*, *OsCNX*, *OsMOGS*, *OsTSD2, SAPK2, OsCKX9*, *SRS1/DEP2, OsLHT1* and *SAPK2* located in the intervals of MQTL_PN2.3, MQTL_PN1.2, MQTL_PN8.2, MQTL_PN1.2, MQTL_PN4.2, MQTL_PN1.3, MQTL_PN2.4, MQTL_PN7.1, MQTL_PN5.2, MQTL_PN7.1, MQTL_PN8.2 and MQTL_PN7.1, respectively, were detected as potential candidate genes for PN under drought stress conditions.

*OsCKX9,* located in MQTL_PN5.2 interval, encodes cytokinin oxidase 9. Significant enhancements in tiller number and reduction in plant height and panicle size were observed in both *OsCKX9* mutants and *OsCKX9*-overexpressing plants, proposing that *OsCKX9* homeostasis is of great importance for regulating shoot architecture in rice (Duan et al. 2019). *SRS1/DEP2* (The *Small and Round Seed1/*Dense and Erect Panicle2) is engaged in regulating seed size and panicle length in rice (Abe et al. 2010).

*OsLHT1* (MQTL_PN8.2) plays key roles in the translocation of amino acids from vegetative to reproductive organs for GY and quality of nutrition and functionality. The amino acid transporter of *OsLHT1* exhibits a broad substrate specificity and a tendency for neutral and acidic amino acids, and disturbance of *OsLHT1* function noticeably repressed rice growth and fertility. Loss-of-function of *OsLHT1* in two *oslht1* mutants, produced through CRISPR/Cas9 genome-editing technology, led to inhibition of root and shoot growth and significant reduction of grain yield in rice (Guo et al. 2020). *SAPK2* codes for a Serine/threonine-protein kinase and contributes to rice yield by controlling nitrogen metabolic processes under water deficit conditions in the reproductive stage (Lou et al. 2020).

Out of the 25 identified MQTLs for PN trait, 14 MQTLs overlapped with 23 SNP peak positions discovered by GWAS studies (Fig. 5, Additional file 10: Table S7). Three novel candidate genes for PN trait under drought stress conditions in rice include *Os01g0915350 and Os02g0752200, Os02g0752250* (Fig. 5, Additional file 10: Table S7) that were detected in SNP peak positions overlapped with QTL-overview peaks MQTLs.

### MQTLs and Candidate Genes for Spikelet Fertility

We identified 13 MQTLs for SF trait using MQTL analysis. The highest number of MQTLs per chromosome was two MQTLs on each of the chromosomes 4, 5, 6, 8 and 9 (Table 3). MQTL_SF4.2 having the most number of original QTLs from 10 independent studies and MQTL_SF2.1 having CI < 1.17 cM were recognized as the most stable MQTLs for SF trait under drought stress. Out of the 13 identified MQTLs for SF trait, one MQTL overlapped with one SNP peak position detected by GWAS studies (Fig. 5, Additional file 10: Table S7).

Gibberellic acid (GA) plays an important role in development of floral organs and GA signaling has a key function in spikelet fertility. *OsGID1*, detected in MQTL_SF5.1, acts as soluble GA receptor and binds directly to the biologically active GA. Then, OsGID1 interacts with SLR1, a DELLA protein that supress GA signalling. This results in degradation of SLR1 and consequently, permitting GA signaling pathway (Kwon and Paek 2016; Ueguchi-Tanaka et al. 2005). Moreover, *OsMFT1*, detected in MQTL_SF6.2, play an important role in GA biosynthesis and ABA signaling (Lu et al. 2023) and has a main regulatory function under drought stress in rice (Chen et al. 2021). It is also reported that panicle branching and spikelets per panicle in rice is enhanced by *OsMFT1* through suppressing a subfamily of MADS-box genes and SEPALLATA-like genes, respectively (Song et al. 2018).

### Breeding MQTLs

Thirteen MQTLs were identified as "Breeding MQTLs" meeting the following criteria: having more than 10 initial QTLs, a confidence interval (CI) of less than 1 cM, and an average proportion of phenotypic variance explained (PVE) by the original QTLs exceeding 10. These MQTLs are specifically identified as MQTL2-8, MQTL1-12, MQTL8-9, MQTL9-4, MQTL1-5, MQTL12-4, MQTL3-5, MQTL6-3, MQTL1-6, MQTL12-2, MQTL1-11, MQTL3-6, and MQTL8-4 (Table 5, Fig. 3).

## Conclusion

Meta-analysis of yield and drought tolerance associated traits under drought stress conditions led to the discovery of 213 MQTLs, among which 17 MQTLs had a genetic distance of less than 1 cM and accounted for an average phenotypic variance of 20.29%. Notably, 63 MQTLs (out of 213 MQTLs) coincided with SNP positions identified by GWAS for yield components and DT-related traits under drought stress in rice. Moreover, 19 genes precisely situated at the SNP peak positions and QTL-overview peaks were nominated as candidate genes for subsequent functional analysis. These genes were involved in GY (*OsSPO11-5*, *OsDRF1*, *FKF1*, *Os03g0197175*, *Os03g0197200*, *Os03g0305000*, *Os03g0305050*, *Os03g0232800* and *Os08g0170200*), plant height (*Os01g0884500* and *Os01g0885300*), Biomass yield (*OsBRXL2*, *DEK1* (*ADL1*) and *Os06g0164300*), drought response index (*Os03g0288800* and *Os03g0286500*) and panicle number (*Os01g0915350*, *Os02g0752200*, and *Os02g0752250*) under drought stress. On the other hand, the inclusive meta-analysis of QTLs for all the yield-associated traits together led to identification of 13 MQTLs having suitable features to be used as "breeding MQTLs". Finally, integrating the results of MQTL-analysis for yield and DT-associated traits (distinct and combined analysis of traits), GWAS studies, and transcriptome data, resulted in finding 82 candidate genes involved in DT and yield maintenance under drought stress. The promising candidate genes and breeding MQTLs discovered in the current research are valuable sources for genetic engineering and molecular breeding for drought tolerance in rice.

### Supplementary Information

**Additional file 1: Fig. S1.** Venn diagram showing the common genes among the drought responsive genes identified based on the RNA-seq and microarray experiments, and the genes placed within the areas of the 213 identified MQTLs for the distinct traits and the genes placed inside the locations of those MQTLs with CI < 1 cM. Supplementary table S11 includes the detailed information.

**Additional file 2: Fig. S2.** QTL-overview index of yield and DT-associated traits on the consensus genetic map of rice. A total of 1087 initial QTLs from 76 independent studies were used for the analysis. Green and red horizontal lines show the average index (real QTLs) and high-value threshold (QTL hotspot), respectively. The position of the 49 "QTL hotspot" areas are indicated by upper labels. a; QTL-overview index for all the studied traits, b; QTL-overview index for BY, c; QTL-overview index for CP, d; QTL-overview index for DRI, e; QTL-overview index for FLZ, f; QTL-overview index for GY, g; QTL-overview index for HD, h; QTL-overview index for HI, i; QTL-overview index for PH, j; QTL-overview index for PN, k; QTL-overview index for SF.

**Additional file 3: Fig. S3.** The Venn diagram showing the common genes among the genes located in significant SNPs based on GWAS studies, the DEGs (based on RNA-seq and microarray experiments), and the genes placed within the areas of the 213 identified MQTLs for the distinct traits and the genes placed inside the locations of those MQTLs with CI < 1 cM. Supplementary table S12 includes the detailed information.

**Additional file 4: Table S1.** Classification of the studied traits.

**Additional file 5: Table S2.** The high-density consensus genetic map comprises 6970 markers.

**Additional file 6: Table S3.** The genes located in the detected MQTLs regions for each distinct trait.

**Additional file 7: Supplementary Table S4.** The genes located in the detected MQTLs regions through inclusive MQTL analysis.

**Additional file 8: Supplementary Table S5.** The list of RNA-seq and microarray studies used to identify drought-responsive genes in rice.

**Additional file 9: Supplementary Table S6.** Drought responsive genes located in the identified MQTL regions.

**Additional file 10: Supplementary Table S7.** The MQTLs overlapped with the significant SNPs in reported rice GWAS studies for yield and DT-associated traits under drought stress conditions in rice.

**Additional file 11: Supplementary Table S8.** The collected QTL data for performing meta-analysis of QTLs in the current research.

**Additional file 12: Supplementary Table S9.** The identified MQTLs that were associated with more than one trait.

**Additional file 13: Supplementary Table S10.** The drought responsive genes identified in rice based on the related RNA-seq and microarray experiments (Supplemental Table 5).

**Additional file 14: Supplementary Table S11.** The common genes between the drought responsive genes (based on the RNA-seq and microarray experiments) and the genes located in the areas of the 213 identified MQTLs for distinct traits and the genes placed inside the locations of those MQTLs with CI < 1 cM together with their expression levels.

**Additional file 15: Supplementary Table S12.** The common genes among the DEGs (based on RNA-seq and microarray experiments), the genes located in significant SNPs based on GWAS studies, the genes placed within the areas of the 213 identified MQTLs for the distinct traits, and the genes placed inside the locations of those MQTLs with CI < 1 cM together with their expression levels.

**Additional file 16: Supplementary Table S13.** Common genes among the DEGs (based on the related RNA-seq and microarray experiments), the genes located in the MQTL regions detected by inclusive MQTL analysis (96 MQTL), the genes placed within the MQTL areas identified for the distinct traits (213 MQTLs) and the genes located in significant SNPs based on GWAS studies.

## Data Availability

The article and its supplementary include all the supporting data for the current study.

## Abbreviations

QTLs: Quantitative trait loci
GWAS: Genome-wide association studies
LOD: Logarithm of the odds
DT: Drought tolerance
GY: Grain yield
CI: Confidence interval
YLD: Yield-associated traits
GW: Grain weight
HD: Heading date
PH: Plant height
TN: Tiller number

## References

[CR1] Abdirad S, Ghaffari MR, Majd A, Irian S, Soleymaniniya A, Daryani P, Koobaz P, Shobbar Z-S, Farsad LK, Yazdanpanah P (2022). Genome-wide expression analysis of root tips in contrasting rice genotypes revealed novel candidate genes for water stress adaptation. Front Plant Sci.

[CR2] Abe Y, Mieda K, Ando T, Kono I, Yano M, Kitano H, Iwasaki Y (2010). The SMALL AND ROUND SEED1 (SRS1/DEP2) gene is involved in the regulation of seed size in rice. Genes Genet Syst.

[CR3] Agrawal GK, Rakwal R, Iwahashi H (2002). Isolation of novel rice (*Oryza sativa* L.) multiple stress responsive MAP kinase gene, OsMSRMK2, whose mRNA accumulates rapidly in response to environmental cues. Biochem Biophys Res Commun.

[CR4] Arcade A, Labourdette A, Falque M, Mangin B, Chardon F, Charcosset A, Joets J (2004). BioMercator: integrating genetic maps and QTL towards discovery of candidate genes. Bioinformatics.

[CR5] Babu RC, Nguyen BD, Chamarerk V, Shanmugasundaram P, Chezhian P, Jeyaprakash P, Ganesh S, Palchamy A, Sadasivam S, Sarkarung S (2003). Genetic analysis of drought resistance in rice by molecular markers: association between secondary traits and field performance. Crop Sci.

[CR6] Baghyalakshmi K, Jeyaprakash P, Ramchander S, Raveendran M, Robin S (2016). Fine mapping of rice drought QTL and study on combined effect of QTL for their physiological parameters under moisture stress condition. J Appl Nat Sci.

[CR7] Baisakh N, Yabes J, Gutierrez A, Mangu V, Ma P, Famoso A, Pereira A (2020). Genetic mapping identifies consistent quantitative trait loci for yield traits of rice under greenhouse drought conditions. Genes.

[CR8] Bang SW, Lee DK, Jung H, Chung PJ, Kim YS, Choi YD, Suh JW, Kim JK (2019). Overexpression of OsTF1L, a rice HD-Zip transcription factor, promotes lignin biosynthesis and stomatal closure that improves drought tolerance. Plant Biotechnol J.

[CR9] Barik SR, Pandit E, Pradhan SK, Mohanty SP, Mohapatra T (2019). Genetic mapping of morpho-physiological traits involved during reproductive stage drought tolerance in rice. PLoS ONE.

[CR10] Bernier J, Kumar A, Ramaiah V, Spaner D, Atlin G (2007). A large-effect QTL for grain yield under reproductive-stage drought stress in upland rice. Crop Sci.

[CR11] Bhandari A, Sandhu N, Bartholome J, Cao-Hamadoun T-V, Ahmadi N, Kumari N, Kumar A (2020). Genome-wide association study for yield and yield related traits under reproductive stage drought in a diverse indica-aus rice panel. Rice.

[CR12] Bhattarai U, Subudhi PK (2018). Genetic analysis of yield and agronomic traits under reproductive-stage drought stress in rice using a high-resolution linkage map. Gene.

[CR13] Bilgrami S, Darzi Ramandi H, Farokhzadeh S, Rousseau-Gueutin M, Sobhani Najafabadi A, Ghaderian M, Huang P, Liu L (2023). Meta-analysis of seed weight QTLome using a consensus and highly dense genetic map in *Brassica napus* L. Theor Appl Genet.

[CR14] Bilgrami SS, Ramandi HD, Shariati V, Razavi K, Tavakol E, Fakheri BA, Mahdi Nezhad N, Ghaderian M (2020). Detection of genomic regions associated with tiller number in Iranian bread wheat under different water regimes using genome-wide association study. Sci Rep.

[CR15] Bimpong IK, Serraj R, Chin JH, Ramos J, Mendoza EM, Hernandez JE, Mendioro MS, Brar DS (2011). Identification of QTLs for drought-related traits in alien introgression lines derived from crosses of rice (*Oryza sativa* cv. IR64)× O. glaberrima under lowland moisture stress. J Plant Biol.

[CR16] Blum A (2011). Drought resistance–is it really a complex trait?. Funct Plant Biol.

[CR17] Borah P, Khurana JP (2018). The OsFBK1 E3 ligase subunit affects anther and root secondary cell wall thickenings by mediating turnover of a cinnamoyl-CoA reductase. Plant Physiol.

[CR18] Brambilla V, Fornara F (2013). Molecular control of flowering in response to day length in rice. J Integr Plant Biol.

[CR19] Chakraborty S, ZENG, Z.B. (2011). QTL mapping for days to flowering under drought condition in rice (*Oryza sativa* L.) genome. Notulae Botanicae Horti Agrobotanici Cluj-Napoca.

[CR20] Chardon F, Virlon B, Moreau L, Falque M, Joets J, Decousset L, Murigneux A, Charcosset A (2004). Genetic architecture of flowering time in maize as inferred from quantitative trait loci meta-analysis and synteny conservation with the rice genome. Genetics.

[CR21] Chen X, Wang Y, Lv B, Li J, Luo L, Lu S, Zhang X, Ma H, Ming F (2014). The NAC family transcription factor OsNAP confers abiotic stress response through the ABA pathway. Plant Cell Physiol.

[CR22] Chen Y, Shen J, Zhang L, Qi H, Yang L, Wang H, Wang J, Wang Y, Du H, Tao Z (2021). Nuclear translocation of OsMFT1 that is impeded by OsFTIP1 promotes drought tolerance in rice. Mol Plant.

[CR23] Choi J, Lee J, Kim K, Cho M, Ryu H, An G, Hwang I (2012). Functional identification of OsHk6 as a homotypic cytokinin receptor in rice with preferential affinity for iP. Plant Cell Physiol.

[CR24] Courtois B, McLaren G, Sinha P, Prasad K, Yadav R, Shen L (2000). Mapping QTLs associated with drought avoidance in upland rice. Mol Breed.

[CR25] Courtois B, Audebert A, Dardou A, Roques S, Ghneim-Herrera T, Droc G, Frouin J, Rouan L, Gozé E, Kilian A (2013). Genome-wide association mapping of root traits in a japonica rice panel. PLoS ONE.

[CR26] Cui P, Liu H, Ruan S, Ali B, Gill RA, Ma H, Zheng Z, Zhou W (2017). A zinc finger protein, interacted with cyclophilin, affects root development via IAA pathway in rice. J Integr Plant Biol.

[CR27] Darvasi A, Soller M (1997). A simple method to calculate resolving power and confidence interval of QTL map location. Behav Genet.

[CR28] Darvasi A, Weinreb A, Minke V, Weller J, Soller M (1993). Detecting marker-QTL linkage and estimating QTL gene effect and map location using a saturated genetic map. Genetics.

[CR29] Daryani P, Darzi Ramandi H, Dezhsetan S, Mirdar Mansuri R, Hosseini Salekdeh G, Shobbar Z-S (2022). Pinpointing genomic regions associated with root system architecture in rice through an integrative meta-analysis approach. Theor Appl Genet.

[CR30] de Dorlodot S, Forster B, Pagès L, Price A, Tuberosa R, Draye X (2007). Root system architecture: opportunities and constraints for genetic improvement of crops. Trends Plant Sci.

[CR31] Ding W, Lin L, Zhang B, Xiang X, Wu J, Pan Z, Zhu S (2015). OsKASI, a β-ketoacyl-[acyl carrier protein] synthase I, is involved in root development in rice (*Oryza sativa* L.). Planta.

[CR32] Ding W, Tong H, Zheng W, Ye J, Pan Z, Zhang B, Zhu S (2017). Isolation, characterization and transcriptome analysis of a cytokinin receptor mutant Osckt1 in rice. Front Plant Sci.

[CR33] Ding C, Lin X, Zuo Y, Yu Z, Baerson SR, Pan Z, Zeng R, Song Y (2021). Transcription factor OsbZIP49 controls tiller angle and plant architecture through the induction of indole-3-acetic acid-amido synthetases in rice. Plant J.

[CR34] Dixit S, Swamy B, Vikram P, Ahmed H, Sta Cruz M, Amante M, Atri D, Leung H, Kumar A (2012). Fine mapping of QTLs for rice grain yield under drought reveals sub-QTLs conferring a response to variable drought severities. Theor Appl Genet.

[CR35] Dixit S, Singh A, Sta Cruz MT, Maturan PT, Amante M, Kumar A (2014). Multiple major QTL lead to stable yield performance of rice cultivars across varying drought intensities. BMC Genet.

[CR36] Dixit S, Huang BE, Sta Cruz MT, Maturan PT, Ontoy JCE, Kumar A (2014). QTLs for tolerance of drought and breeding for tolerance of abiotic and biotic stress: an integrated approach. PLoS ONE.

[CR37] Dixit S, Grondin A, Lee C-R, Henry A, Olds T-M, Kumar A (2015). Understanding rice adaptation to varying agro-ecosystems: trait interactions and quantitative trait loci. BMC Genet.

[CR38] Du H, Wu N, Cui F, You L, Li X, Xiong L (2014). A homolog of ETHYLENE OVERPRODUCER, O s ETOL 1, differentially modulates drought and submergence tolerance in rice. Plant J.

[CR39] Duan J, Yu H, Yuan K, Liao Z, Meng X, Jing Y, Liu G, Chu J, Li J (2019). Strigolactone promotes cytokinin degradation through transcriptional activation of CYTOKININ OXIDASE/DEHYDROGENASE 9 in rice. Proc Natl Acad Sci.

[CR40] Fukao T, Xiong L (2013). Genetic mechanisms conferring adaptation to submergence and drought in rice: simple or complex?. Curr Opin Plant Biol.

[CR41] Gelli M, Konda AR, Liu K, Zhang C, Clemente TE, Holding DR, Dweikat IM (2017). Validation of QTL mapping and transcriptome profiling for identification of candidate genes associated with nitrogen stress tolerance in sorghum. BMC Plant Biol.

[CR42] Ghimire KH, Quiatchon LA, Vikram P, Swamy BM, Dixit S, Ahmed H, Hernandez JE, Borromeo TH, Kumar A (2012). Identification and mapping of a QTL (qDTY1. 1) with a consistent effect on grain yield under drought. Field Crop Res.

[CR43] Goffinet B, Gerber S (2000). Quantitative trait loci: a meta-analysis. Genetics.

[CR44] Gu J, Yin X, Struik PC, Stomph TJ, Wang H (2012). Using chromosome introgression lines to map quantitative trait loci for photosynthesis parameters in rice (*Oryza sativa* L.) leaves under drought and well-watered field conditions. J Exp Bot.

[CR45] Gu J, Yin X, Zhang C, Wang H, Struik PC (2014). Linking ecophysiological modelling with quantitative genetics to support marker-assisted crop design for improved yields of rice (*Oryza sativa*) under drought stress. Ann Bot.

[CR46] Guo F, Ding C, Zhou Z, Huang G, Wang X (2018). Effects of combined amendments on crop yield and cadmium uptake in two cadmium contaminated soils under rice-wheat rotation. Ecotoxicol Environ Saf.

[CR47] Guo Z, Yang W, Chang Y, Ma X, Tu H, Xiong F, Jiang N, Feng H, Huang C, Yang P (2018). Genome-wide association studies of image traits reveal genetic architecture of drought resistance in rice. Mol Plant.

[CR48] Guo N, Gu M, Hu J, Qu H, Xu G (2020). Rice OsLHT1 functions in leaf-to-panicle nitrogen allocation for grain yield and quality. Front Plant Sci.

[CR49] Heberle H, Meirelles GV, da Silva FR, Telles GP, Minghim R (2015). InteractiVenn: a web-based tool for the analysis of sets through Venn diagrams. BMC Bioinf.

[CR50] Hemamalini G, Shashidhar H, Hittalmani S (2000). Molecular marker assisted tagging of morphological and physiological traits under two contrasting moisture regimes at peak vegetative stage in rice (*Oryza sativa* L.). Euphytica.

[CR51] Ho S-L, Huang L-F, Lu C-A, He S-L, Wang C-C, Yu S-P, Chen J, Yu S-M (2013). Sugar starvation-and GA-inducible calcium-dependent protein kinase 1 feedback regulates GA biosynthesis and activates a 14-3-3 protein to confer drought tolerance in rice seedlings. Plant Mol Biol.

[CR52] Hu H, Xiong L (2014). Genetic engineering and breeding of drought-resistant crops. Annu Rev Plant Biol.

[CR53] Hu SP, Zhou Y, Zhang L, Zhu XD, Li L, Luo LJ, Liu GL, Zhou QM (2009). Correlation and quantitative trait loci analyses of total chlorophyll content and photosynthetic rate of rice (*Oryza sativa*) under water stress and well-watered conditions. J Integr Plant Biol.

[CR54] Hu J, Wang Y, Fang Y, Zeng L, Xu J, Yu H, Shi Z, Pan J, Zhang D, Kang S (2015). A rare allele of GS2 enhances grain size and grain yield in rice. Mol Plant.

[CR55] Hur YJ, Kim DH (2014). Overexpression of OsMAPK2 enhances low phosphate tolerance in rice and Arabidopsis thaliana. Am J Plant Sci.

[CR56] James D, Borphukan B, Fartyal D, Ram B, Singh J, Manna M, Sheri V, Panditi V, Yadav R, Achary VMM (2018). Concurrent overexpression of OsGS1; 1 and OsGS2 genes in transgenic rice (*Oryza sativa* L.): impact on tolerance to abiotic stresses. Front Plant Sci.

[CR57] Kadam NN, Struik PC, Rebolledo MC, Yin X, Jagadish SK (2018). Genome-wide association reveals novel genomic loci controlling rice grain yield and its component traits under water-deficit stress during the reproductive stage. J Exp Bot.

[CR58] Kato Y, Hirotsu S, Nemoto K, Yamagishi J (2008). Identification of QTLs controlling rice drought tolerance at seedling stage in hydroponic culture. Euphytica.

[CR59] Kawahara Y, de la Bastide M, Hamilton JP, Kanamori H, McCombie WR, Ouyang S, Schwartz DC, Tanaka T, Wu J, Zhou S (2013). Improvement of the *Oryza sativa* Nipponbare reference genome using next generation sequence and optical map data. Rice.

[CR60] Khahani B, Tavakol E, Shariati V, Rossini L (2021). Meta-QTL and ortho-MQTL analyses identified genomic regions controlling rice yield, yield-related traits and root architecture under water deficit conditions. Sci Rep.

[CR61] Khowaja FS, Price AH (2008). QTL mapping rolling, stomatal conductance and dimension traits of excised leaves in the Bala× Azucena recombinant inbred population of rice. Field Crop Res.

[CR62] Khowaja FS, Norton GJ, Courtois B, Price AH (2009). Improved resolution in the position of drought-related QTLs in a single mapping population of rice by meta-analysis. BMC Genom.

[CR63] Kim T-H, Hur Y-J, Han S-I, Cho J-H, Kim K-M, Lee J-H, Song Y-C, Kwon Y-U, Shin D (2017). Drought-tolerant QTL qVDT11 leads to stable tiller formation under drought stress conditions in rice. Plant Sci.

[CR64] Krzywinski M, Schein J, Birol I, Connors J, Gascoyne R, Horsman D, Jones SJ, Marra MA (2009). Circos: an information aesthetic for comparative genomics. Genome Res.

[CR65] Kumar R, Venuprasad R, Atlin G (2007). Genetic analysis of rainfed lowland rice drought tolerance under naturally-occurring stress in eastern India: heritability and QTL effects. Field Crop Res.

[CR66] Kwon C-T, Paek N-C (2016). Gibberellic acid: a key phytohormone for spikelet fertility in rice grain production. Int J Mol Sci.

[CR67] Lafitte H, Price AH, Courtois B (2004). Yield response to water deficit in an upland rice mapping population: associations among traits and genetic markers. Theor Appl Genet.

[CR68] Lanceras JC, Pantuwan G, Jongdee B, Toojinda T (2004). Quantitative trait loci associated with drought tolerance at reproductive stage in rice. Plant Physiol.

[CR69] Lang N, Nha C, Ha P, Buu B (2013). Quantitative trait loci (QTLs) associated with drought tolerance in rice (*Oryza sativa* L.). Sabrao J Breed Genet.

[CR70] Lee DK, Chung PJ, Jeong JS, Jang G, Bang SW, Jung H, Kim YS, Ha SH, Choi YD, Kim JK (2017). The rice Os NAC 6 transcription factor orchestrates multiple molecular mechanisms involving root structural adaptions and nicotianamine biosynthesis for drought tolerance. Plant Biotechnol J.

[CR71] Li S, Gao F, Xie K, Zeng X, Cao Y, Zeng J, He Z, Ren Y, Li W, Deng Q (2016). The OsmiR396c-OsGRF4-OsGIF1 regulatory module determines grain size and yield in rice. Plant Biotechnol J.

[CR72] Liang S, Wu L, Ren G, Zhao X, Zhou M, McNeil D, Ye G (2016). Genome-wide association study of grain yield and related traits using a collection of advanced indica rice breeding lines for irrigated ecosystems. Field Crop Res.

[CR73] Lin M-H, Lin C-W, Chen J-C, Lin Y-C, Cheng S-Y, Liu T-H, Jan F-J, Wu S-T, Thseng F-S, Ku H-M (2007) Tagging rice drought-related QTL with SSR DNA markers. 作物, 環境與生物資訊 4:65–76.

[CR74] Liu H, Zou G, Liu G, Hu S, Li M, Yu X, Mei H, Luo L (2005). Correlation analysis and QTL identification for canopy temperature, leaf water potential and spikelet fertility in rice under contrasting moisture regimes. Chin Sci Bull.

[CR75] Liu G, Mei H, Yu X, Zou G, Liu H, Hu S, Li M, Wu J, Chen L, Luo L (2008). QTL analysis of panicle neck diameter, a trait highly correlated with panicle size, under well-watered and drought conditions in rice (*Oryza sativa* L.). Plant Sci.

[CR76] Löffler M, Schön C-C, Miedaner T (2009). Revealing the genetic architecture of FHB resistance in hexaploid wheat (*Triticum aestivum* L.) by QTL meta-analysis. Mol Breeding.

[CR77] Loni F, Ismaili A, Nakhoda B, Ramandi HD, Shobbar Z-S (2023) The genomic regions and candidate genes associated with drought tolerance and yield-related traits in foxtail millet: an integrative meta-analysis approach

[CR78] Lou D, Chen Z, Yu D, Yang X (2020). SAPK2 contributes to rice yield by modulating nitrogen metabolic processes under reproductive stage drought stress. Rice.

[CR79] Lu K, Guo Z, Di S, Lu Y, Muhammad IAR, Rong C, Ding Y, Li W, Ding C (2023). OsMFT1 inhibits seed germination by modulating abscisic acid signaling and gibberellin biosynthesis under salt stress in rice. Plant Cell Physiol.

[CR80] Ma X, Feng F, Wei H, Mei H, Xu K, Chen S, Li T, Liang X, Liu H, Luo L (2016). Genome-wide association study for plant height and grain yield in rice under contrasting moisture regimes. Front Plant Sci.

[CR81] Ma Y, Xue H, Zhang L, Zhang F, Ou C, Wang F, Zhang Z (2016). Involvement of auxin and brassinosteroid in dwarfism of autotetraploid apple (Malus× domestica). Sci Rep.

[CR82] Ma Y, Yang C, He Y, Tian Z, Li J, Sunkar R (2017). Rice OVATE family protein 6 regulates plant development and confers resistance to drought and cold stresses. J Exp Bot.

[CR83] Mardani Z, Rabiei B, Sabouri H, Sabouri A (2013). Mapping of QTLs for germination characteristics under non-stress and drought stress in rice. Rice Sci.

[CR84] Matsubara K, Yamamoto E, Kobayashi N, Ishii T, Tanaka J, Tsunematsu H, Yoshinaga S, Matsumura O, Yonemaru J-i, Mizobuchi R (2016). Improvement of rice biomass yield through QTL-based selection. PLoS ONE.

[CR85] Melandri G, Prashar A, McCouch SR, Van Der Linden G, Jones HG, Kadam N, Jagadish K, Bouwmeester H, Ruyter-Spira C (2020). Association mapping and genetic dissection of drought-induced canopy temperature differences in rice. J Exp Bot.

[CR86] Michael Gomez S, Manikanda Boopathi N, Satheesh Kumar S, Ramasubramanian T, Chengsong Z, Jeyaprakash P, Senthil A, Chandra Babu R (2010). Molecular mapping and location of QTLs for drought-resistance traits in indica rice (*Oryza sativa* L.) lines adapted to target environments. Acta Physiol Plant.

[CR87] Min MK, Kim R, Hong W-J, Jung K-H, Lee J-Y, Kim B-G (2021). OsPP2C09 is a bifunctional regulator in both ABA-dependent and independent abiotic stress signaling pathways. Int J Mol Sci.

[CR88] Mishra KK, Vikram P, Yadaw RB, Swamy B, Dixit S, Cruz MTS, Maturan P, Marker S, Kumar A (2013). qDTY12. 1: a locus with a consistent effect on grain yield under drought in rice. BMC Genet.

[CR89] Nie YY, Zhang L, Wu YH, Liu HJ, Mao WW, Du J, Xiu HL, Wu XY, Li X, Yan YW (2015). Retracted: Screening of candidate genes and fine mapping of drought tolerance quantitative trait loci on chromosome 4 in rice (*Oryza sativa* L.) under drought stress. Ecol Evol.

[CR90] Ning Y, Jantasuriyarat C, Zhao Q, Zhang H, Chen S, Liu J, Liu L, Tang S, Park CH, Wang X (2011). The SINA E3 ligase OsDIS1 negatively regulates drought response in rice. Plant Physiol.

[CR91] Palanog AD, Swamy BM, Shamsudin NAA, Dixit S, Hernandez JE, Boromeo TH, Cruz PCS, Kumar A (2014). Grain yield QTLs with consistent-effect under reproductive-stage drought stress in rice. Field Crop Res.

[CR92] Pantaliao GF, Narciso M, Guimarães C, Castro A, Colombari JM, Breseghello F, Rodrigues L, Vianello RP, Borba TO, Brondani C (2016). Genome wide association study (GWAS) for grain yield in rice cultivated under water deficit. Genetica.

[CR93] Pariasca-Tanaka J, Baertschi C, Wissuwa M (2020). Identification of loci through genome-wide association studies to improve tolerance to sulfur deficiency in rice. Front Plant Sci.

[CR94] Park J-R, Resolus D, Kim K-M (2021). Osbrkq1, related grain size mapping, and identification of grain shape based on qtl mapping in rice. Int J Mol Sci.

[CR95] Petersson SV, Johansson AI, Kowalczyk M, Makoveychuk A, Wang JY, Moritz T, Grebe M, Benfey PN, Sandberg G, Ljung K (2009). An auxin gradient and maximum in the Arabidopsis root apex shown by high-resolution cell-specific analysis of IAA distribution and synthesis. Plant Cell.

[CR96] Prince SJ, Beena R, Gomez SM, Senthivel S, Babu RC (2015). Mapping consistent rice (*Oryza sativa* L.) yield QTLs under drought stress in target rainfed environments. Rice.

[CR97] Qun X, Yuan X-P, Yu H-Y, Wang Y-P, Tang S-X, Wei X-H (2011). Mapping QTLs for drought tolerance at seedling stage in rice using doubled haploid population. Rice Sci.

[CR98] Ren D, Cui Y, Hu H, Xu Q, Rao Y, Yu X, Zhang Y, Wang Y, Peng Y, Zeng D (2019). AH 2 encodes a MYB domain protein that determines hull fate and affects grain yield and quality in rice. Plant J.

[CR99] Robin S, Pathan M, Courtois B, Lafitte R, Carandang S, Lanceras S, Amante M, Nguyen HT, Li Z (2003). Mapping osmotic adjustment in an advanced back-cross inbred population of rice. Theor Appl Genet.

[CR100] Sabar M, Shabir G, Shah SM, Aslam K, Naveed SA, Arif M (2019). Identification and mapping of QTLs associated with drought tolerance traits in rice by a cross between Super Basmati and IR55419-04. Breed Sci.

[CR101] Sabouri H, Dadras AR, Sabouri A, Katouzi M (2013). Mapping QTLs for agronomic traits in rice under water stress condition using Iranian recombinant inbred lines population. J Plant Physiol Breed.

[CR102] Saikumar S, Gouda PK, Saiharini A, Varma CMK, Vineesha O, Padmavathi G, Shenoy VV (2014). Major QTL for enhancing rice grain yield under lowland reproductive drought stress identified using an O. sativa/O. glaberrima introgression line. Field Crop Res.

[CR103] Sandhu N, Jain S, Kumar A, Mehla BS, Jain R (2013). Genetic variation, linkage mapping of QTL and correlation studies for yield, root, and agronomic traits for aerobic adaptation. BMC Genet.

[CR104] Sandhu N, Singh A, Dixit S, Sta Cruz MT, Maturan PC, Jain RK, Kumar A (2014). Identification and mapping of stable QTL with main and epistasis effect on rice grain yield under upland drought stress. BMC Genet.

[CR105] Sandhu N, Subedi SR, Singh VK, Sinha P, Kumar S, Singh S, Ghimire SK, Pandey M, Yadaw RB, Varshney RK (2019). Deciphering the genetic basis of root morphology, nutrient uptake, yield, and yield-related traits in rice under dry direct-seeded cultivation systems. Sci Rep.

[CR106] Sangodele E, Hanchinal R, Hanamaratti N, Shenoy V, Kumar V (2014). Analysis of drought tolerant QTL linked to physiological and productivity component traits under water-stress and non-stress in rice (*Oryza sativa* L.). Int J Curr Res Acad Rev.

[CR107] Sellamuthu R, Ranganathan C, Serraj R (2015). Mapping QTLs for reproductive-stage drought resistance traits using an advanced backcross population in upland rice. Crop Sci.

[CR108] Seo JS, Joo J, Kim MJ, Kim YK, Nahm BH, Song SI, Cheong JJ, Lee JS, Kim JK, Choi YD (2011). OsbHLH148, a basic helix-loop-helix protein, interacts with OsJAZ proteins in a jasmonate signaling pathway leading to drought tolerance in rice. Plant J.

[CR109] Shamsudin NAA, Swamy B, Ratnam W, Cruz S, Teressa M, Sandhu N, Raman AK, Kumar A (2016). Pyramiding of drought yield QTLs into a high quality Malaysian rice cultivar MRQ74 improves yield under reproductive stage drought. Rice.

[CR110] Shao-Xia Z, Feng T, Zuo-Feng Z, Yong-Cai F, Xiang-Kun W, Chuan-Qing S (2006). Identification of quantitative trait loci controlling drought tolerance at seedling stage in Chinese Dongxiang common wild rice (*Oryza rufipogon* Griff.). Acta Genet Sin.

[CR111] Shelley IJ, Nishiuchi S, Shibata K, Inukai Y (2013). SLL1, which encodes a member of the stearoyl-acyl carrier protein fatty acid desaturase family, is involved in cell elongation in lateral roots via regulation of fatty acid content in rice. Plant Sci.

[CR112] Singhal A, Tien Y-Y, Hsia RY (2016). Racial-ethnic disparities in opioid prescriptions at emergency department visits for conditions commonly associated with prescription drug abuse. PLoS ONE.

[CR113] Solis J, Gutierrez A, Mangu V, Sanchez E, Bedre R, Linscombe S, Baisakh N (2018). Genetic mapping of quantitative trait loci for grain yield under drought in rice under controlled greenhouse conditions. Front Chem.

[CR114] Song S, Wang G, Hu Y, Liu H, Bai X, Qin R, Xing Y (2018). OsMFT1 increases spikelets per panicle and delays heading date in rice by suppressing Ehd1, FZP and SEPALLATA-like genes. J Exp Bot.

[CR115] Srinivasan S, Gomez SM, Kumar SS, Ganesh S, Biji K, Senthil A, Babu RC (2008). QTLs linked to leaf epicuticular wax, physio-morphological and plant production traits under drought stress in rice (*Oryza sativa* L.). Plant Growth Regul.

[CR116] Su L, Yang J, Li D, Peng Z, Xia A, Yang M, Luo L, Huang C, Wang J, Wang H (2021). Dynamic genome-wide association analysis and identification of candidate genes involved in anaerobic germination tolerance in rice. Rice.

[CR117] Subashri M, Robin S, Vinod K, Rajeswari S, Mohanasundaram K, Raveendran T (2009). Trait identification and QTL validation for reproductive stage drought resistance in rice using selective genotyping of near flowering RILs. Euphytica.

[CR118] Suji K, Biji K, Poornima R, Prince K, Amudha K, Kavitha S, Mankar S, Babu RC (2012). Mapping QTLs for plant phenology and production traits using indica rice (*Oryza sativa* L.) lines adapted to rainfed environment. Mol Biotechnol.

[CR119] Sun X, Ma Y, Yang C, Li J (2020). Rice OVATE family protein 6 regulates leaf angle by modulating secondary cell wall biosynthesis. Plant Mol Biol.

[CR120] Swamy B, Vikram P, Dixit S, Ahmed H, Kumar A (2011). Meta-analysis of grain yield QTL identified during agricultural drought in grasses showed consensus. BMC Genomics.

[CR121] Swamy BPM, Ahmed HU, Henry A, Mauleon R, Dixit S, Vikram P, Tilatto R, Verulkar SB, Perraju P, Mandal NP (2013). Genetic, physiological, and gene expression analyses reveal that multiple QTL enhance yield of rice mega-variety IR64 under drought. PLoS ONE.

[CR122] Swamy B, Shamsudin NAA, Rahman SNA, Mauleon R, Ratnam W, Cruz S, Teressa M, Kumar A (2017). Association mapping of yield and yield-related traits under reproductive stage drought stress in rice (*Oryza sativa* L.). Rice.

[CR123] Swamy BPM, Ahmed HU, Henry A, Mauleon R, Dixit S, Vikram P, Tilatto R, Verulkar SB, Perraju P, Mandal NP (2013) Genetic, physiological, and gene expression analyses reveal that multiple QTL enhance yield of rice mega-variety IR64 under drought. PloS One 8:e62795 10.1371/journal.pone.0062795 PMC3648568 23667521

[CR124] Tabuchi M, Sugiyama K, Ishiyama K, Inoue E, Sato T, Takahashi H, Yamaya T (2005). Severe reduction in growth rate and grain filling of rice mutants lacking OsGS1; 1, a cytosolic glutamine synthetase1; 1. Plant J.

[CR125] Takai T, Fukuta Y, Sugimoto A, Shiraiwa T, Horie T (2006). Mapping of QTLs controlling carbon isotope discrimination in the photosynthetic system using recombinant inbred lines derived from a cross between two different rice (*Oryza sativa* L.) cultivars. Plant Prod Sci.

[CR126] Tester M, Langridge P (2010). Breeding technologies to increase crop production in a changing world. Science.

[CR127] This D, Comstock J, Courtois B, Xu Y, Ahmadi N, Vonhof WM, Fleet C, Setter T, McCouch S (2010). Genetic analysis of water use efficiency in rice (*Oryza sativa* L.) at the leaf level. Rice.

[CR128] Tian F, Li DJ, Fu Q, Zhu ZF, Fu YC, Wang XK, Sun CQ (2006). Construction of introgression lines carrying wild rice (*Oryza rufipogon* Griff.) segments in cultivated rice (*Oryza sativa* L.) background and characterization of introgressed segments associated with yield-related traits. Theor Appl Genet.

[CR129] To HTM, Nguyen HT, Dang NTM, Nguyen NH, Bui TX, Lavarenne J, Phung NTP, Gantet P, Lebrun M, Bellafiore S (2019). Unraveling the genetic elements involved in shoot and root growth regulation by jasmonate in rice using a genome-wide association study. Rice.

[CR130] Trijatmiko KR, Prasetiyono J, Thomson MJ, Vera Cruz CM, Moeljopawiro S, Pereira A (2014). Meta-analysis of quantitative trait loci for grain yield and component traits under reproductive-stage drought stress in an upland rice population. Mol Breed.

[CR131] Ueguchi-Tanaka M, Ashikari M, Nakajima M, Itoh H, Katoh E, Kobayashi M, Chow T-y, Y-iC H, Kitano H, Yamaguchi I (2005). Gibberellin insensitive DWARF1 encodes a soluble receptor for gibberellin. Nature.

[CR132] Venuprasad R, Dalid C, Del Valle M, Zhao D, Espiritu M, Sta Cruz M, Amante M, Kumar A, Atlin G (2009). Identification and characterization of large-effect quantitative trait loci for grain yield under lowland drought stress in rice using bulk-segregant analysis. Theor Appl Genet.

[CR133] Verma SK, Saxena RR, Saxena RR, Xalxo MS, Verulkar SB (2014). QTL for grain yield under water stress and non-stress conditions over years in rice ('*Oryza sativa*'L.). Austral J Crop Sci.

[CR134] Veyrieras J-B, Goffinet B, Charcosset A (2007). MetaQTL: a package of new computational methods for the meta-analysis of QTL mapping experiments. BMC Bioinf.

[CR135] Vikram P, Swamy B, Dixit S, Ahmed HU, Cruz TS, M, Singh AK, Kumar A  (2011). qDTY 1.1, a major QTL for rice grain yield under reproductive-stage drought stress with a consistent effect in multiple elite genetic backgrounds. BMC Genet.

[CR136] Vikram P, Swamy BM, Dixit S, Ahmed H, Cruz MS, Singh AK, Ye G, Kumar A (2012). Bulk segregant analysis: “An effective approach for mapping consistent-effect drought grain yield QTLs in rice”. Field Crop Res.

[CR137] Wang B, Sang Y, Song J, Gao X-Q, Zhang X (2009). Expression of a rice OsARGOS gene in Arabidopsis promotes cell division and expansion and increases organ size. J Genet Genomics.

[CR138] Wang Y, Zang J, Sun Y, Ali J, Xu J, Li Z (2013). Background-independent quantitative trait loci for drought tolerance identified using advanced backcross introgression lines in rice. Crop Sci.

[CR139] Wang L, Yu C, Chen C, He C, Zhu Y, Huang W (2014). Identification of rice Di19 family reveals OsDi19-4 involved in drought resistance. Plant Cell Rep.

[CR140] Wang L, Yu C, Xu S, Zhu Y, Huang W (2016). OsDi19-4 acts downstream of OsCDPK14 to positively regulate ABA response in rice. Plant, Cell Environ.

[CR141] Wang J, Lu K, Nie H, Zeng Q, Wu B, Qian J, Fang Z (2018). Rice nitrate transporter OsNPF7. 2 positively regulates tiller number and grain yield. Rice.

[CR142] Wang L, Cheng Y, Ma Q, Mu Y, Huang Z, Xia Q, Zhang G, Nian H (2019). QTL fine-mapping of soybean (*Glycine max* L.) leaf type associated traits in two RILs populations. BMC Genom.

[CR143] Wang F, Yano K, Nagamatsu S, Inari-Ikeda M, Koketsu E, Hirano K, Aya K, Matsuoka M (2020). Genome-wide expression quantitative trait locus studies facilitate isolation of causal genes controlling panicle structure. Plant J.

[CR144] Wei H, Wang X, Xu H, Wang L (2020) Molecular basis of heading date control in rice. aBIOTECH 1:219–232 10.1007/s42994-020-00019-w PMC9590479 36304129

[CR145] Wu Y, Huang M, Tao X, Guo T, Chen Z, Xiao W (2016). Quantitative trait loci identification and meta-analysis for rice panicle-related traits. Mol Genet Genom.

[CR146] Xing Y, Zhang Q (2010). Genetic and molecular bases of rice yield. Annu Rev Plant Biol.

[CR147] Xing W, Zhao H, Zou D (2014). Detection of main-effect and epistatic QTL for yield-related traits in rice under drought stress and normal conditions. Can J Plant Sci.

[CR148] Xu J, Lafitte H, Gao Y, Fu B, Torres R, Li Z (2005). QTLs for drought escape and tolerance identified in a set of random introgression lines of rice. Theor Appl Genet.

[CR149] Xu K, Chen S, Li T, Ma X, Liang X, Ding X, Liu H, Luo L (2015). OsGRAS23, a rice GRAS transcription factor gene, is involved in drought stress response through regulating expression of stress-responsive genes. BMC Plant Biol.

[CR150] Xu Y, Zhang H, Hu J, Wang X, Huang M, Wang H (2018). Further QTL mapping for yield component traits using introgression lines in rice (*Oryza sativa* L.) under drought field environments. Euphytica.

[CR151] Yadav S, Sandhu N, Singh VK, Catolos M, Kumar A (2019). Genotyping-by-sequencing based QTL mapping for rice grain yield under reproductive stage drought stress tolerance. Sci Rep.

[CR152] Yadaw RB, Dixit S, Raman A, Mishra KK, Vikram P, Swamy BM, Cruz MTS, Maturan PT, Pandey M, Kumar A (2013). A QTL for high grain yield under lowland drought in the background of popular rice variety Sabitri from Nepal. Field Crop Res.

[CR153] Yang Z, Wu Y, Li Y, Ling H-Q, Chu C (2009). OsMT1a, a type 1 metallothionein, plays the pivotal role in zinc homeostasis and drought tolerance in rice. Plant Mol Biol.

[CR154] Yang L, Wang J, Lei L, Wang J, Junaid Subhani M, Liu H, Sun J, Zheng H, Zhao H, Zou D (2018). QTL mapping for heading date, leaf area and chlorophyll content under cold and drought stress in two related recombinant inbred line populations (Japonica rice) and meta-analysis. Plant Breeding.

[CR155] Yang X, Ren Y, Cai Y, Niu M, Feng Z, Jing R, Mou C, Liu X, Xiao L, Zhang X (2018). Overexpression of OsbHLH107, a member of the basic helix-loop-helix transcription factor family, enhances grain size in rice (*Oryza sativa* L.). Rice.

[CR156] Yu Y, Ouyang Y, Yao W (2018). shinyCircos: an R/Shiny application for interactive creation of Circos plot. Bioinformatics.

[CR157] Yue B, Xiong L, Xue W, Xing Y, Luo L, Xu C (2005). Genetic analysis for drought resistance of rice at reproductive stage in field with different types of soil. Theor Appl Genet.

[CR158] Yue B, Xue W, Xiong L, Yu X, Luo L, Cui K, Jin D, Xing Y, Zhang Q (2006). Genetic basis of drought resistance at reproductive stage in rice: separation of drought tolerance from drought avoidance. Genetics.

[CR159] Yue B, Xue W, Luo L, Xing Y (2008). Identification of quantitative trait loci for four morphologic traits under water stress in rice (*Oryza sativa* L.). J Genet Genom.

[CR160] Zhang F, Hu Z, Wu Z, Lu J, Shi Y, Xu J, Wang X, Wang J, Zhang F, Wang M (2021). Reciprocal adaptation of rice and Xanthomonas oryzae pv. oryzae: cross-species 2D GWAS reveals the underlying genetics. Plant Cell.

[CR161] Zhao X-Q, Xu J-L, Zhao M, Lafitte R, Zhu L-H, Fu B-Y, Gao Y-M, Li Z-K (2008). QTLs affecting morph-physiological traits related to drought tolerance detected in overlapping introgression lines of rice (*Oryza sativa* L.). Plant Sci.

[CR162] Zhao J, Wu C, Yuan S, Yin L, Sun W, Zhao Q, Zhao B, Li X (2013). Kinase activity of OsBRI1 is essential for brassinosteroids to regulate rice growth and development. Plant Sci.

[CR163] Zhao L, Tan L, Zhu Z, Xiao L, Xie D, Sun C (2015). PAY 1 improves plant architecture and enhances grain yield in rice. Plant J.

[CR164] Zhao J, Yu N, Ju M, Fan B, Zhang Y, Zhu E, Zhang M, Zhang K (2019). ABC transporter OsABCG18 controls the shootward transport of cytokinins and grain yield in rice. J Exp Bot.

[CR165] Zhou G, Liu F, Cao J, Yue B, Xiong L (2011). Detecting quantitative trait loci for water use efficiency in rice using a recombinant inbred line population. Chin Sci Bull.

[CR166] Zhou S, Zhu M, Wang F, Huang J, Wang G (2013). Mapping of QTLs for yield and its components in a rice recombinant inbred line population. Pak J Bot.

[CR167] Zhou W, Wang X, Zhou D, Ouyang Y, Yao J (2017). Overexpression of the 16-kD a α-amylase/trypsin inhibitor RAG 2 improves grain yield and quality of rice. Plant Biotechnol J.

[CR168] Z-m GU, H-j TANG, X-f CHEN, Feng L, H-s ZHANG (2010). Preliminary study on function of Calcineurin B-like protein gene OsCBL8 in rice. Rice Sci.

[CR169] Zou G, Mei H, Liu H, Liu G, Hu S, Yu X, Li M, Wu J, Luo L (2005). Grain yield responses to moisture regimes in a rice population: association among traits and genetic markers. Theor Appl Genet.

[CR170] Zou Y, Liu X, Wang Q, Chen Y, Liu C, Qiu Y, Zhang W (2014). OsRPK1, a novel leucine-rich repeat receptor-like kinase, negatively regulates polar auxin transport and root development in rice. Biochimica Et Biophysica Acta (BBA)-Gen Subj.

