# Supplementary material for: Uncovering the Genomic Regions Associated with Yield Maintenance in Rice Under Drought Stress Using an Integrated Meta-Analysis Approach
Source: Rice (N Y). 2024 Jan 16;17:7. doi: 10.1186/s12284-024-00684-1 (PMC10792158; doi:10.1186/s12284-024-00684-1)
Supplement: Supplementary file 2 — Additional file 2: Fig. S2. QTL-overview index of yield and DT-associated traits on the consensus genetic map of rice. A total of 1087 initial QTLs from 76 independent studies were used for the analysis. Green and red horizontal lines show the average index (real QTLs) and high-value threshold (QTL hotspot), respectively. The position of the 49 "QTL hotspot" areas are indicated by upper labels. a; QTL-overview index for all the studied traits, b; QTL-overview index for BY, c; QTL-overview index for CP, d; QTL-overview index for DRI, e; QTL-overview index for FLZ, f; QTL-overview index for GY, g; QTL-overview index for HD, h; QTL-overview index for HI, i; QTL-overview index for PH, j; QTL-overview index for PN, k; QTL-overview index for SF. [file 12284_2024_684_MOESM2_ESM.docx]

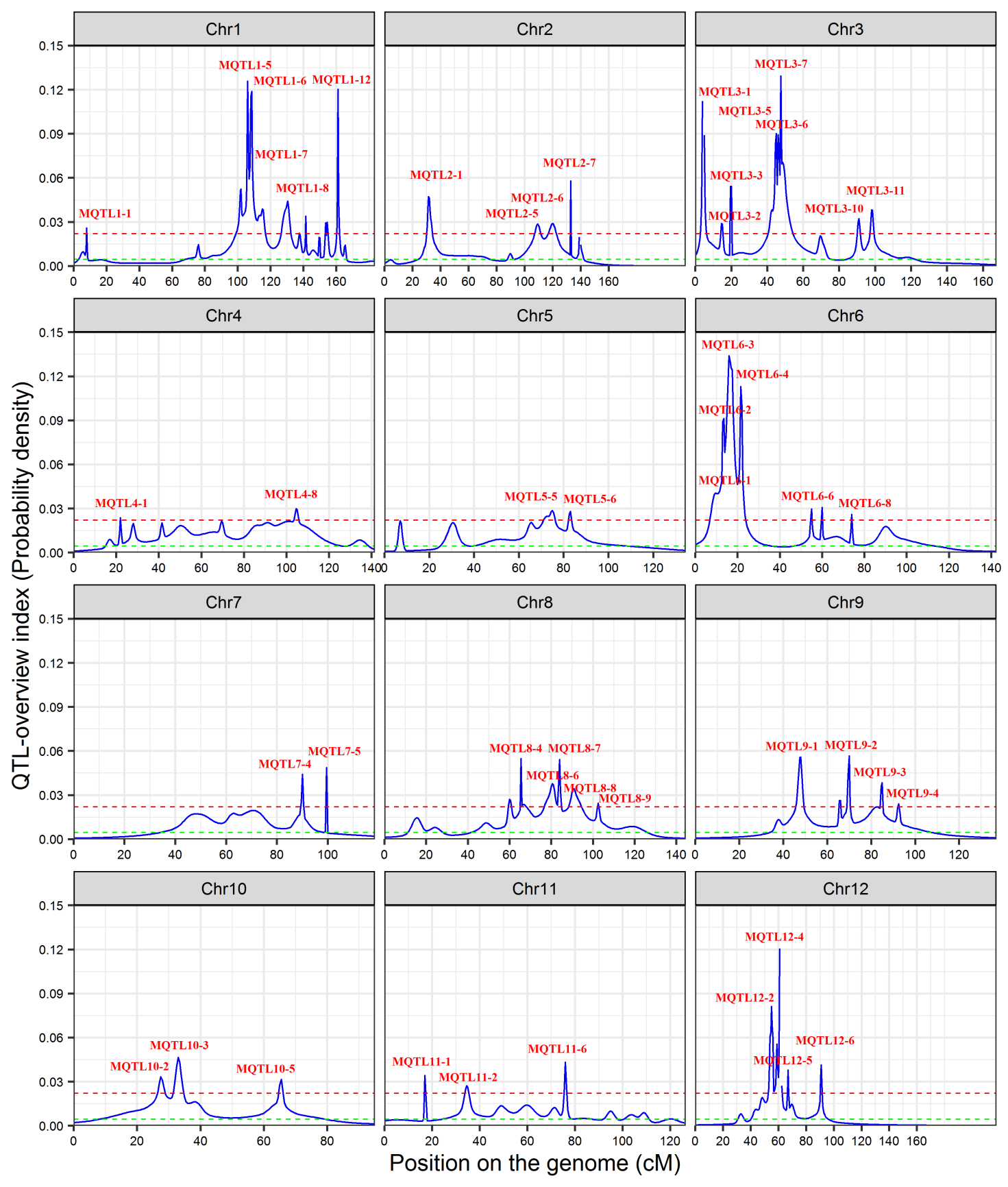


**a**


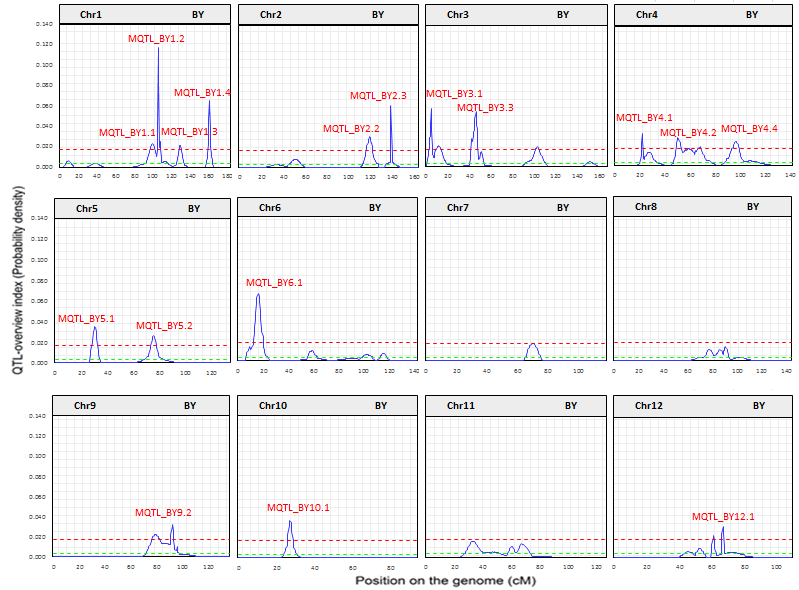


**b**


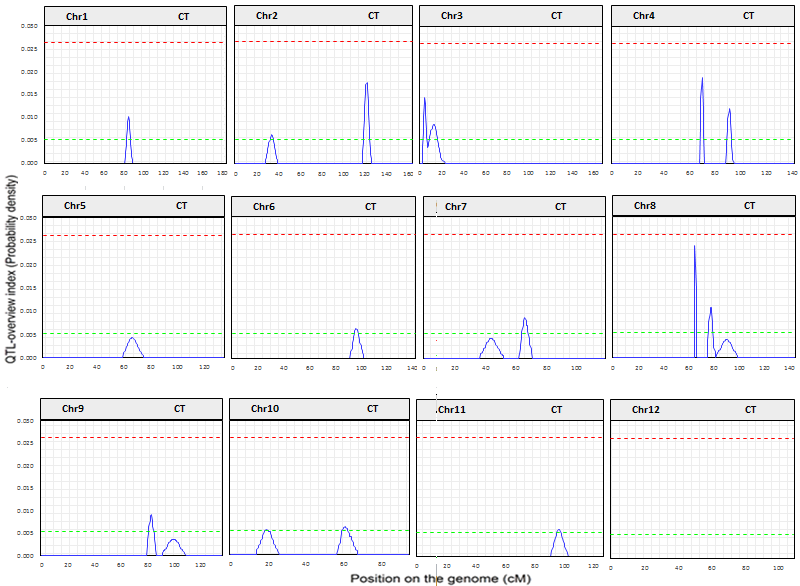


**c**


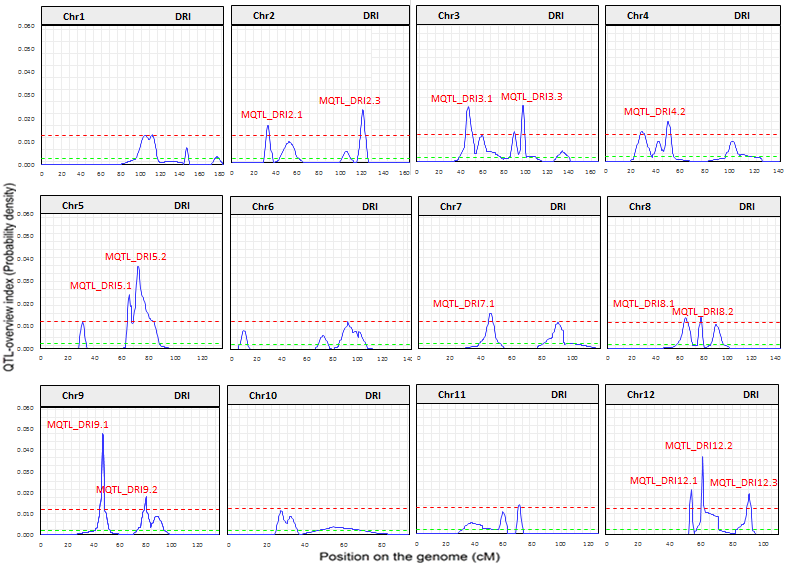


**d**


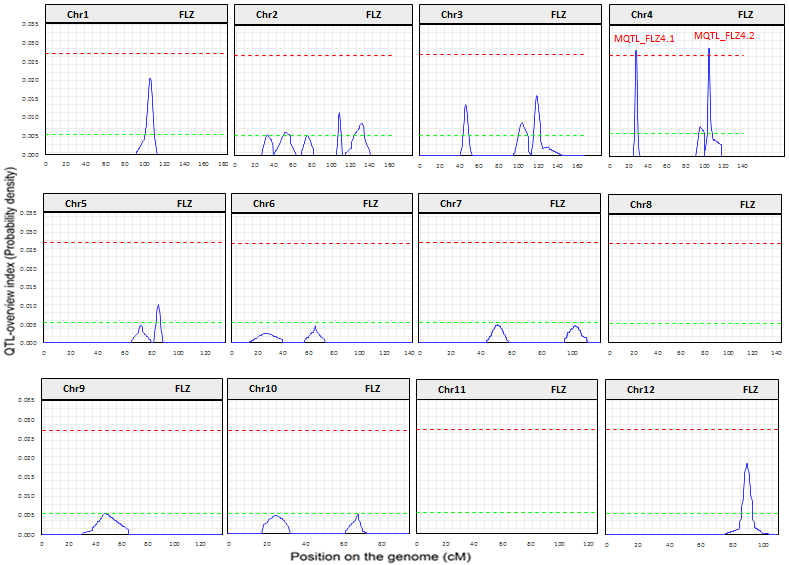


**e**


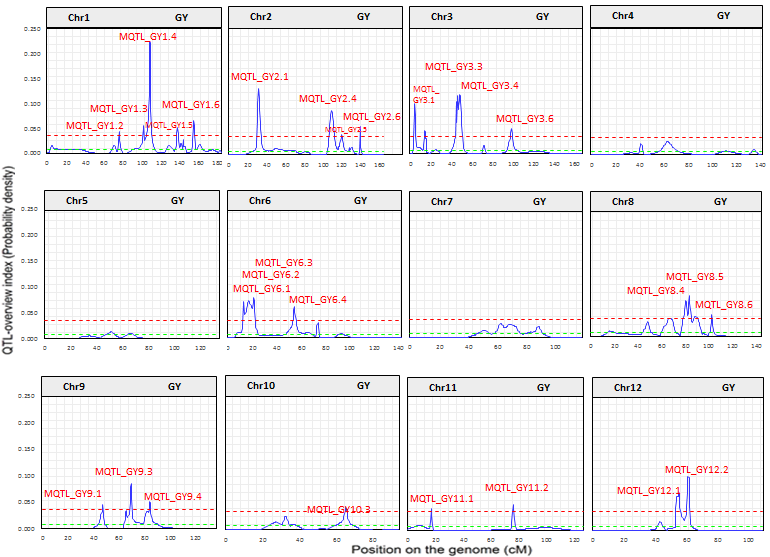


**f**


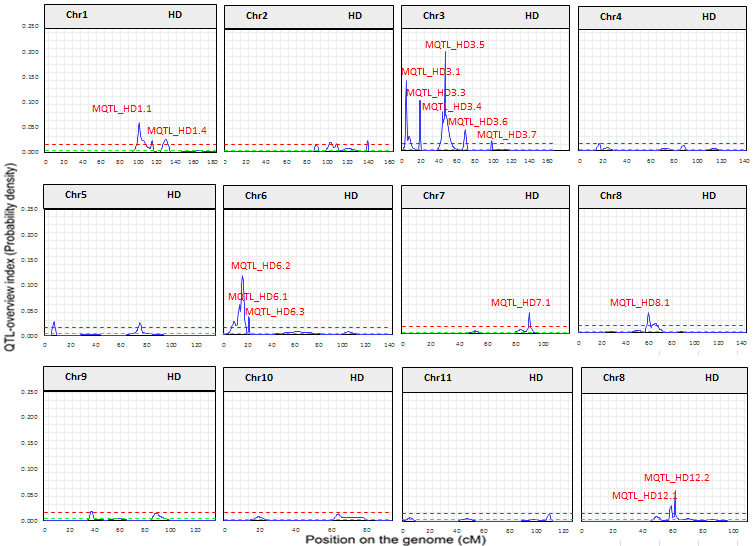


**g**


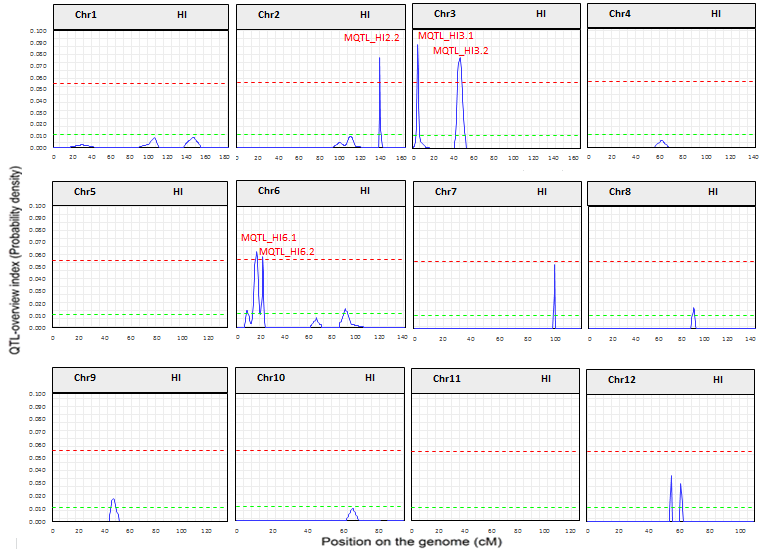


**h**


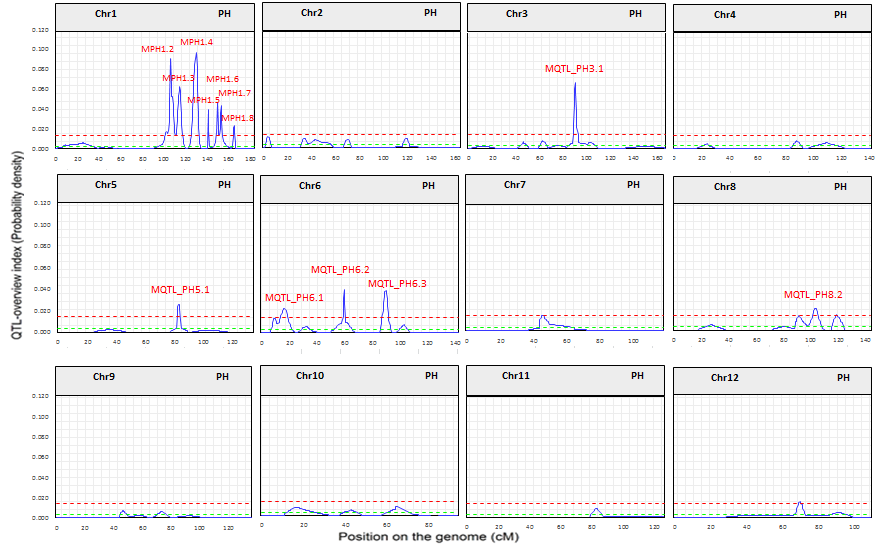


**i**


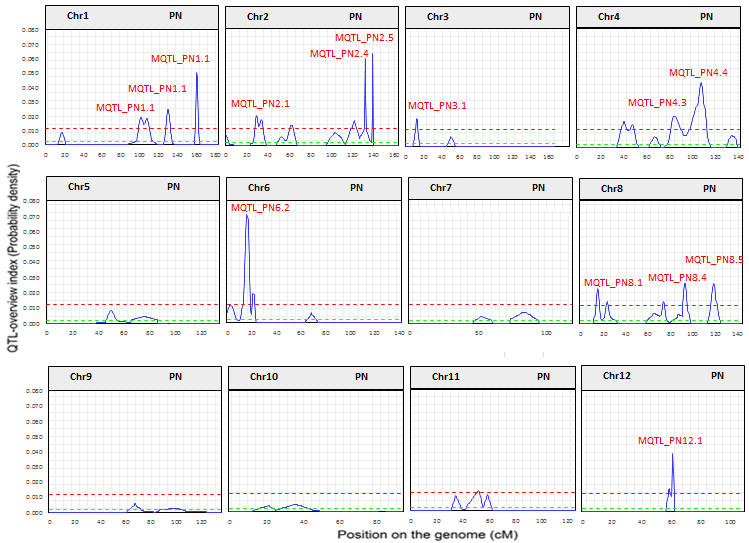


**j**


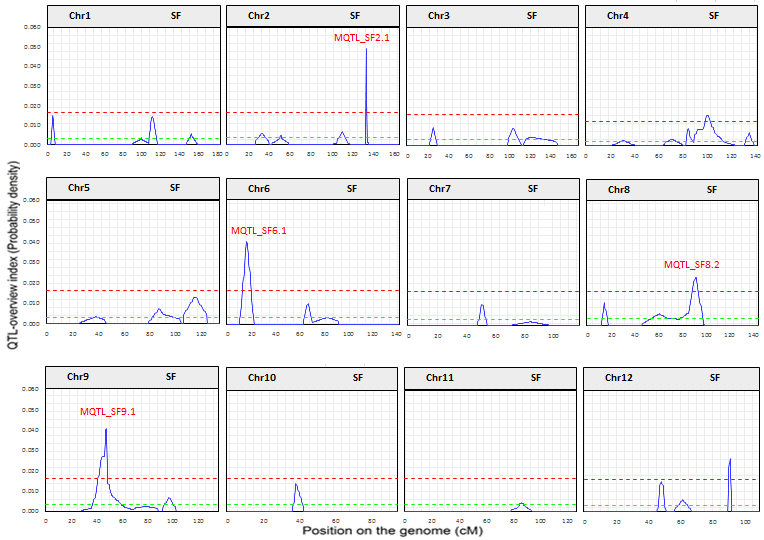


**k**

**Supplementary Figure S2.** QTL-overview index of yield and DT-associated traits on the consensus genetic map of rice. A total of 1087 initial QTLs from 76 independent studies were used for the analysis. Green and red horizontal lines show the average index (real QTLs) and high-value threshold (QTL hotspot), respectively. The position of the 49 "QTL hotspot" areas are indicated by upper labels. a; QTL-overview index for all the studied traits, b; QTL-overview index for BY, c; QTL-overview index for CP, d; QTL-overview index for DRI, e; QTL-overview index for FLZ, f; QTL-overview index for GY, g; QTL-overview index for HD, h; QTL-overview index for HI, i; QTL-overview index for PH, j; QTL-overview index for PN, k; QTL-overview index for SF.

**b**
