# Supplementary material for: Uncovering the Genomic Regions Associated with Yield Maintenance in Rice Under Drought Stress Using an Integrated Meta-Analysis Approach
Source: Rice (N Y). 2024 Jan 16;17:7. doi: 10.1186/s12284-024-00684-1 (PMC10792158; doi:10.1186/s12284-024-00684-1)
Supplement: Supplementary file 3 — Additional file 3: Fig. S3. The Venn diagram showing the common genes among the genes located in significant SNPs based on GWAS studies, the DEGs (based on RNA-seq and microarray experiments), and the genes placed within the areas of the 213 identified MQTLs for the distinct traits and the genes placed inside the locations of those MQTLs with CI < 1 cM. Supplementary table S12 includes the detailed information. [file 12284_2024_684_MOESM3_ESM.docx]

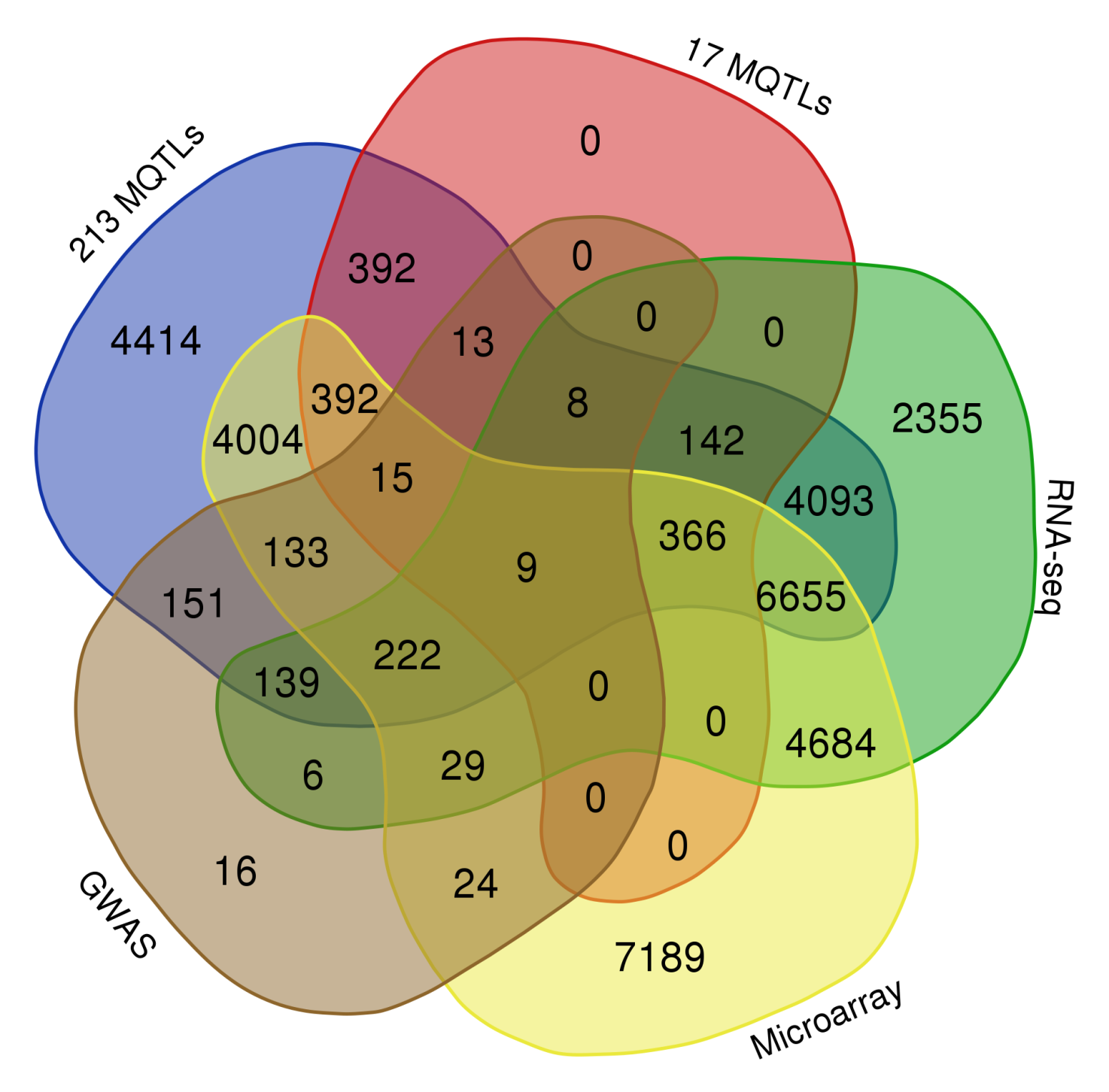


**Supplementary Figure S3.** The Venn diagram showing the common genes among the genes located in significant SNPs based on GWAS studies, the DEGs (based on RNA-seq and microarray experiments) and the genes placed within the areas of the 213 identified MQTLs for the distinct traits and the genes placed inside the areas of those MQTLs with CI < 1cM. Supplementary table S12 includes the detailed information.

**b**
