# Supplementary material for: Uncovering the Genomic Regions Associated with Yield Maintenance in Rice Under Drought Stress Using an Integrated Meta-Analysis Approach
Source: Rice (N Y). 2024 Jan 16;17:7. doi: 10.1186/s12284-024-00684-1 (PMC10792158; doi:10.1186/s12284-024-00684-1)
Supplement: Supplementary file 8 — Additional file 8: Supplementary Table S5. The list of RNA-seq and microarray studies used to identify drought-responsive genes in rice. [file 12284_2024_684_MOESM8_ESM.docx]

**Supplementary Table S5.** The list of RNA-seq and microarray studies used to identify drought-responsive genes in rice.

| **Number** | **References** | **Genotype** | **Developmental stage** | **Tissue** |
| --- | --- | --- | --- | --- |
| 1 | ([Yoo et al., 2017](#_ENREF_20)) | (*Oryza sativa*) cv. Chilbo | Four-week-old seedlings | Leaf, root |
| 2 | ([Ereful et al., 2020](#_ENREF_3)) | IR64, Apo | Early flowering stage | Leaf samples |
| 3 | ([Huang et al., 2014](#_ENREF_5)) | H471, HHZ, P28 | Tillering stage | Fresh leaf |
| 4 | ([Shim et al., 2018](#_ENREF_16)) | *Oryza sativa* cv. Dongjin | Vegetative stage | Higher number of panicle and filling rate |
| 5 | ([Silveira et al., 2015](#_ENREF_17)) | Brazilian rice cultivars | Reproductive phase | Leaf tissues |
| 6 | ([Zhang et al., 2018](#_ENREF_23)) | Nipponbare | Seedling stage | Leaf, diameter of the xylem |
| 7 | ([Zhang et al., 2017](#_ENREF_22)) | *Oryza rufipogon*, DXWR | Seedlings | Leaf, roots with dry treatment (RD) |
| 8 | ([Fu et al., 2017](#_ENREF_4)) | Variety Dongjin (*Oryza sativa* L. ssp. japonica) | Seedlings | Leaf |
| 9 | ([Lee et al., 2017](#_ENREF_8)) | *Oryza sativa* cv Nipponbare | 2-week-old | Tiller number, root length, crown root number, root diameter |
| 10 | ([Yu et al., 2020](#_ENREF_21)) | PY6 , PR403 | During later stages of drought stress | Leaf |
| 11 | ([Chang et al., 2017](#_ENREF_2)) | *Oryza sativa* | Seedlings | Leaf |
| 12 | ([Yang et al., 2019](#_ENREF_19)) |  |  |  |
| 13 | ([Singh et al., 2020](#_ENREF_18)) | ARC-10372 | Leaf at panicle initiation stage | Leaf, panicle |
| 14 | ([Krishnan et al., 2010](#_ENREF_7)) | Nipponbare | seedling, reproductive, vegetative | Plant tissue |
| 15 | ([Sen SK, 2016](#_ENREF_15)) | Nivara, rufipogan, N22 | Vegetative , Grain-filling stage | Leaf |
| 16 | ([Pueffeld, 2016](#_ENREF_13)) | Vandana , Way Rarem, 481-B | Reproductive | Spikelets |
| 17 | ([Pueffeld M, 2016](#_ENREF_14)) | Vandana , Way Rarem, 481-B | Reproductive | Leaves |
| 18 | ([Li et al., 2015](#_ENREF_9)) | N22, Moroberekan | Before and after pollination | Floral organs |
| 19 | ([Ambavaram et al., 2014](#_ENREF_1)) | Nipponbare |  | Tmospheric carbon dioxide (CO2), photosynthetic carbon metabolism (PCM) |
| 20 | ([Moumeni et al., 2011](#_ENREF_10)) | IR77298-14-1-2-B-10, IR77298-5-6-B-18, IR77298-14-1-2-B-13, IR77298-5-6-B-11, IR64 | Reproductive stage | Shoot |
| 21 | ([Ambavaram *et al.*, 2014](#_ENREF_1)) | IR77298-14-1-2-B-10, IR77298-5-6-B-18, IR77298-14-1-2-B-13, IR77298-5-6-B-11, IR65 | Reproductive stage | Panicle |
| 22 | ([Ning et al., 2011](#_ENREF_11)) | *Oryza sativa* (wild type) | Seven-day-old plants | Leaf |
| 23 | ([Huang et al., 2009](#_ENREF_6)) | Zhonghua11 |  | Shoot |
| 24 | ([Peleg et al., 2011](#_ENREF_12)) | Wild-type |  | Flag leaf |

**Ambavaram, M.M., Basu, S., Krishnan, A., Ramegowda, V., Batlang, U., Rahman, L., Baisakh, N., and Pereira, A.** (2014). Coordinated regulation of photosynthesis in rice increases yield and tolerance to environmental stress. Nature communications **5**:1-14.

**Chang, Y., Nguyen, B.H., Xie, Y., Xiao, B., Tang, N., Zhu, W., Mou, T., and Xiong, L.** (2017). Co-overexpression of the constitutively active form of OsbZIP46 and ABA-activated protein kinase SAPK6 improves drought and temperature stress resistance in rice. Frontiers in plant science **8**:1102.

**Ereful, N.C., Liu, L.-y., Greenland, A., Powell, W., Mackay, I., and Leung, H.** (2020). RNA-seq reveals differentially expressed genes between two indica inbred rice genotypes associated with drought-yield QTLs. Agronomy **10**:621.

**Fu, J., Wu, H., Ma, S., Xiang, D., Liu, R., and Xiong, L.** (2017). OsJAZ1 attenuates drought resistance by regulating JA and ABA signaling in rice. Frontiers in plant science **8**:2108.

**Huang, L., Zhang, F., Wang, W., Zhou, Y., Fu, B., and Li, Z.** (2014). Comparative transcriptome sequencing of tolerant rice introgression line and its parents in response to drought stress. BMC genomics **15**:1-16.

**Huang, X.-Y., Chao, D.-Y., Gao, J.-P., Zhu, M.-Z., Shi, M., and Lin, H.-X.** (2009). A previously unknown zinc finger protein, DST, regulates drought and salt tolerance in rice via stomatal aperture control. Genes & development **23**:1805-1817.

**Krishnan, A., Ambavaram, M., Batlang, U., and Pereira, A.** (2010). A resource for systems analysis of transcriptional modules involved in drought response in rice. Sys Anal Stress Resp Plan **138**.

**Lee, D.K., Chung, P.J., Jeong, J.S., Jang, G., Bang, S.W., Jung, H., Kim, Y.S., Ha, S.H., Choi, Y.D., and Kim, J.K.** (2017). The rice Os NAC 6 transcription factor orchestrates multiple molecular mechanisms involving root structural adaptions and nicotianamine biosynthesis for drought tolerance. Plant Biotechnology Journal **15**:754-764.

**Li, X., Lawas, L.M., Malo, R., Glaubitz, U., Erban, A., Mauleon, R., Heuer, S., Zuther, E., Kopka, J., and Hincha, D.K.** (2015). Metabolic and transcriptomic signatures of rice floral organs reveal sugar starvation as a factor in reproductive failure under heat and drought stress. Plant, Cell & Environment **38**:2171-2192.

**Moumeni, A., Satoh, K., Kondoh, H., Asano, T., Hosaka, A., Venuprasad, R., Serraj, R., Kumar, A., Leung, H., and Kikuchi, S.** (2011). Comparative analysis of root transcriptome profiles of two pairs of drought-tolerant and susceptible rice near-isogenic lines under different drought stress. BMC plant biology **11**:1-17.

**Ning, Y., Xie, Q., and Wang, G.-L.** (2011). OsDIS1-mediated stress response pathway in rice. Plant Signaling & Behavior **6**:1684-1686.

**Peleg, Z., Reguera, M., Tumimbang, E., Walia, H., and Blumwald, E.** (2011). Cytokinin‐mediated source/sink modifications improve drought tolerance and increase grain yield in rice under water‐stress. Plant Biotechnology Journal **9**:747-758.

**Pueffeld, M., Raorane, ML., Sreenivasulu, N., Kohli, A.** (2016). Transcriptome profile for spikelets of rice plants subjected to severe reproductive stage drought stress.

**Pueffeld M, R.M., Sreenivasulu N, Kohli A** (2016). Transcriptome profile for leaves of rice plants subjected to severe reproductive stage drought stress.

**Sen SK, D.A., Kaur R, Yadav S** (2016). Global expression analysis of differential expression of genes working under Soil Water Stress condition in different genotypes of rice in Vegetative and Grain-filling stage.

**Shim, J.S., Oh, N., Chung, P.J., Kim, Y.S., Choi, Y.D., and Kim, J.-K.** (2018). Overexpression of OsNAC14 improves drought tolerance in rice. Frontiers in plant science **9**:310.

**Silveira, R., Abreu, F., Mamidi, S., McClean, P., Vianello, R., Lanna, A., Carneiro, N., and Brondani, C.** (2015). Expression of drought tolerance genes in tropical upland rice cultivars (Oryza sativa). Embrapa Milho e Sorgo-Artigo em periódico indexado (ALICE).

**Singh, S., Kumar, A., Panda, D., Modi, M.K., and Sen, P.** (2020). Identification and characterization of drought responsive miRNAs from a drought tolerant rice genotype of Assam. Plant Gene **21**:100213.

**Yang, S., Xu, K., Chen, S., Li, T., Xia, H., Chen, L., Liu, H., and Luo, L.** (2019). A stress-responsive bZIP transcription factor OsbZIP62 improves drought and oxidative tolerance in rice. BMC plant biology **19**:1-15.

**Yoo, Y.-H., Nalini Chandran, A.K., Park, J.-C., Gho, Y.-S., Lee, S.-W., An, G., and Jung, K.-H.** (2017). OsPhyB-mediating novel regulatory pathway for drought tolerance in rice root identified by a global RNA-Seq transcriptome analysis of rice genes in response to water deficiencies. Frontiers in Plant Science **8**:580.

**Yu, B., Liu, J., Wu, D., Liu, Y., Cen, W., Wang, S., Li, R., and Luo, J.** (2020). Weighted gene coexpression network analysis-based identification of key modules and hub genes associated with drought sensitivity in rice. BMC plant biology **20**:1-21.

**Zhang, F., Zhou, Y., Zhang, M., Luo, X., and Xie, J.** (2017). Effects of drought stress on global gene expression profile in leaf and root samples of Dongxiang wild rice (Oryza rufipogon). Bioscience Reports **37**.

**Zhang, J., Zhang, H., Srivastava, A.K., Pan, Y., Bai, J., Fang, J., Shi, H., and Zhu, J.-K.** (2018). Knockdown of rice microRNA166 confers drought resistance by causing leaf rolling and altering stem xylem development. Plant physiology **176**:2082-2094.
